# SR9009 inhibits lethal prostate cancer subtype 1 by regulating the LXRα/FOXM1 pathway independently of REV-ERBs

**DOI:** 10.1038/s41419-022-05392-6

**Published:** 2022-11-10

**Authors:** Hang Xu, Jiapeng Zhang, Xiaonan Zheng, Ping Tan, Xingyu Xiong, Xianyanling Yi, Yang Yang, Yan Wang, Dazhou Liao, Hong Li, Qiang Wei, Jianzhong Ai, Lu Yang

**Affiliations:** 1 Department of Urology, West China Hospital, Sichuan University, 610041 Chengdu, China; 2 Institute of Urology, West China Hospital, Sichuan University, 610041 Chengdu, China; 3 Animal Experimental Center, West China Hospital, Sichuan University, 610041 Chengdu, China; 4 Research Core Facility, West China Hospital, Sichuan University, 610041 Chengdu, China

**Keywords:** Prostate cancer, Drug development

## Abstract

Perturbations of the circadian clock are linked to multiple diseases, including cancers. Pharmacological activation of REV-ERB nuclear receptors, the core components of the circadian clock, has antitumor effects on various malignancies, while the impact of SR9009 on prostate cancer (PCa) remains unknown. Here, we found that SR9009 was specifically lethal to PCa cell lines but had no cytotoxic effect on prostate cells. SR9009 significantly inhibited colony formation, the cell cycle, and cell migration and promoted apoptosis in PCa cells. SR9009 treatment markedly inhibited prostate cancer subtype 1 (PCS1), the most lethal and aggressive PCa subtype, through FOXM1 pathway blockade, while it had no impacts on PCS2 and PCS3. Seven representative genes, including FOXM1, CENPA, CENPF, CDK1, CCNB1, CCNB2, and BIRC5, were identified as the shared genes involved in the FOXM1 pathway and PCS1. All of these genes were upregulated in PCa tissues, associated with worse clinicopathological outcomes and downregulated after SR9009 treatment. Nevertheless, knockdown or knockout of REV-ERB could not rescue the anticancer effect of SR9009 in PCa. Further analysis confirmed that it was LXRα rather than REV-ERBs which has been activated by SR9009. The expression levels of these seven genes were changed correspondingly after LXRα knockdown and SR9009 treatment. An in vivo study validated that SR9009 restrained tumor growth in 22RV1 xenograft models and inhibited FOXM1 and its targeted gene expression. In summary, SR9009 can serve as an effective treatment option for highly aggressive and lethal PCS1 tumors through mediating the LXRα/FOXM1 pathway independently of REV-ERBs.

## Introduction

Prostate cancer (PCa) is the most common malignancy in the male population, accounting for 26% of all estimated new cases and 11% of all estimated deaths in the United States in 2021 [1]. Androgen deprivation therapy (ADT) is the most commonly used treatment strategy for hormone-sensitive nonmetastatic and metastatic PCa, but the disease will eventually progress to castration-resistant prostate cancer (CRPC). Although several novel drugs, including enzalutamide [2] and abiraterone acetate [3], have been introduced to treat mCRPC patients, therapy resistance still occurs rapidly [4], hampering the therapeutic options currently available for patients. Therefore, finding new targets and therapeutic drugs for mCRPC patients has become an urgent unmet need.

The circadian clock plays a critical role in regulating physiological processes in humans [5]. Disruption of the circadian clock can lead to multiple problems, ranging from sleep disorders to cancer [6]. For example, night-shift work, which perturbs the circadian rhythm, is an important risk factor for the development of PCa [7]. Thus, pharmacological regulation of the circadian clock might be an attractive method for cancer prevention and therapies. At the molecular level, the mammalian circadian clock is composed of multiple genes that form clock-activator and clock-repressor complexes [8]. The nuclear receptors REV-ERBα (NR1D1) and REV-ERBβ (NR1D2) are the core members of the circadian clock and function as repressors of functions such as circadian rhythm (repressing BMAL1/CLOCK complexes) and metabolism [9] in the absence of a transcriptional activation domain. SR9009, a putative synthetic agonist of REV-ERBs, is beneficial for treating obesity, diabetes and circadian rhythm disorders [8] and can be easily acquired (https://www.simplyanabolics.com/sarms/sr9009-stenabolic/). Recent work by Sulli and colleagues suggested that SR9009 has powerful antitumor effects on multiple cancer types, including brain cancer, leukemia, breast cancer, colon cancer and melanoma [10]. The therapeutic effects of SR9009 on glioblastoma [11], hepatocellular carcinoma [12] and lung cancer [13] were subsequently demonstrated. However, whether SR9009 has an antitumor effect on PCa, especially lethal tumors, remains unknown.

Although most SR9009-related studies have demonstrated that the therapeutic effect of SR9009 occurs via REV-ERBs, several reports have also revealed that SR9009 might also work in a REV-ERB-independent manner [14, 15].

Herein, we sought to investigate the influence of SR9009 on PCa progression. Our results revealed that SR9009 has cytotoxic effects on PCa cell lines but not on prostate cells. Furthermore, SR9009 could inhibit prostate cancer subtype 1 (PCS1), the most lethal and aggressive PCa [16], by mediating the LXRα/FOXM1 pathway independently of REV-ERBs. The results obtained provide new insight into molecular mechanisms and therapeutic interventions in PCa.

## Materials and methods

### Cell lines

RWPE-1, PC3, 22RV1, DU145, LNCaP, and C4-2B cells were purchased from Shanghai Cell Bank Type Culture Collection Committee (CBTCCC, Shanghai, China). HEK293T cell lines were purchased from the American Type Culture Collection (ATCC, Manassas, Virginia, USA). PCa cell lines were cultured in RPMI 1640 medium (HyClone, Utah, USA) supplemented with 10% fetal bovine serum (FBS; Gibco, Australia) and 1% antibiotics (penicillin and streptomycin; HyClone) in a humidified incubator containing 5% CO_2_ at 37 °C. RWPE-1 cells were cultured in keratinocyte serum-free medium. Cells stably transfected with plasmid were cultured in complete culture medium with additional puromycin (2 μg/mL; KEHBIO, Beijing, China).

### Cell transfection

Small interfering RNAs (siRNAs) targeting REV-ERBα, REV-ERBβ, LXRα, and FOXM1 were obtained from RiboBio (Guangzhou, China). The siRNA sequence is shown in Table S1. siRNA (50 nM) was transfected into cells using Lipofectamine 3000 transfection reagent (Invitrogen, CA, USA). FOXM1 overexpression plasmid (pLV.CMV.FOXM1.PGK. Puro) was purchased from PackGene (Guangzhou, China). The lentiviral packaging procedure for the target plasmid has been described previously [17].

### Clustered regularly interspaced short palindromic repeats (CRISPR)/Cas9-mediated gene editing

Cas9-expressing stable cell lines were constructed by infection with Cas9 lentivirus and further puromycin screening. REV-ERB (NR1D1 and NR1D2)-specific sgRNA oligos were designed and cloned into the pLentiCRISPR V2 plasmid (sequences listed in Table S2). The harvested lentivirus was added to the cell supernatant and centrifuged at 2000 rpm for 1 h, followed by incubation for 1.5 h.

### Cell counting kit-8 (CCK-8) assay

SR9009, purchased from MedChemExpress (MCE, NJ, USA), was first dissolved in dimethyl sulfoxide (DMSO) and then diluted to the working concentrations (maximum DMSO concentration < 0.5%). Approximately (2–5) × 10^3^ cells per well were seeded in a 96-well plate. When the cells grew to 70–80% confluence, they were treated with SR9009, DMSO, or siRNAs for 48 h. Thereafter, the culture medium in each well was replaced with 100 μL of fresh complete culture medium containing 10 μL of CCK-8 reagent (Dojindo Molecular Technologies, Rockville, USA). Then, the 96-well plate was placed into an incubator at 37 °C in the dark for 2 h. Finally, the plate was placed in the EonTM Microplate Reader (Bio-Tek, VT, USA) to measure the absorbance at 450 nm. At least 3 duplicate wells were set at the same time.

### Colony formation assay

PCa cells were seeded in 6-well plates at 500 cells/well. After 48 h of incubation, the cells were treated with SR9009 (20 μM) or DMSO for another 10–14 days. The cells were washed with phosphate buffered saline (PBS) and fixed with cold methanol for 20 min. After washing with PBS, the cells were stained with crystal violet (Beyotime, Shanghai, China) for 15 min. Subsequently, the cells were washed and imaged using a Celigo Imaging Cytometer.

### Cell cycle

Cells were pretreated with SR9009 (20 μM), DMSO or siRNAs for 48 h. Subsequently, they were digested, centrifuged and collected into flow tubes. Then, 500 μL of 70% ice-cold ethanol was added, and the cells were fixed overnight at 4 °C. Cells were washed and filtered before the PI/RNase A (KeyGen Biotech, Jiangsu, China) dye working solution was added. After 30 min of incubation in the dark, we detected and recorded the cell cycle using a CytoFLEX Research Flow Cytometer (Beckman Coulter, CA, USA).

### Cell apoptosis

An Annexin V-PE/7-AAD cell apoptosis detection kit was purchased from KeyGen Biotech. Cells were treated with SR9009 (20 μM) or DMSO for 48 h. The cells were then digested and washed twice with cold PBS. Next, 55 μL dye working solution (50 μL binding buffer + 5 μL 7-AAD) was added to the cells and incubated at 37 °C for 5–15 min in the dark. Subsequently, 450 μL binding buffer and 1 μL Annexin V-PE were added and incubated for 5–15 min. Cell apoptosis was assessed by a FACSAria SORP instrument (BD, USA).

### Wound healing assay

Cells were digested, suspended and seeded at 3 × 10^5^ cells per well in 6-well plates. When the cells grew to approximately 80–90% confluence, 3 vertical parallel lines were drawn in each well. Cells were washed twice and treated with SR9009 (20 μM) or DMSO for 24 h. Images were immediately taken under an inverted fluorescence Zeiss OBSERVER D1/AX10 CAM HRC microscope (Zeiss), and the sites were recorded. Subsequently, the 6-well plates were placed in the cell incubator for an additional 24 h of incubation and imaged again. ImageJ software (National Institutes of Health, USA; Version 1.48) was applied to calculate the migration distance.

### Transwell assay

Transwell migration assays were conducted using a Transwell chamber (Millipore, Massachusetts, USA). Briefly, Transwell chambers were placed on a 24-well plate. Fresh medium containing 10% FBS and 20 μM SR9009 in 600 μL was added to the lower chambers, and (2–5) × 10^4^ cells in 200 μL of medium containing 20 μM SR9009 without FBS were added to the upper chamber. The 24-well plate was incubated at 37 °C for 48 h. Cells that invaded through the chamber were washed, fixed (20 min with 4% paraformaldehyde) and stained (30 min with crystal violet). Then, the upper chambers were washed, photographed and preserved under an inverted fluorescence OBSERVER D1/AX10 cam HRC microscope (Zeiss). Transferred cells were analyzed using ImageJ software.

### RNA-sequencing (RNA-seq)

PC3 cells were treated with 20 μM SR9009 or DMSO for 48 h. Then, the cells were harvested, and RNA was stored using TRIzol (Invitrogen, CA, USA). Novogene (Beijing, China) was entrusted to perform RNA-seq. Briefly, RNA samples were extracted, and RNA sample quantification and qualification were performed. Then, the NEBNext® UltraTM RNA Library Prep Kit for Illumina® (NEB, USA) was selected to generate sequencing libraries following the manufacturer’s recommendations, and index codes were added to attribute sequences to each sample. After clustering and sequencing (Novogene Experimental Department), data analysis was performed through the following steps: quality control, read mapping to the reference, and quantification of the gene expression level (fragments per kilobase million was calculated). Differential expression analysis was performed using the DESeq2 R package (1.16.1). Gene Ontology (GO) enrichment analysis and Kyoto Encyclopedia of Genes and Genome (KEGG) enrichment analysis were implemented by the clusterProfiler R package.

### RNA extraction and real-time quantitative polymerase chain reaction (qPCR)

Total RNA was extracted by using the RNeasy Mini Kit (Qiagen, Texas, USA) according to manual protocol. A total of 1 μg of RNA was added to synthesize first-strand complementary DNA (cDNA) using the Thermo Scientific RevertAid RT kit (Vilnius, Lithuania) with Oligo (dT)_18_. Quantitative PCR (qPCR) was performed using the QuantiNova SYBR Green PCR kit (Qiagen), and reactions were performed on the CFX96 Touch Real-Time PCR System (Bio-Rad, California, USA). The PCR amplification settings were as follows: 50 °C for 2 min and 95 °C for 10 min; 40 cycles of 98 °C for 5 s; and 59 °C for 10 s. β-actin was used for normalization, and each sample was repeated at least three times. The data were analyzed using the 2^−ΔΔCt^ method. Primer sequences were acquired from the PrimerBank website (https://pga.mgh.harvard.edu/primerbank/) and synthesized by Sangon Biotech (Shanghai, China). The primers used in this study are shown in Table S2.

### Western blot analysis

Proteins were extracted, and the concentrations were determined using the Pierce™ BCA Protein Assay Kit (Thermo). Proteins were denatured at 100 °C for 10 min. After the proteins were separated by sodium dodecyl sulfate‒polyacrylamide gel electrophoresis (SDS‒PAGE, Epizyme, Shanghai, China), the gels were transferred onto polyvinylidene fluoride (PVDF) membranes (Millipore) and run at 250 mA for 90 min. Subsequently, the membranes were cut, blocked (5% skim milk powder), and incubated with diluted primary antibodies at 4 °C overnight. The primary antibodies were as follows: anti-GAPDH (ZEN-BIO 200306-7E4), anti-β-actin (ZEN-BIO 250132), anti-vinculin (ZEN-BIO R26085), anti-NR1D1 (ab174309), anti-NR1D2 (ab251948 and Protein Tech 13906-1-AP), anti-FOXM1 (CST20459), anti-LXRα (ab41902), anti-CCNB1 (CST12231), anti-CCNB2 (ab185622), anti-CENPA (CST2186), anti-CENPF (CST58982), anti-CDK1 (ZEN-BIO 200544), anti-survivin (CST2808), and anti-ARNTL (Protein Tech 14268-1-AP). The membranes were washed and incubated with secondary antibodies for 1 h according to the primary antibody sources. Immunoreactivity was visualized using enhanced chemiluminescent (ECL) chromogenic substrate (Millipore). The membranes were finally detected by using a ChemiDoc MP Imager System (Bio-Rad).

### Immunohistochemistry (IHC)

Specimens were fixed in 4% paraformaldehyde at room temperature and embedded in paraffin. Then, tissues were cut into 4 μm thick sections. Subsequently, we dewaxed, hydrated and incubated the tissues with antibodies overnight at 4 °C. After incubation with the corresponding secondary antibodies, the sections were stained with diaminobenzidine and reverse stained with hematoxylin.

### Mouse model

Male nude BALB/c mice (18–20 g each) at 6 weeks of age were purchased from Chengdu Dossy Experimental Animals Co., Ltd. (Chengdu, China). Mice were castrated with goserelin (MCE, daily for 19 days) subcutaneously. At the same time, cultured 22RV1 cells were collected and suspended in PBS. A 100 μL cell suspension with 5 × 10^6^ cells was subcutaneously injected into the right flank of the mouse to establish a subcutaneous xenograft model. The weight and tumor volume of the mice were measured every 3 days, and the formula for calculating volume was (length × width^2^)/2 [18]. When the tumor volume increased up to 100–200 cm^3^, the mice were randomly divided into two groups (with 4 mice in each group). The investigator was not blinded to the group allocation during the whole experiment. SR9009 was dissolved in 15% Cremophor and administered twice daily (100 mg/kg) [10] in the experimental group through intraperitoneal injection, and the control group was given the same volume of Cremophor. When there was a significant difference between the two groups or the tumor volume exceeded 1000 cm^3^, mice were sacrificed, and subcutaneous tumors were harvested for hematoxylin-eosin staining and IHC.

### Bioinformatic analysis

GEPIA2 (http://gepia2.cancer-pku.cn/#index) was used to analyze the gene expression differences between normal and tumor samples in prostate adenocarcinoma in The Cancer Genome Atlas (TCGA-PRAD). GEPIA2 was also applied to analyze the associations of gene expression (median cutoff value) and disease-free survival (DFS). mRNA expression Z scores relative to diploid samples were analyzed using cBioPortal (http://www.cbioportal.org). Protein levels of LXRα and FOXM1 were obtained from *The Human Protein Atlas*. The level 3 HTSeq-FPKM data in TCGA-PRAD were downloaded from https://portal.gdc.cancer.gov/. RNA-seq data in FPKM format were transformed into transcripts per million reads (TPM) format with log_2_ transformation. Correlation analyses were performed using the R package ggplot2.

### Statistical analysis

Data are presented as the mean ± standard deviation (SD) of at least three independent experiments. Data were statistically analyzed using GraphPad Prism software (Version 6.02; CA, USA). Student’s *t* test, ANOVA, or Wilcoxon rank sum test were applied as appropriate. Homogeneity of variance was tested using the Shapiro‒Wilk normality test for equality of variances. A two-tailed *P* value lower than 0.05 indicated statistical significance, which was labeled as follows: *P < 0.05, **P < 0.01, ***P < 0.001, ****P < 0.0001.

## Results

### SR9009 was specifically lethal to PCa cell lines

**Fig. 1:**
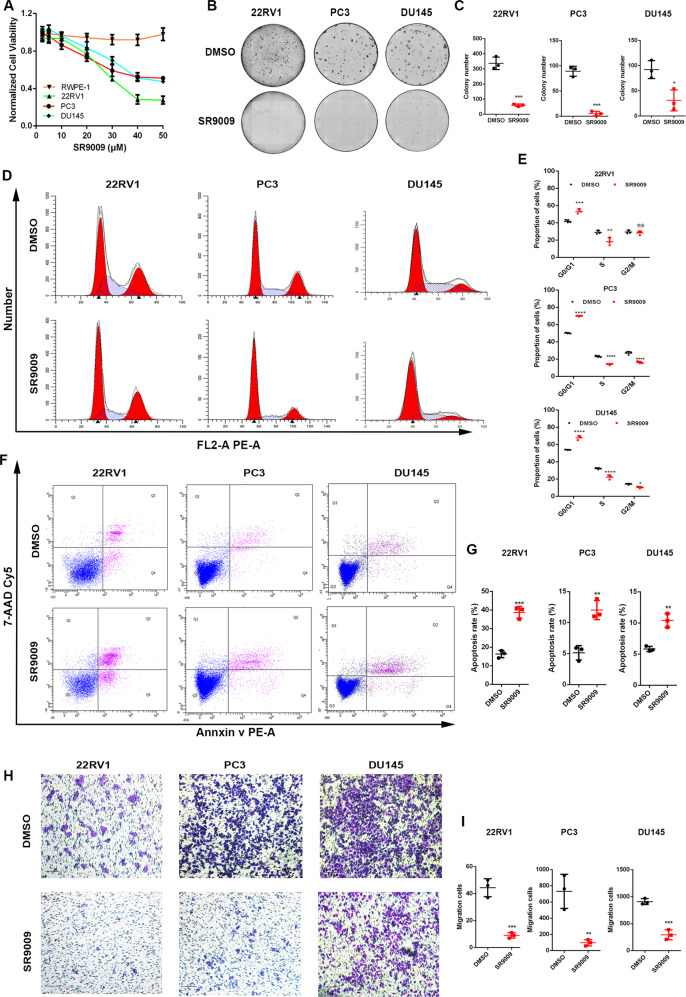
SR9009 is selectively lethal in PCa cells. **A** CCK-8 assay of RWPE-1, PC3, 22RV1, and DU145 cell lines treated with different concentrations of SR9009 for 48 h; means ± SDs; unpaired *t* test. **B**, **C** Clone formation assay of PC3, 22RV1, and DU145 cell lines treated with DMSO or SR9009 (20 μM) for 7–14 days; *n* = 3; means ± SDs; unpaired *t* test. **D**, **E** Cell cycle tests of PC3, 22RV1, and DU145 cells treated with DMSO or SR9009 (20 μM) for 48 h; *n* = 3; means ± SDs; ANOVA. **F**, **G** Cell apoptosis tests of PC3, 22RV1, and DU145 cells treated with DMSO or SR9009 (20 μM) for 48 h; *n* = 3; means ± SDs; unpaired *t* test. **H**, **I** Transwell migration assays of PC3, 22RV1, and DU145 cells treated with DMSO or SR9009 (20 μM) for 48 h; *n* = 3; means ± SDs; unpaired *t* test. ns, not significant; *p < 0.05; **p < 0.01; ***p < 0.001; ****p < 0.0001.

We assessed the impact of SR9009 on cell viability in three PCa cell lines and 1 normal prostate cell line. SR9009 markedly inhibited 22RV1, PC3 and DU145 cell viability in a dose-dependent manner but had no impact on RWPE-1 cells (Fig. 1A, Fig. S1A–D). We further showed that SR9009 significantly inhibited colony formation (Fig. 1B, C) and induced cell cycle arrest in PCa cells (Fig. 1D, E). SR9009 also promoted apoptosis in PCa cells compared with controls (Fig. 1F, G). Moreover, SR9009 impaired PCa cell migration in vitro in scratch and Transwell assays (Fig. 1H, I, Fig. S1D–E). These results indicated that SR9009 could inhibit PCa cell growth and migration but had no influence on normal prostate cells in vitro.

### SR9009 inhibited the cell cycle pathway and suppressed lethal PCS1

**Fig. 2:**
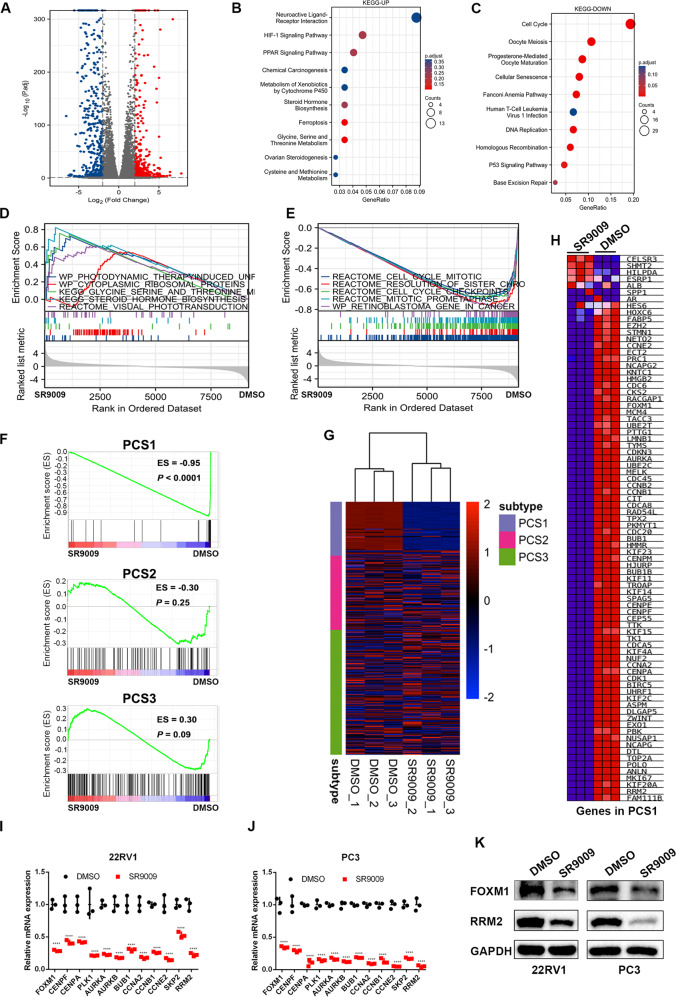
SR9009 downregulated the cell cycle pathway and suppressed lethal prostate cancer subtype 1 (PCS1). **A** Volcano plot of differentially expressed genes between the SR9009 group (*n* = 3) and DMSO group (*n* = 3) after RNA sequencing. **B**, **C** Kyoto Encyclopedia of Genes and Genome (KEGG) pathway analysis of up- (**B**) and downregulated (**C**) pathways after SR9009 or DMSO was added; **D**, **E** Gene Set Enrichment Analysis (GSEA) analysis of the top 5 upregulated (**D**) and downregulated (**E**) pathways applying “c2.cp.v7.2.symbols.gmt [Curated]” from the MSigDB database. **F** GSEA of PCS1 after SR9009 or DMSO administration. **G** Cluster analysis of genes in PCS1, PCS2, and PCS3 between the SR9009 and DMSO groups. **H** Heatmap of genes in PCS1 after SR9009 or DMSO administration. **I**, **J** qPCR validations of genes downregulated in PCS1 in 22RV1 (**I**) and PC3 (**J**); *n* = 3; means ± SDs; ANOVA. **K** Western blot validation of SR9009 treatment on the expression of FOXM1 and RRM2 in PC3 and 22RV1 cells. ns, not significant; *p < 0.05; **p < 0.01; ***p < 0.001; ****p < 0.0001.

A total of 559 upregulated and 553 downregulated genes were identified through RNA-seq (Fig. 2A, |Log2FC|> 2, adjusted *P* value < 0.05). KEGG pathway enrichment analysis revealed that SR9009 significantly promoted ferroptosis and glycine, serine and threonine metabolism (Fig. 2B) and inhibited the cell cycle pathway (Fig. 2C). GSEA indicated that SR9009 mainly functioned by regulating the PCa cell cycle (Fig. 2D, E). A recent analysis classified PCa into three distinct subtypes, named the “PCS classification” [16], and this classification performed better in distinguishing luminal and basal PCa than the PAM50 classification [19]. Our subsequent GSEA found that SR9009 markedly inhibited PCS1, the most aggressive and lethal PCa subtype (enrichment score [ES] = −0.95, *P* < 0.0001), whereas it had no influence on PCS2 (ES = −0.30, *P* = 0.25) and PCS3 (ES = 0.30, *P* = 0.09) when compared with the control groups (Fig. 2F–H, Fig. S2A, B). The results indicated that SR9009 significantly reduced 71 of 82 genes in PCS1 (Fig. 2H). The sequencing results were validated at the mRNA and protein levels (Fig. 2I–K).

### SR9009 inhibited PCS1 by downregulating FOXM1 expression

**Fig. 3:**
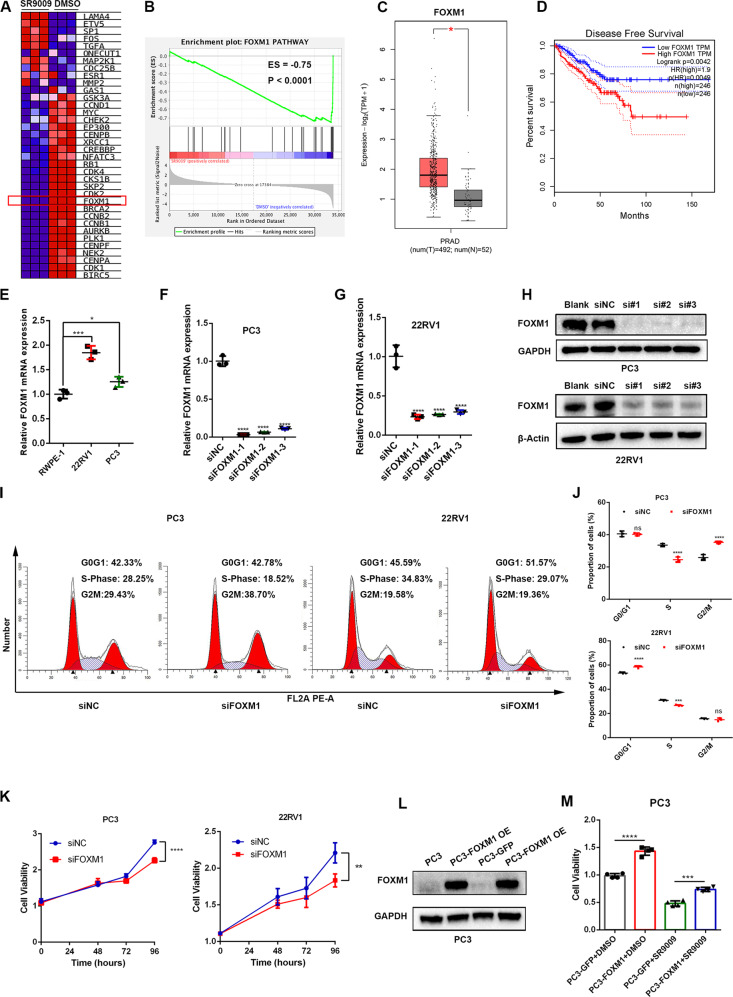
SR9009 inhibited PCS1 by downregulating FOXM1 expression. **A** Heatmap of genes in the FOXM1 pathway after SR9009 or DMSO administration. **B** GSEA of the FOXM1 pathway after SR9009 or DMSO administration. **C** Expression of FOXM1 between normal and tumor samples in TCGA-PRAD using the GEPIA database. **D** Association of FOXM1 expression and disease-free survival using the GEPIA database. **E** Relative expression of PC3 and 22rv1 compared with RWPE-1-cell lines. **F**, **G** qPCR validation of the knockdown of FOXM1 in PC3 and 22RV1 cell lines; *n* = 3; means ± SDs; ANOVA. **H** Western blot validation of the knockdown of FOXM1 in PC3 and 22RV1 cell lines. **I**, **J** Flow cytometry of cell cycle analysis of siFOXM1 in PC3 and 22RV1 cells after 48 h of transfection; *n* = 3; means ± SDs; ANOVA. **K**: CCK-8 assays of the effect of siFOXM1 on cell viability in PC3 (*n* = 6) and 22RV1 (*n* = 5) cells; means ± SDs; ANOVA. **L** Western blot of FOXM1 overexpression validation in PC3 cells. OE overexpression. **M** SR9009 (20 μM)-induced cytotoxicity (48 h incubation) could be partially rescued by FOXM1 overexpression; *n* = 4; means ± SDs; unpaired *t* test. ns not significant; *p < 0.05; **p < 0.01; ***p < 0.001; ****p < 0.0001.

Previous studies have demonstrated that the FOXM1 pathway is the master driver of PCS1 [20]. We found that SR9009 treatment significantly reduced 20 of 36 FOXM1-regulated genes (Fig. 3A). GSEA also validated that SR9009 treatment inhibited the FOXM1 pathway (ES = −0.75, *P* < 0.0001, Fig. 3B). Subsequently, from the TCGA-PRAD dataset, we found that FOXM1 was significantly overexpressed in PCa samples compared with normal tissues (Fig. 3C), and the high expression of FOXM1 was associated with worse DFS (Fig. 3D). The mRNA expression of FOXM1 was found to be higher in 22RV1 and PC3 cells than in RWPE-1 cells (Fig. 3E). Knocking down FOXM1 in PC3 and 22RV1 cell lines (Fig. 3F–H) significantly induced cell cycle arrest (Fig. 3 I,J) and reduced cell viability (Fig. 3K). FOXM1 overexpression (Fig. 3L) increased cell viability in PC3 cells, and the treatment effect of SR9009 could be partially rescued after FOXM1 overexpression (Fig. 3M). Together, these results indicated that SR9009 mediated PCS1 inhibition through FOXM1 regulation.

### SR9009 inhibited the expression of seven genes involved in both the FOXM1 pathway and lethal PCS1

**Fig. 4:**
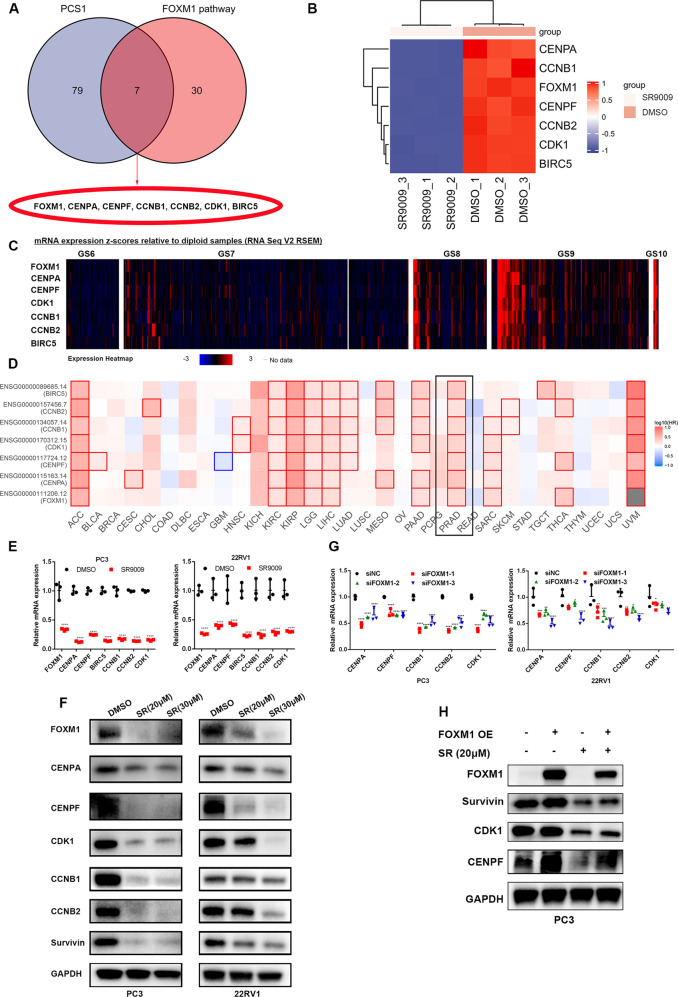
SR9009 treatment inhibited the expression of genes in the FOXM1 pathway and PCS1. **A** Venn diagram of the common genes in both the FOXM1 pathway and PCS1. **B** Heatmap of FOXM1, CENPA, CENPF, CDK1, CCNB1, CCNB2, and BIRC5 expression based on RNA sequencing. **C** mRNA expression of FOXM1, CENPA, CENPF, CDK1, CCNB1, CCNB2, and BIRC5 relative to diploid samples from TCGA-PRAD analysis (cBioPortal database). **D** Association of FOXM1, CENPA, CENPF, CDK1, CCNB1, CCNB2, and BIRC5 with disease-free survival across cancers (GEPIA database). PRAD is shown in the black frame. **E**, **F** qPCR validation of the expression of these seven genes after SR9009 treatment for 48 h; *n* = 3; means ± SDs; ANOVA. **F** Western blot validation of the expression of these seven genes after SR9009 treatment for 48 h. **G** qPCR results of the effect of FOXM1 knockdown on CENPA, CENPF, CDK1, CCNB1, and CCNB2 expression; *n* = 3; means ± SDs; ANOVA. **H** Effect of FOXM1 overexpression and/or SR9009 treatment on the expression of FOXM1, CENPA, CENPF, CDK1, CCNB1, CCNB2, and BIRC5. ns, not significant; *p < 0.05; **p < 0.01; ***p < 0.001; ****p < 0.0001.

To further identify the key genes involved in lethal PCS1, we obtained 7 genes, including FOXM1, CENPA, CENPF, CCNB1, CCNB2, CDK1 and BIRC5, after intersection between PCS1 genes and FOXM1 pathway-involved genes (Fig. 4A). The expression of these 7 genes was significantly downregulated after SR9009 treatment (Fig. 4B). Previous studies have validated the important role of these 7 genes in promoting PCa progression [21**–**25]. Our bioinformatic analyses showed that the high expression levels of these 7 genes were all associated with adverse clinicopathological outcomes in PCa (Fig. S3A-D). We show the expression heatmap of these 7 genes stratified by Gleason score in Fig. 4C (with the Gleason score increased, the expression of these 7 genes was elevated). Moreover, the high expression of these 7 genes was associated with decreased DFS in PCa and other cancer types (Fig. 2D, Fig. 4D, Fig. S3E–J). SR9009 significantly decreased the expression of these 7 genes at both the mRNA and protein levels (Fig. 4E, F). Consistent with the fact that FOXM1 directly regulates the other 6 genes, we observed reduced expression of these 6 genes after FOXM1 knockdown (Fig. 4G). More importantly, the decreased expression of FOXM1 target genes induced by SR9009 could be partially rescued after FOXM1 overexpression (Fig. 4H). These results indicated that SR9009 could inhibit FOXM1-related genes and serve as a FOXM1 pathway inhibitor.

### SR9009-induced FOXM1 pathway inhibition was independent of REV-ERBs

**Fig. 5:**
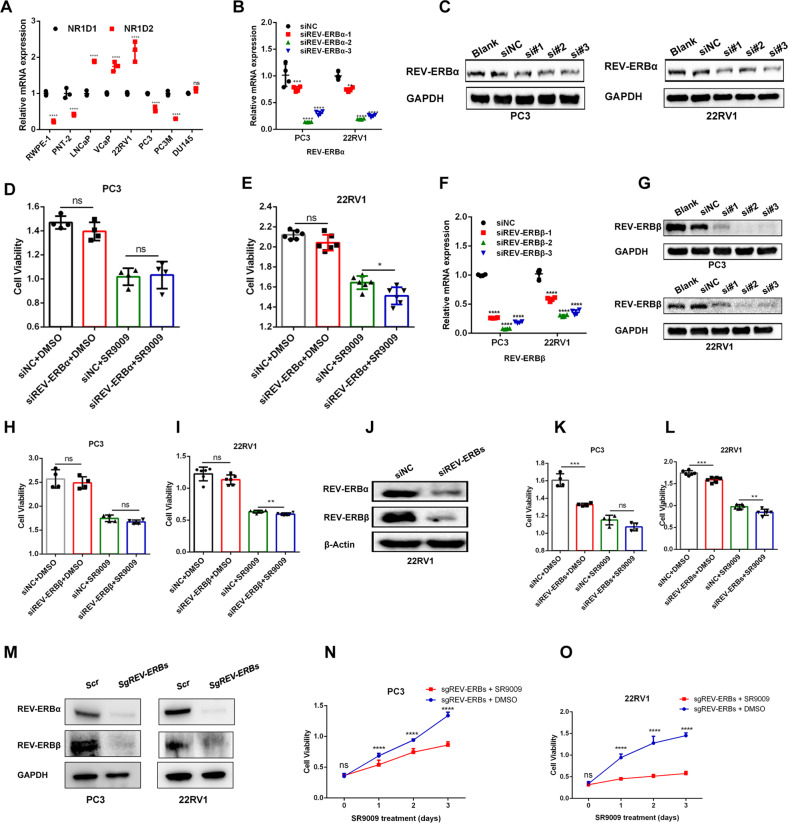
SR9009-induced FOXM1 pathway inhibition was independent of REV-ERBs. **A** Relative mRNA expression levels of REV-ERBα and REV-ERBβ across eight prostate cell lines; *n* = 3; means ± SDs; ANOVA. **B** qPCR of REV-ERBα knockdown validation in PC3 and 22RV1 cells using 3 siRNAs; *n* = 4; means ± SDs; ANOVA. **C** Western blot validation of REV-ERBα knockdown in PC3 and 22RV1 cells using 3 siRNAs. **D**, **E** CCK-8 assay showed that knockdown of REV-ERBα could not rescue SR9009-induced cytotoxicity in PC3 (**D**; *n* = 4; means ± SDs; unpaired *t* test) and 22RV1 (**E**; *n* = 6; means ± SDs; unpaired *t* test) cells. **F**, **G** qPCR (**F**; *n* = 4; means ± SDs; ANOVA) and Western blot (**G**) of REV-ERBβ knockdown validation in PC3 and 22RV1 cells using 3 siRNAs. **H**, **I** CCK-8 assay showed that knockdown of REV-ERBβ could not rescue SR9009-induced cytotoxicity in PC3 (**H**; *n* = 4; means ± SDs; unpaired *t* test) and 22RV1 (**I**; *n* = 6; means ± SDs; unpaired *t* test) cells. **J** Western blot of REV-ERB knockdown validation in 22RV1 cells. **K**, **L** CCK-8 assay showed that knockdown of REV-ERBs could not rescue SR9009-induced cytotoxicity in PC3 (**K**; *n* = 4; means ± SDs; unpaired *t* test) and 22RV1 (**L**; *n* = 6; means ± SDs; unpaired *t* test) cells. **M** Western blot validation of REV-ERB protein levels through CRISPR/Cas9 gene editing in PC3 and 22RV1 cells. N-O: PC3 (**N**, *n* = 8 means ± SDs; ANOVA) and 22RV1 (**O**, *n* = 8 means ± SDs; ANOVA)-REV-ERB knockout cells were treated with SR9009 (20 μM) or DMSO for 3 days. ns, not significant; *p < 0.05; **p < 0.01; ***p < 0.001; ****p < 0.0001.

Given that SR9009 was reported to be an REV-ERB agonist [10] and that the activation of REV-ERBs could lead to transcriptional repression of target genes [26] (BMAL1 [also known as ARNTL] is an essential feedback loop in regulating circadian rhythms [27]), we first examined the effect of SR9009 treatment on BMAL1 expression. Surprisingly, after analyzing our RNA-seq data, we found that BMAL1 expression was elevated after SR9009 treatment (Fig. S4A). Subsequent experiments revealed that SR9009 treatment increased BMAL1 expression at both the mRNA (Fig. S4B, C) and protein levels (Fig. S4D, E). To confirm that the cytotoxic effect of SR9009 was mediated by REV-ERBs, we examined the mRNA expression of REV-ERBα and REV-ERBβ across eight prostate cell lines (Fig. 5A). We first downregulated REV-ERBα expression (Fig. 5B, C) and observed elevated BMAL1 expression (Fig. S5A). Surprisingly, the SR9009-induced decrease in cell viability was not rescued after REV-ERBα knockdown in PC3 and 22RV1 cells (Fig. 5D, E). Furthermore, we also found that FOXM1 and its target genes were not elevated after REV-ERBα silencing (Fig. S5B). These results indicated that SR9009-induced FOXM1 pathway inhibition was not mediated by REV-ERBα activation. In addition, SR9009-induced cell viability impairment was not rescued after REV-ERBβ knockdown (Fig. 5F–I, Fig. S5C), and FOXM1 expression was decreased (not elevated) after REV-ERBβ knockdown (Fig. S5D), suggesting that SR9009-induced FOXM1 pathway inhibition was not mediated by REV-ERBβ activation.

Subsequently, both REV-ERBα and REV-ERBβ were simultaneously silenced (Fig. 5J, Fig. S5E), which did not rescue the SR9009 effect on PCa cells (Fig. 5K, L) and did not elevate FOXM1 expression (Fig. S5F). Furthermore, we conducted CRISPR/Cas9-mediated REV-ERB knockout in PC3 and 22RV1 cells (Fig. S6). The western blot results revealed that REV-ERBs were efficiently disrupted in both PC3 and 22RV1 cells (Fig. 5M). Most importantly, SR9009 treatment could not rescue PCa cell viability after REV-ERB knockout (Fig. 5N, O). Taken together, the above data demonstrated that SR9009-induced FOXM1 pathway inhibition was independent of REV-ERBs.

### SR9009 inhibits the FOXM1 pathway through activation of LXRα

**Fig. 6:**
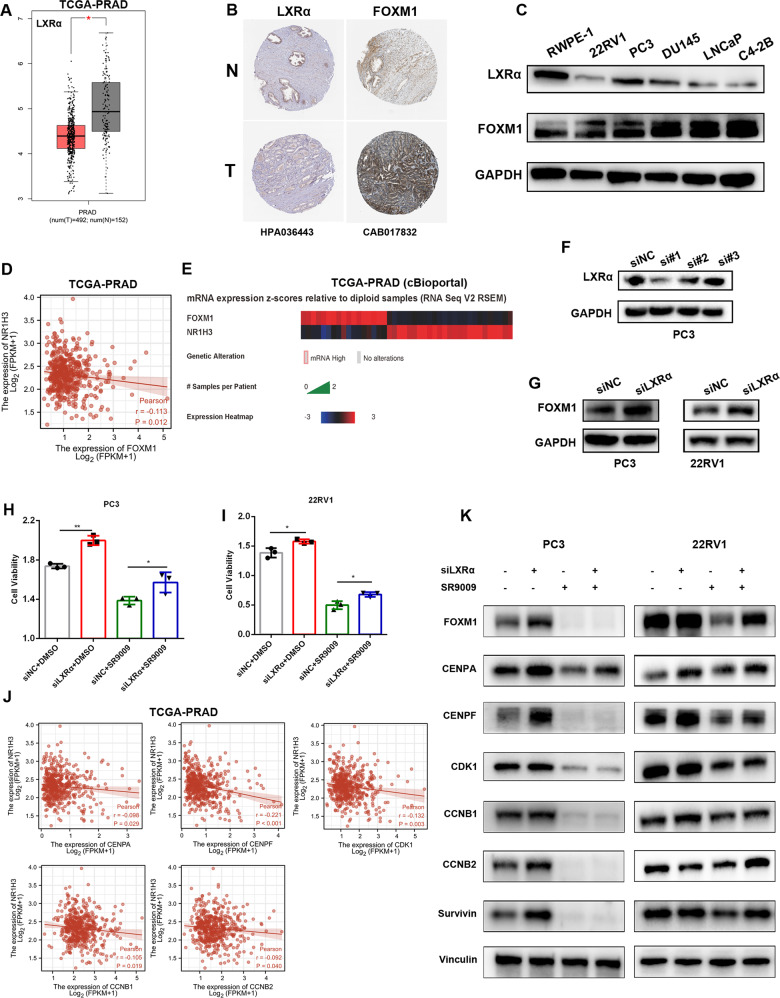
SR9009 inhibited the FOXM1 pathway through LXRα activation. **A** LXRα expression in normal and tumor tissues from TCGA-PRAD datasets (GEPIA database). **B** Protein expression of LXRα and FOXM1 in PCa tissues relative to normal prostate tissues from The Human Protein Atlas databases. **C** Protein expression of LXRα and FOXM1 across prostate cell lines. **D** A negative correlation was found between LXRα and FOXM1. **E** mRNA expression profile of LXRα and FOXM1 relative to diploid samples in TCGA-PRAD (cBioPortal). **F** Western blot of LXRα knockdown validation in PC3 cells using siRNA transfection. **G** LXRα knockdown increased FOXM1 expression in PC3 and 22RV1 cells. **H**, **I** CCK-8 assay showed that knockdown of LXRα could rescue SR9009-induced cytotoxicity in PC3 (**H**; *n* = 3; means ± SDs; unpaired *t* test) and 22RV1 cells (**I**; *n* = 3; means ± SDs; unpaired *t* test). **J** LXRα is negatively correlated with the expression of CENPA, CENPF, CDK1, CCNB1, and CCNB2 in TCGA-PRAD. **K** Effect of LXRα knockdown and/or SR9009 treatment on the expression of FOXM1, CENPA, CENPF, CDK1, CCNB1, CCNB2, and BIRC5. ns not significant; *p < 0.05; **p < 0.01; ***p < 0.001; ****p < 0.0001.

We next sought to explore other possible SR9009 targets that could repress the FOXM1 pathway. Previous literature reported that SR9009 manifested marked selectivity for LXRα (also known as NR1H3) when compared with REV-ERBα [28], and LXRα suppressed FOXM1 expression in hepatocellular carcinoma [29]. Thus, we hypothesized that SR9009-mediated FOXM1 pathway inhibition might involve the activation of LXRα. We found that LXRα mRNA and protein levels were downregulated in TCGA-PRAD tissues compared with normal tissues (Fig. 6A-B). Compared with RWPE-1, the protein expression of LXRα was decreased in PCa cell lines, whereas FOXM1 was increased (Fig. 6C). A negative association was found between LXRα and FOXM1 (Fig. 6D-E). In addition, LXRα knockdown significantly upregulated FOXM1 expression (Fig. 6F, G), suggesting that FOXM1 is regulated by LXRα. More importantly, the SR9009-induced cell viability reduction was partially rescued after LXRα knockdown (Fig. 6H, I), indicating that LXRα rather than REV-ERBs was activated after SR9009 treatment. In addition, we found that LXRα was negatively associated with FOXM1-targeted genes, including CENPA, CENPF, CDK1, CCNB1 and CCNB2, in TCGA-PRAD (Fig. 6J). LXRα knockdown upregulated FOXM1-targeted genes and partially rescued SR9009-induced FOXM1 pathway inhibition (Fig. 6K).

### SR9009 impaired PCa tumor growth and blocked the FOXM1 pathway in vivo

**Fig. 7:**
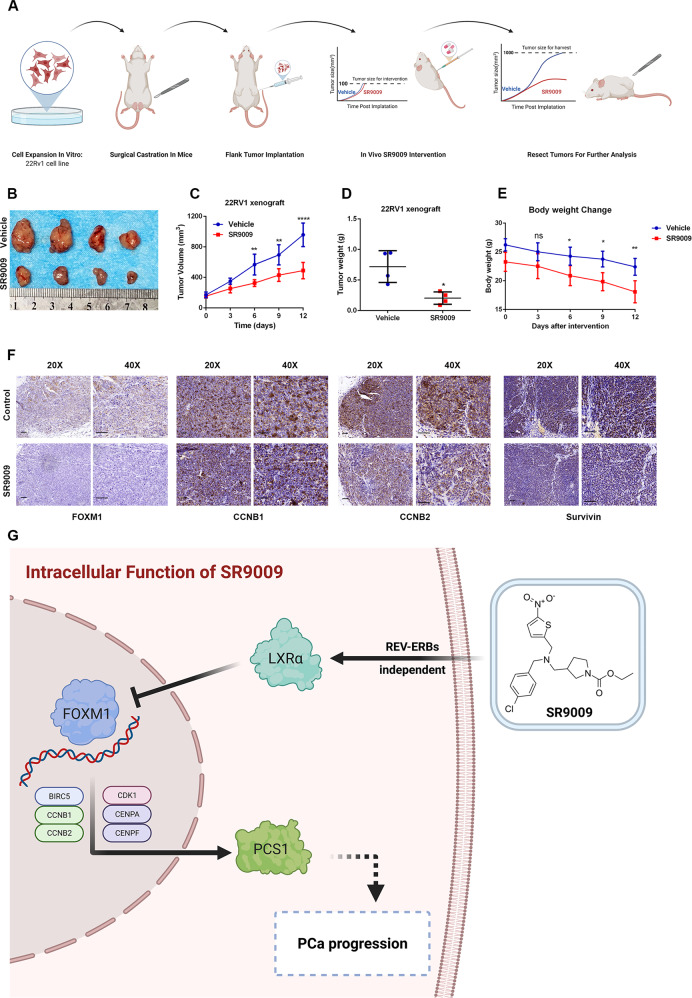
SR9009 impaired PCa tumor growth and blocked the FOXM1 pathway in vivo. **A** Experimental procedure of subcutaneous 22RV1 xenograft model building. **B** Tumor sizes were significantly lower in the SR9009 group than in the vehicle group. SR9009 was administered at a dose of 100 mg/kg twice per day via intraperitoneal injection. **C** SR9009 impaired PCa tumor growth; *n* = 4; means ± SDs; ANOVA **D** tumor weights were markedly lower in the SR9009 group than in the vehicle group; *n* = 4; means ± SDs; unpaired *t* test. **E** SR9009 reduced mouse body weight; *n* = 4; means ± SDs; ANOVA. **F** Immunohistochemistry showed that SR9009 downregulated the expression of FOXM1, CCNB1, CCNB2, and survivin. **G** Schematic model of the regulatory network of SR9009 in PCa. Data are expressed as the means ± SEMs. ns not significant; *p < 0.05; **p < 0.01; ***p < 0.001; ****p < 0.0001.

We next assessed the impact of SR9009 on PCa progression in vivo. We established 22RV1 xenografts in nude mice and treated them with SR9009, along with continuous ADT (Fig. 7A). Our results indicated that SR9009 treatment significantly decreased tumor volume (Fig. 7B) and tumor growth (Fig. 7C). Tumor weights in the SR9009 group were significantly lower than those in the control group (Fig. 7D). As expected, the mouse weights were decreased after SR9009 treatment (Fig. 7E). Furthermore, by performing IHC staining using mouse tumor tissues, we found that SR9009 treatment reduced the expression of FOXM1 and its target genes CCNB1, CCNB2 and Survivin (Fig. 7F). The findings of the study are summarized in Fig. 7G.

## Discussion

The incidence and mortality of PCa have increased in recent decades. Compared with 1990, the incidence of PCa in 2019 increased by 169.11% and caused over 480 thousand male deaths worldwide [30]. Current treatment strategies for mCRPC patients have been developed, such as androgen receptor inhibitors, PARP inhibitors, and prostate-specific membrane antigen (PSMA)-targeting therapies [31], and there is still considerable room to improve the treatment efficacy and find new drugs for PCa treatment. In this study, by conducting a series of experiments, we showed that SR9009 could significantly inhibit PCa cell growth in vitro and in vivo. SR9009 inhibited the most aggressive and lethal PCS1 tumors. Moreover, our mechanistic exploration showed that SR9009 might also function by regulating the LXRα/FOXM1 pathway independently of REV-ERBs. To the best of our knowledge, this is the first study investigating the role of SR9009 in treating PCa. We also proposed a novel SR9009-related pathway and validated that SR9009 could serve as both a PCS1 and FOXM1 inhibitor. Our study might pave the way for lethal PCa treatment.

Due to the significant inherent biological heterogeneities in cancers [32], molecular classification has been applied to guide precise treatment. Classifications of luminal A, luminal B, and basal subtypes are widely adopted in breast cancer. Current clinical practice or guidelines do not recommend a recognized classification system for PCa. Most attempts to classify PCa were based on gene signatures [33**–**36] and were limited by the sample size analyzed. Two transcriptome-based classifications have been reported recently, named PAM50 [37] and PCS [16]. The PAM50 classification (*n* = 3782) divided PCa patients into luminal A, luminal B, and basal subtypes, and the PCS classification (*n* > 4600) divided PCa patients into PCS1, PCS2, and PCS3 subtypes. A comparison of PCS and PAM50 showed that they were similar in molecular profiles and clinical outcomes, while the PCS system had greater separation regarding clinical outcomes [19]. Considering that PCS1 represents the most lethal and aggressive PCa subtype and the absence of therapeutic options for PCS1 in current clinical practice, we identified that a small-molecule drug, SR9009, selectively inhibited PCS1 tumors (>85% genes in PCS1 were inhibited) but had no impact on PCS2 and PCS3 tumors. Consequently, SR9009 could serve as a novel PCS1 inhibitor.

SR9009 is traditionally considered a putative ligand of REV-ERBs, and its therapeutic effects are mediated via REV-ERBs [10, 11, 13, 38, 39]. Nevertheless, recent studies suggested that the effects of SR9009 might also be independent of REV-ERBs. Dierickx and his colleagues found that the effect of SR9009 on cell proliferation and metabolism was not mediated by REV-ERBs [14]. Ishimaru et al. found that SR9009 had REV-ERB-independent effects in inhibiting mast cell activation [15]. In addition, Gao et al. identified that SR9009 prevented cellular senescence by activating NRF2 independently of REV-ERBs [40]. In line with the above findings, we demonstrated that SR9009 inhibited PCa cell viability and lethal PCS1 tumors through activation of LXRα instead of REV-ERBs. Evidence regarding the role of the REV-ERB-targeted gene BMAL1 in cancer cell biology is conflicting. Although several studies have demonstrated that BMAL1 promotes tumor progression [41, 42], many studies have revealed the tumor-suppressive role of BMAL1 [43**–**47]. We found in PCa that SR9009 upregulated BMAL1 expression instead of downregulating it, suggesting a potential synergetic role of SR9009 and BMAL1 in combating PCa. The specific mechanisms of SR9009-induced high BMAL1 expression remain unknown and should be investigated in further studies. In addition, previous research validated that SR9009 shows marked selectivity for LXRα over REV-ERBs (EC_50_ 6.3 μM for LXRα and > 50 μM for REV-ERBα) [28]. LXRα is a nuclear receptor that is considered to play a pivotal role in lipid metabolism [48]. Our GSEA also found that SR9009 upregulated the steroid hormone biosynthesis pathway (Fig. 2D). In addition, several studies revealed that LXRα activation by the synthetic LXR agonists T0901317 and 22(R)-hydroxycholesterol inhibited PCa cell proliferation [49, 50]. We provide new evidence that SR9009 cannot be applied solely as a putative REV-ERB agonist but to be served as a novel LXRα agonist.

FOXM1 is a crucial transcription factor in cancer and maintains cancer hallmarks by regulating its target gene expression [51]. From TCGA databases, we found that FOXM1 is aberrantly overexpressed in most cancer types and that its high expression was associated with worse survival outcomes. Targeting the FOXM1 pathway is an intriguing strategy for combating cancer. FOXM1 was also reported to be the master driver of lethal PCS1 tumors [20]. We showed that REV-ERBs did not regulate FOXM1 or its target gene expression. Instead, LXRα silencing or pharmacological activation by SR9009 regulated the FOXM1 transcription network. Although the direct regulation of FOXM1 by LXRα was validated in hepatocellular carcinoma and macrophages [29, 52], our study validated this regulation in PCa and provided a novel therapeutic strategy for targeting the FOXM1 pathway and PCS1 subtype.

Our study demonstrated that SR9009 can serve as an effective treatment choice for highly aggressive and lethal PCS1 tumors by mediating the LXRα/FOXM1 pathway independently of REV-ERBs. Nevertheless, the other functions of SR9009 in PCa also warrant further exploration, such as the impact of SR9009 on metabolism, ferroptosis and cellular senescence. Although we showed that SR9009 could serve as both a FOXM1 pathway and PCS1 subtype inhibitor, the application of PCS classification in the clinic is still underway, and prospective clinical trials are warranted to validate the efficacy and safety of SR9009 in mCRPC treatment as a monotherapy or combination therapy.

## Conclusions

In summary, our study demonstrated for the first time that SR9009 could selectively kill PCa cells in a dose-dependent manner but had no impact on normal prostate cells. SR9009 could inhibit the most aggressive and lethal PCS1 tumors through FOXM1 pathway blockade. The effects of SR9009 on PCa cell proliferation are mediated by LXRα activation instead of REV-ERBs. Our study provides new insights into the function of SR9009 and into the discovery of potential useful drugs for PCa therapy.

## Supplementary information

Fig. S1

Fig. S2

Fig. S3

Fig. S4

Fig. S5

Fig. S6

Table S1

Table S2

Table S3

Checklist

Full and uncropped WB

## Data Availability

The data and materials used in the current study are available from the corresponding author upon request.
