# Supplementary figures and images for: SR9009 inhibits lethal prostate cancer subtype 1 by regulating the LXRα/FOXM1 pathway independently of REV-ERBs
Source: Cell Death Dis. 2022 Nov 10;13(11):949. doi: 10.1038/s41419-022-05392-6 (PMC9649669; doi:10.1038/s41419-022-05392-6)

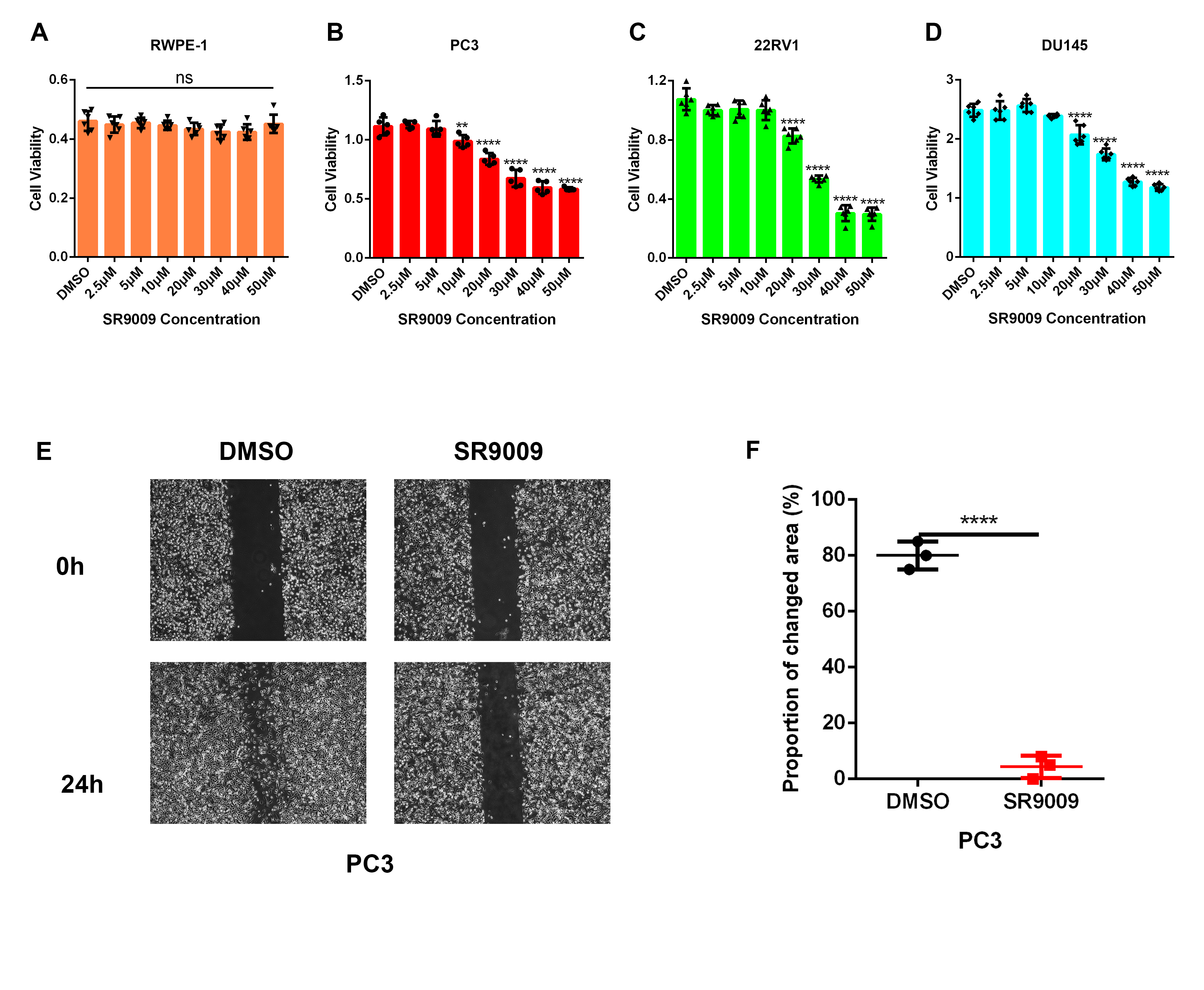

Supplement: Supplementary file 1 — Fig. S1 [file 41419_2022_5392_MOESM1_ESM.tif]

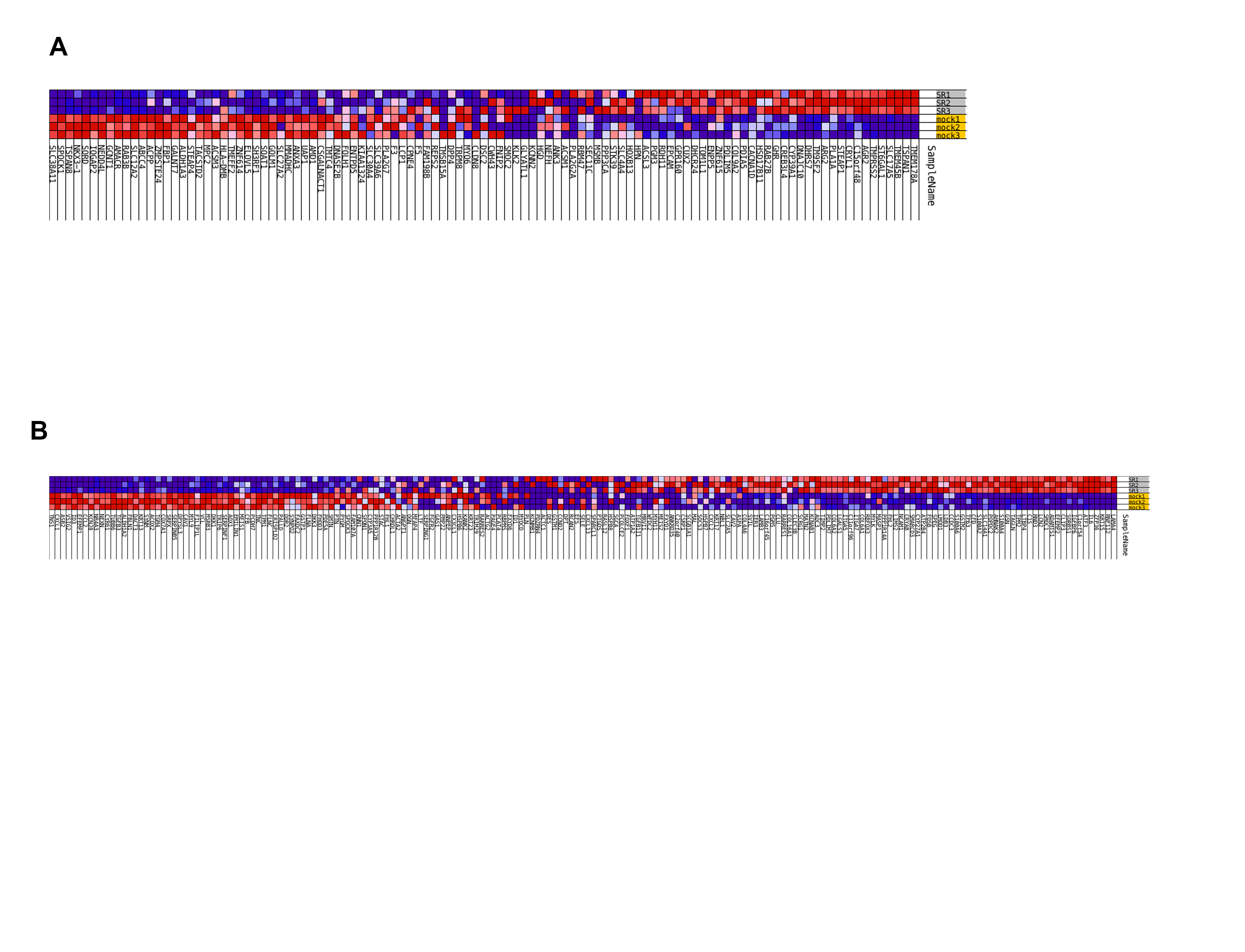

Supplement: Supplementary file 2 — Fig. S2 [file 41419_2022_5392_MOESM2_ESM.tif]

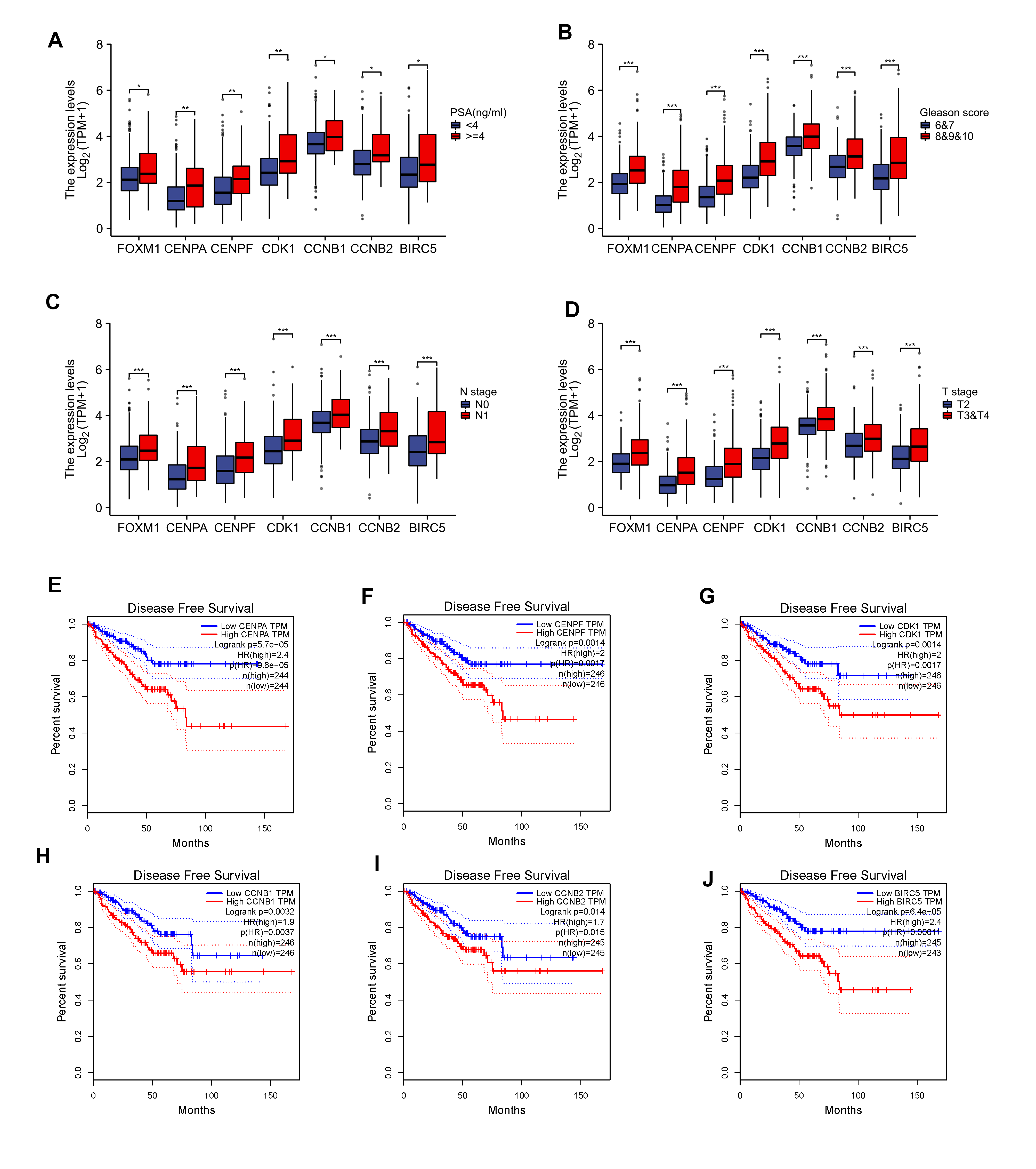

Supplement: Supplementary file 3 — Fig. S3 [file 41419_2022_5392_MOESM3_ESM.tif]

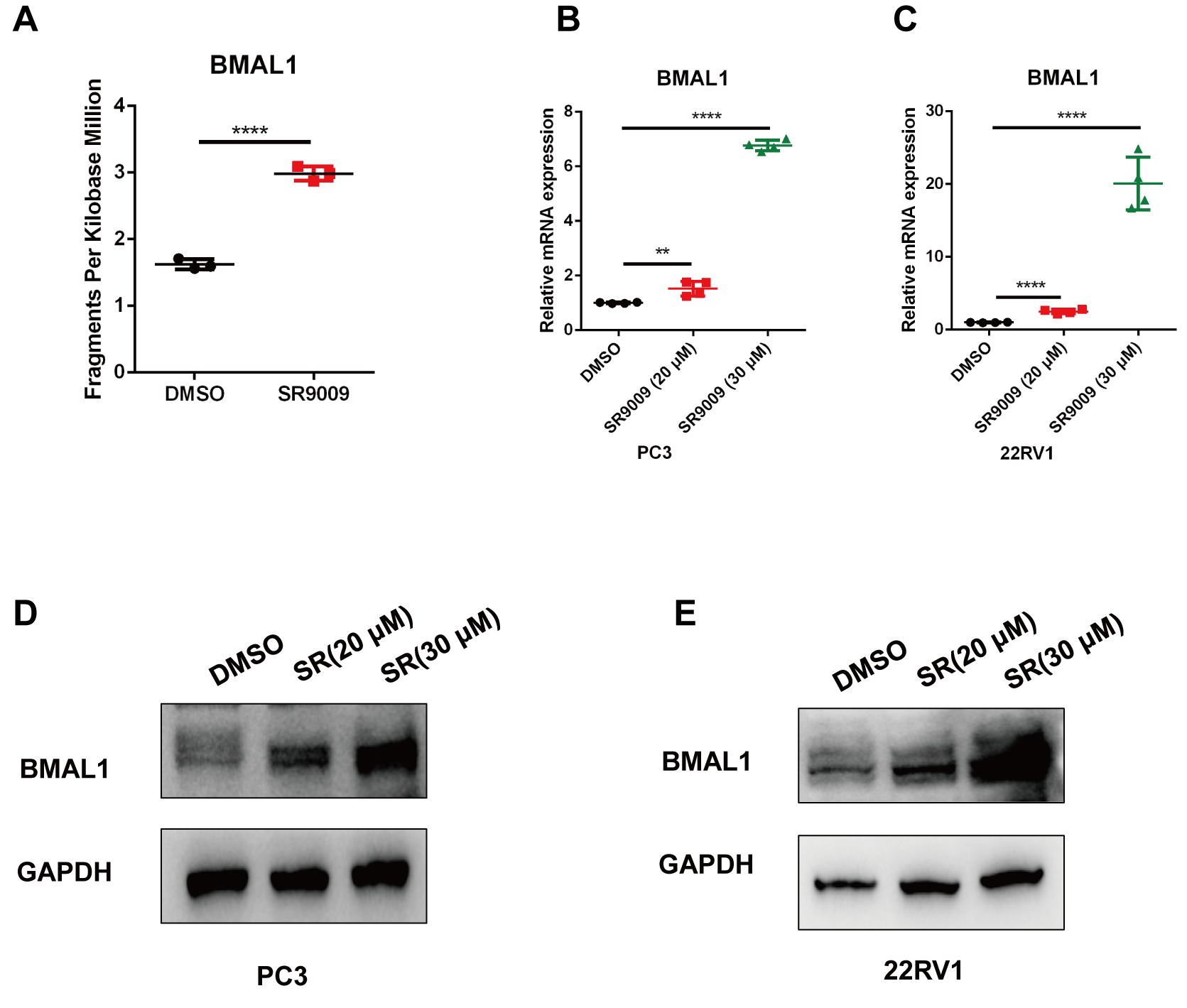

Supplement: Supplementary file 4 — Fig. S4 [file 41419_2022_5392_MOESM4_ESM.tif]

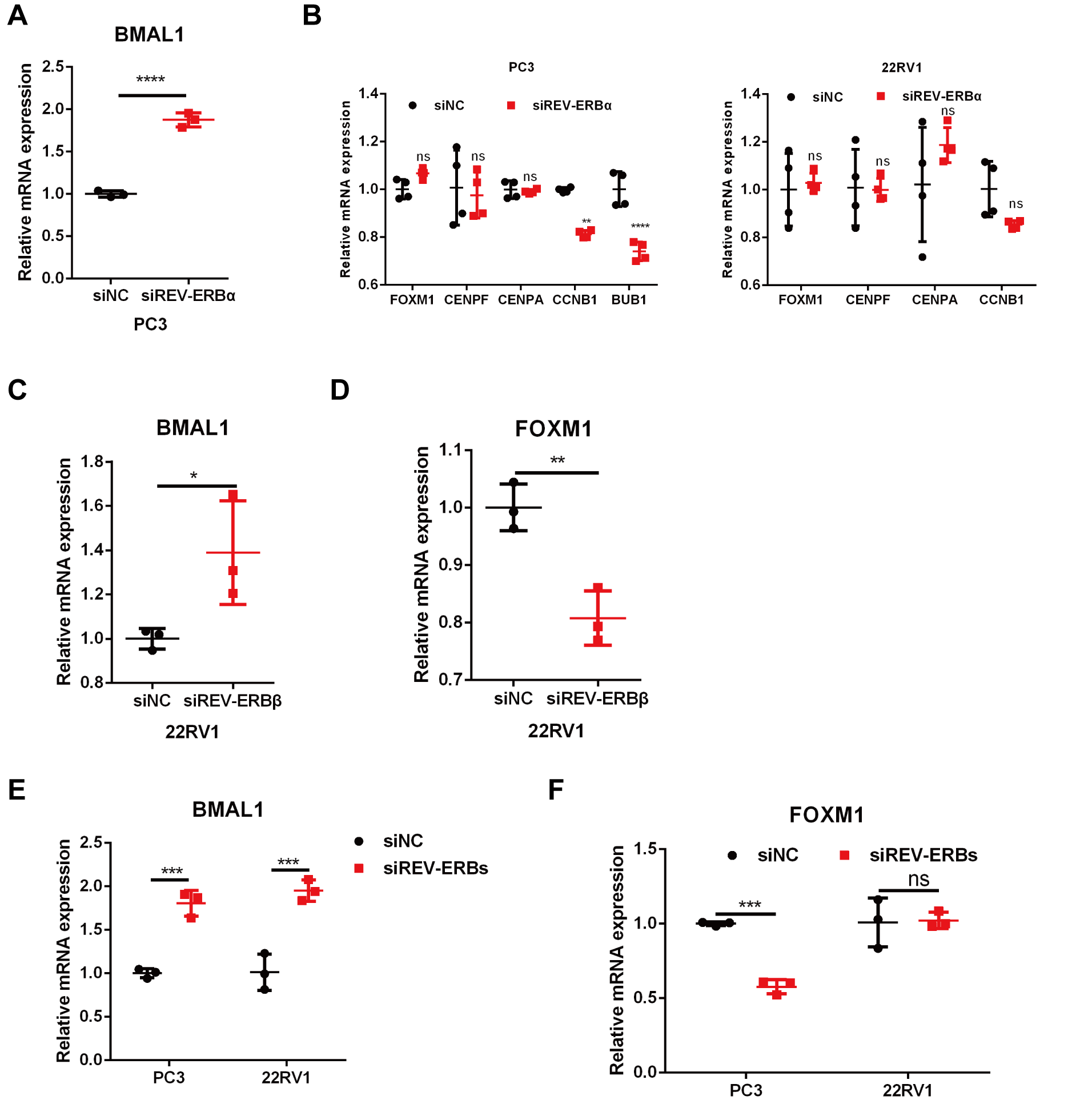

Supplement: Supplementary file 5 — Fig. S5 [file 41419_2022_5392_MOESM5_ESM.tif]

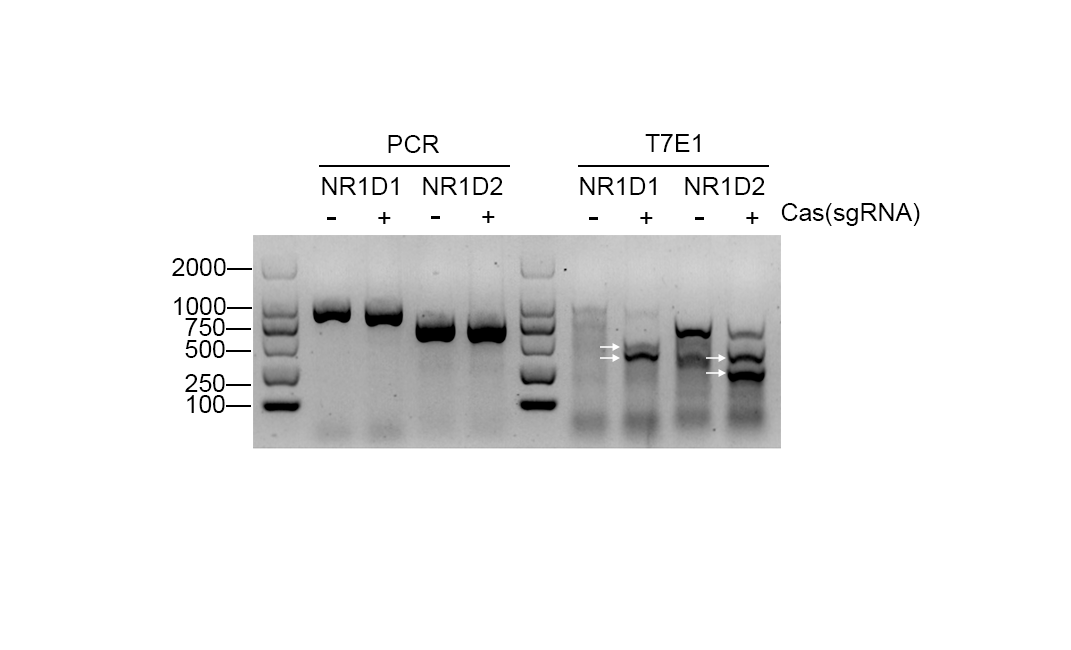

Supplement: Supplementary file 6 — Fig. S6 [file 41419_2022_5392_MOESM6_ESM.png]
