# Supplementary material for: SR9009 inhibits lethal prostate cancer subtype 1 by regulating the LXRα/FOXM1 pathway independently of REV-ERBs
Source: Cell Death Dis. 2022 Nov 10;13(11):949. doi: 10.1038/s41419-022-05392-6 (PMC9649669; doi:10.1038/s41419-022-05392-6)
Supplement: Supplementary file 7 — Table S1 [file 41419_2022_5392_MOESM7_ESM.docx]

**Table S1** siRNA sequences

| **Targets** | **No.** | **sequence** |
| --- | --- | --- |
| NR1D1 | si#1 | CATGTCCTATGAACATGTA |
|  | si#2 | GCAACTCAAAGAATGTTCT |
|  | si#3 | GTGCGCTTTGCTTCGTTGT |
| NR1D2 | si#1 | GAGAACGGATTCCCAAGAA |
|  | si#2 | GATCTTCGATCTTTAAACA |
|  | si#3 | TTCGGAGAAGTATTCAACA |
| NR1H3 | si#1 | GAGTGTCGGCTTCGCAAAT |
|  | si#2 | TCATCAAGGGAGCGCACTA |
|  | si#3 | TCAGTTATAACCGGGAAGA |
| FOXM1 | si#1 | CCAACAATGCTAATATTCA |
|  | si#2 | GCAGAAACGACCGAATCCA |
|  | si#3 | AGTGCCAACCGCTACTTGA |
