# Supplementary material for: SR9009 inhibits lethal prostate cancer subtype 1 by regulating the LXRα/FOXM1 pathway independently of REV-ERBs
Source: Cell Death Dis. 2022 Nov 10;13(11):949. doi: 10.1038/s41419-022-05392-6 (PMC9649669; doi:10.1038/s41419-022-05392-6)
Supplement: Supplementary file 8 — Table S2 [file 41419_2022_5392_MOESM8_ESM.docx]

**Table S2** Sequences of the sgRNAs

| **Gene name** | **Forward Primer** | **Reverse Primer** |
| --- | --- | --- |
| NR1D1 | CACCGGTGGCGTCATCACCTACAT | AAACATGTAGGTGATGACGCCACC |
| NR1D2 | CACCGCTTCAATAGCTGTTCGGTT | AAACAACCGAACAGCTATTGAAGC |
