# Supplementary material for: SR9009 inhibits lethal prostate cancer subtype 1 by regulating the LXRα/FOXM1 pathway independently of REV-ERBs
Source: Cell Death Dis. 2022 Nov 10;13(11):949. doi: 10.1038/s41419-022-05392-6 (PMC9649669; doi:10.1038/s41419-022-05392-6)
Supplement: Supplementary file 9 — Table S3 [file 41419_2022_5392_MOESM9_ESM.docx]

**Table S3** Sense and antisense of the primers

| **Gene name** | **Forward Primer** | **Reverse Primer** |
| --- | --- | --- |
| NR1D1 | TGGACTCCAACAACAACACAG | GATGGTGGGAAGTAGGTGGG |
| NR1D2 | TTTAGTGGCATGGTTCTACTGTG | AGCCTTCGCAAGCATGAACT |
| BMAL1(ARNTL) | AAGGGAAGCTCACAGTCAGAT | GGACATTGCGTTGCATGTTGG |
| FOXM1 | CGTCGGCCACTGATTCTCAAA | GGCAGGGGATCTCTTAGGTTC |
| CENPF | CTCTCCCGTCAACAGCGTTC | GTTGTGCATATTCTTGGCTTGC |
| PLK1 | CACCAGCACGTCGTAGGATTC | CCGTAGGTAGTATCGGGCCTC |
| CCNB1 | AATAAGGCGAAGATCAACATGGC | TTTGTTACCAATGTCCCCAAGAG |
| AURKB | CAGTGGGACACCCGACATC | GTACACGTTTCCAAACTTGCC |
| AURKA | GGAATATGCACCACTTGGAACA | TAAGACAGGGCATTTGCCAAT |
| BUB1 | ACAATCAACGGAGAAAGCATGA | CTCCACCACCTGATGCAACT |
| CENPA | TTCCTCCCATCAACACAGTCG | CACACCACGAGTGAATTTAACAC |
| CCNA2 | CGCTGGCGGTACTGAAGTC | GAGGAACGGTGACATGCTCAT |
| CCNE2 | TCAAGACGAAGTAGCCGTTTAC | TGACATCCTGGGTAGTTTTCCTC |
| SKP2 | ATGCCCCAATCTTGTCCATCT | CACCGACTGAGTGATAGGTGT |
| RRM2 | CACGGAGCCGAAAACTAAAGC | TCTGCCTTCTTATACATCTGCCA |
| NR1H3 | CCTTCAGAACCCACAGAGATCC | ACGCTGCATAGCTCGTTCC |
| CDK1 | GGATGTGCTTATGCAGGATTCC | CATGTACTGACCAGGAGGGATAG |
| BIRC5 | AGGACCACCGCATCTCTACAT | AAGTCTGGCTCGTTCTCAGTG |
| CCNB2 | AATAAGGCGAAGATCAACATGGC | TTTGTTACCAATGTCCCCAAGAG |
| ACTB | CATGTACGTTGCTATCCAGGC | CTCCTTAATGTCACGCACGAT |
