# Supplementary material for: SR9009 inhibits lethal prostate cancer subtype 1 by regulating the LXRα/FOXM1 pathway independently of REV-ERBs
Source: Cell Death Dis. 2022 Nov 10;13(11):949. doi: 10.1038/s41419-022-05392-6 (PMC9649669; doi:10.1038/s41419-022-05392-6)

Figure 2K

22rv1-foxm1


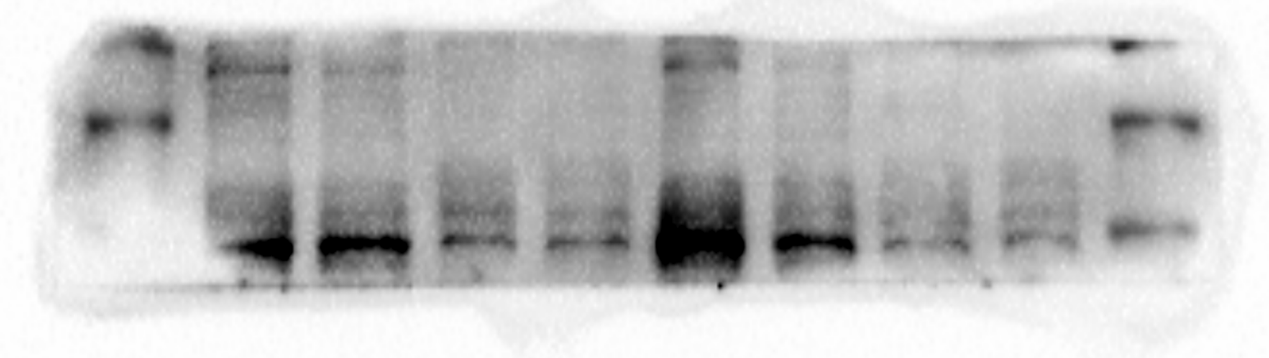


PC3-foxm1


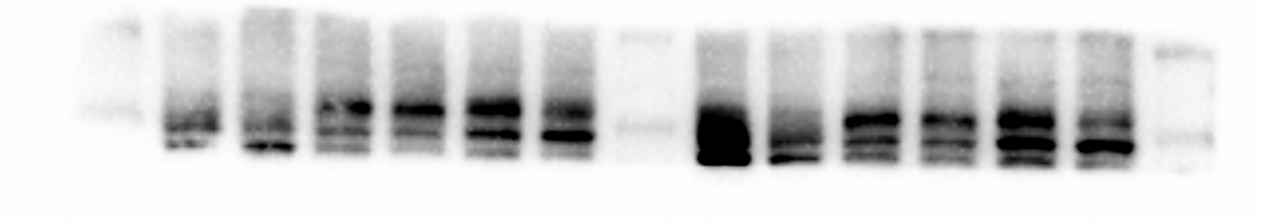


RRM2


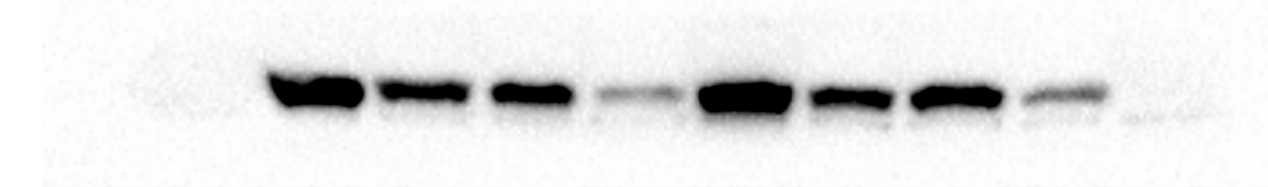


GAPDH


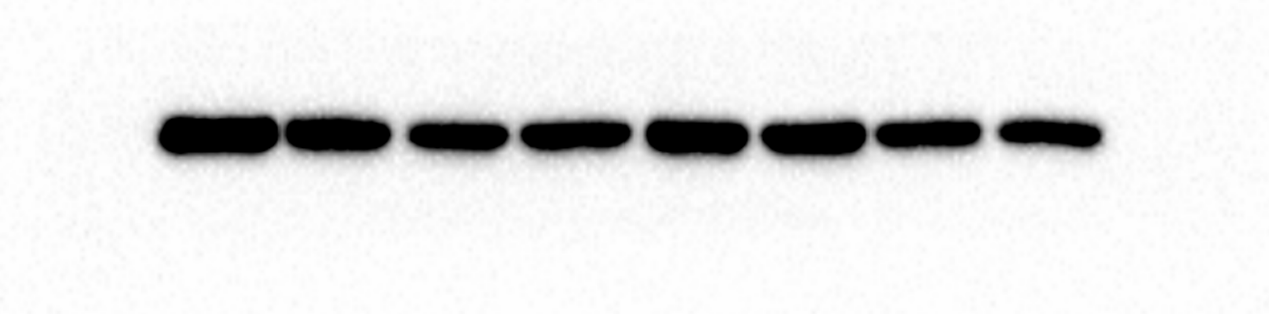


Figure 3

22RV1-ACTIN


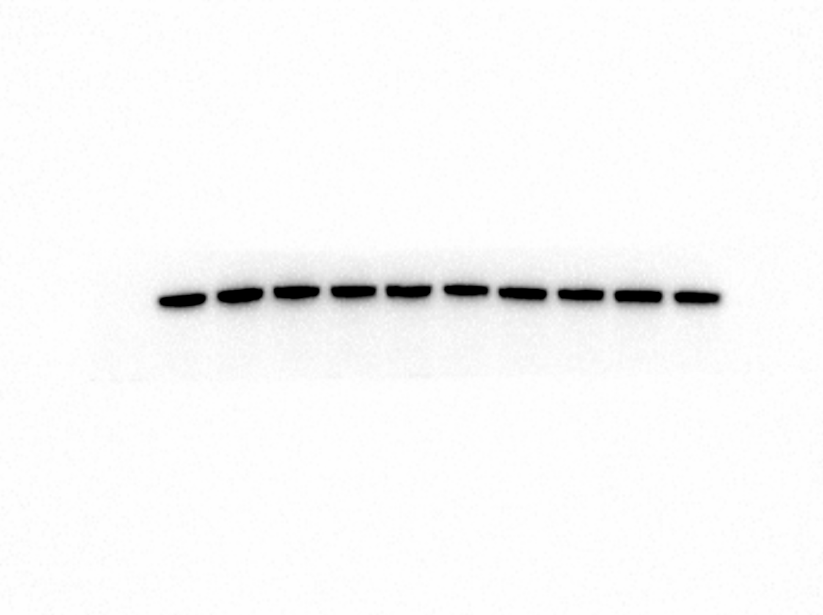


22RV1-FOXM1


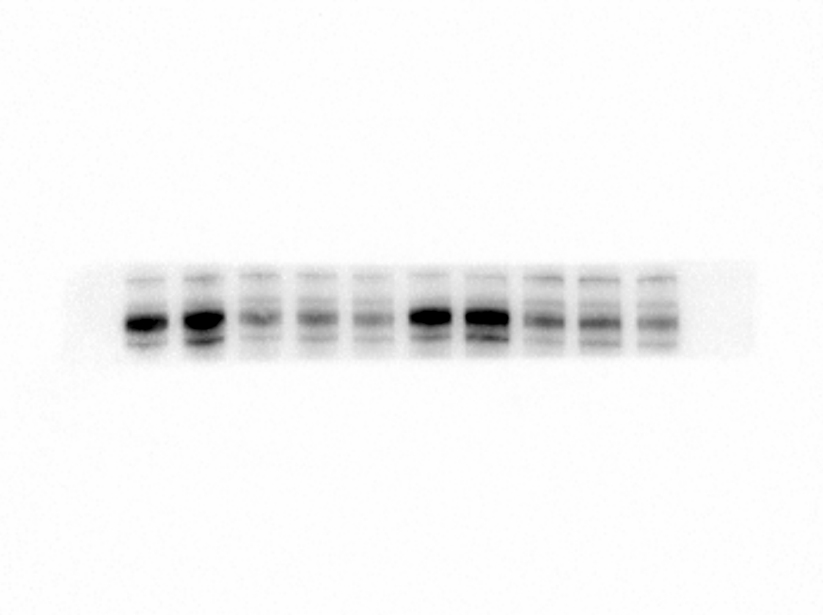


FOXM1-OE


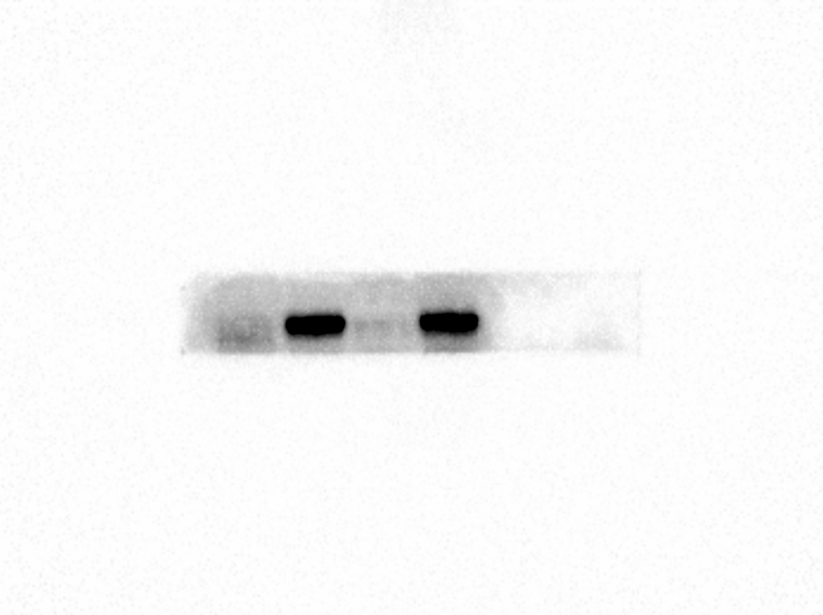


GAPDH_FOXM1OE


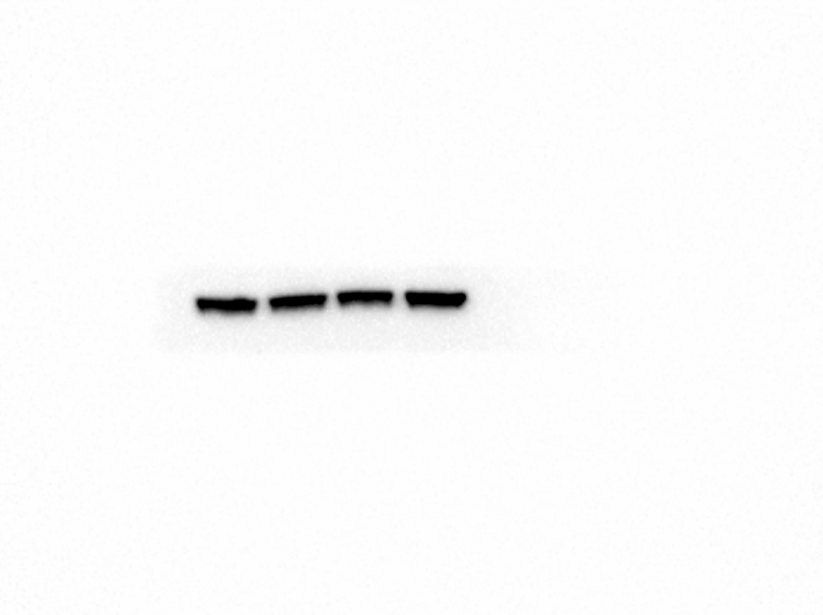


PC3-FOXM1


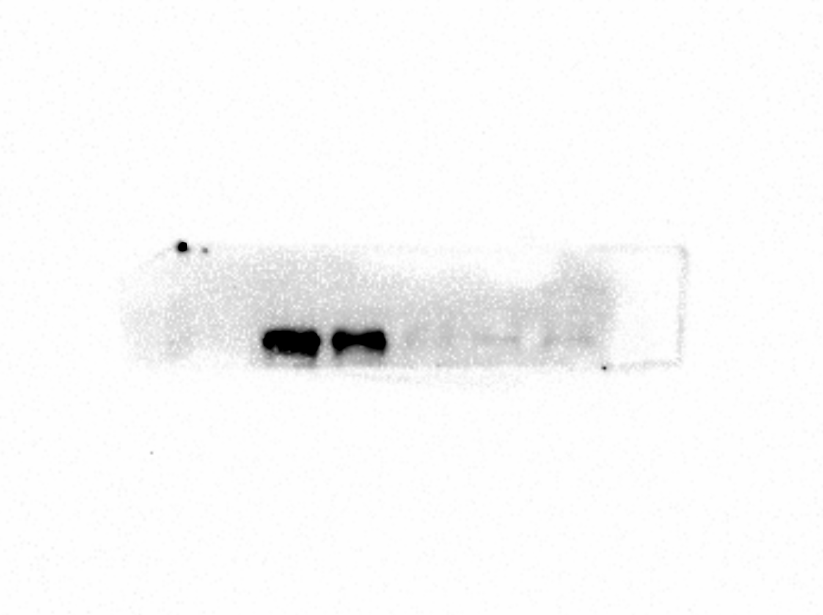


PC3_GAPDH


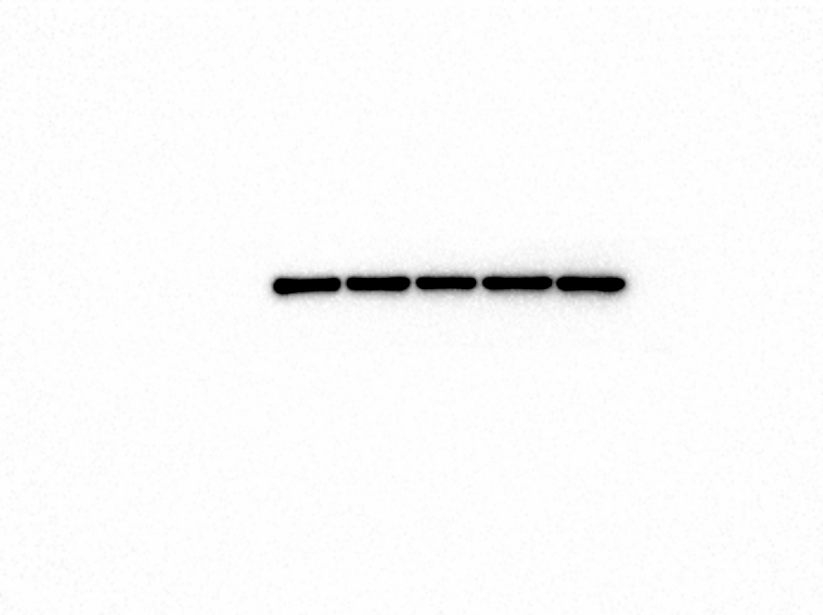


Figure 4F

CCNB1


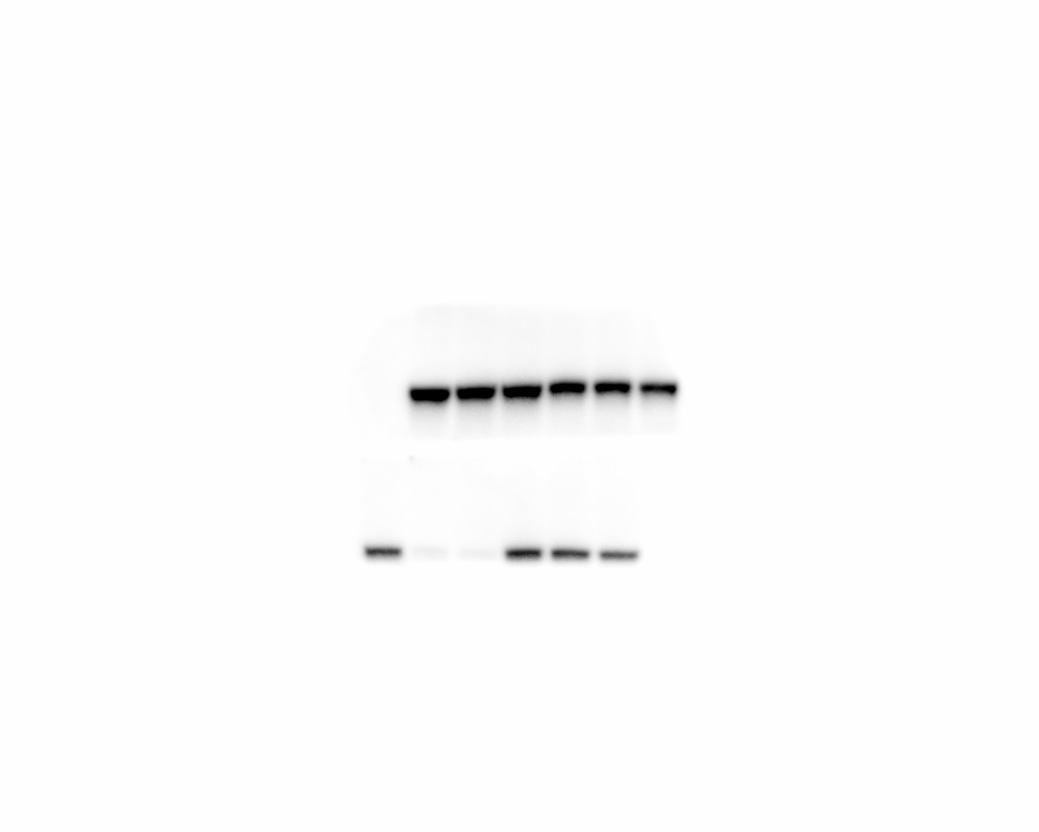


CCNB2


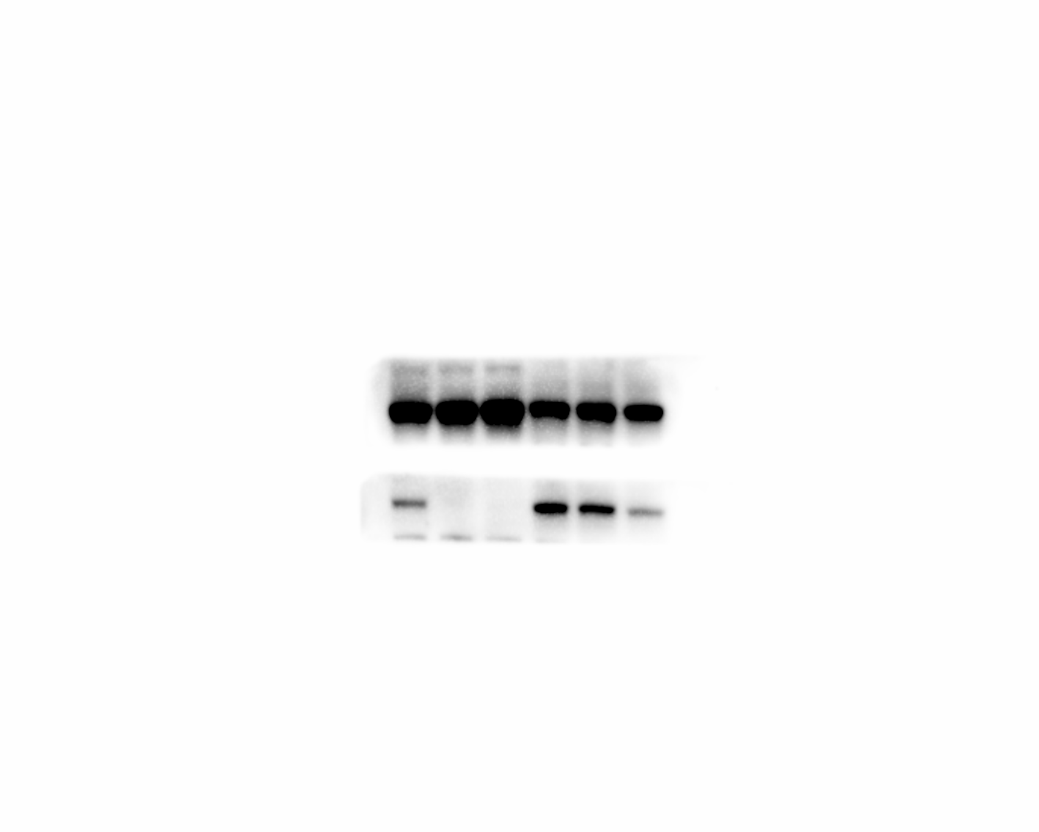


CDK1


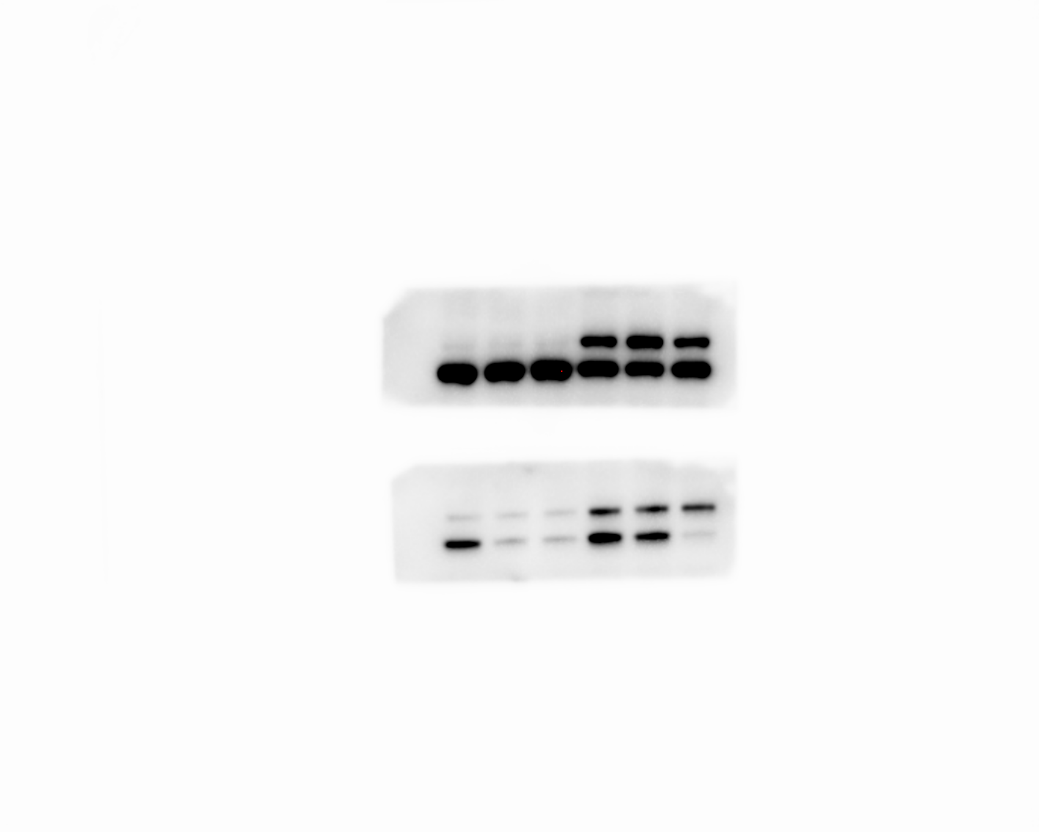


CENPA


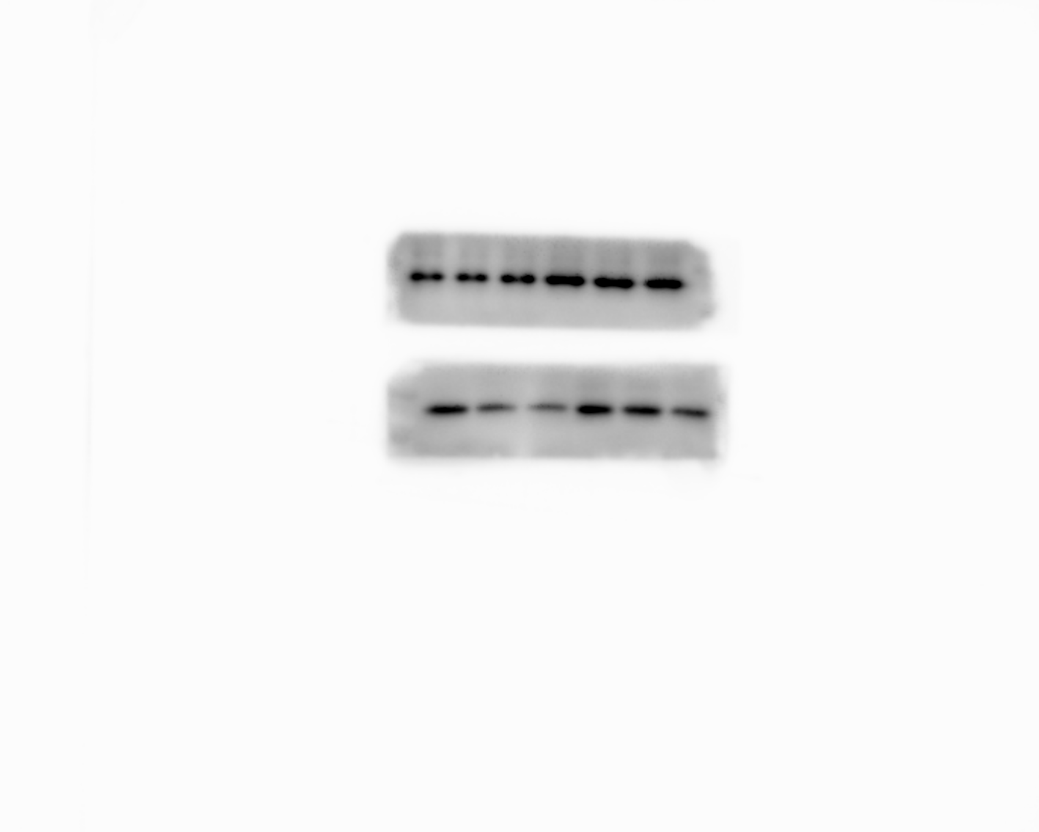


CENPF


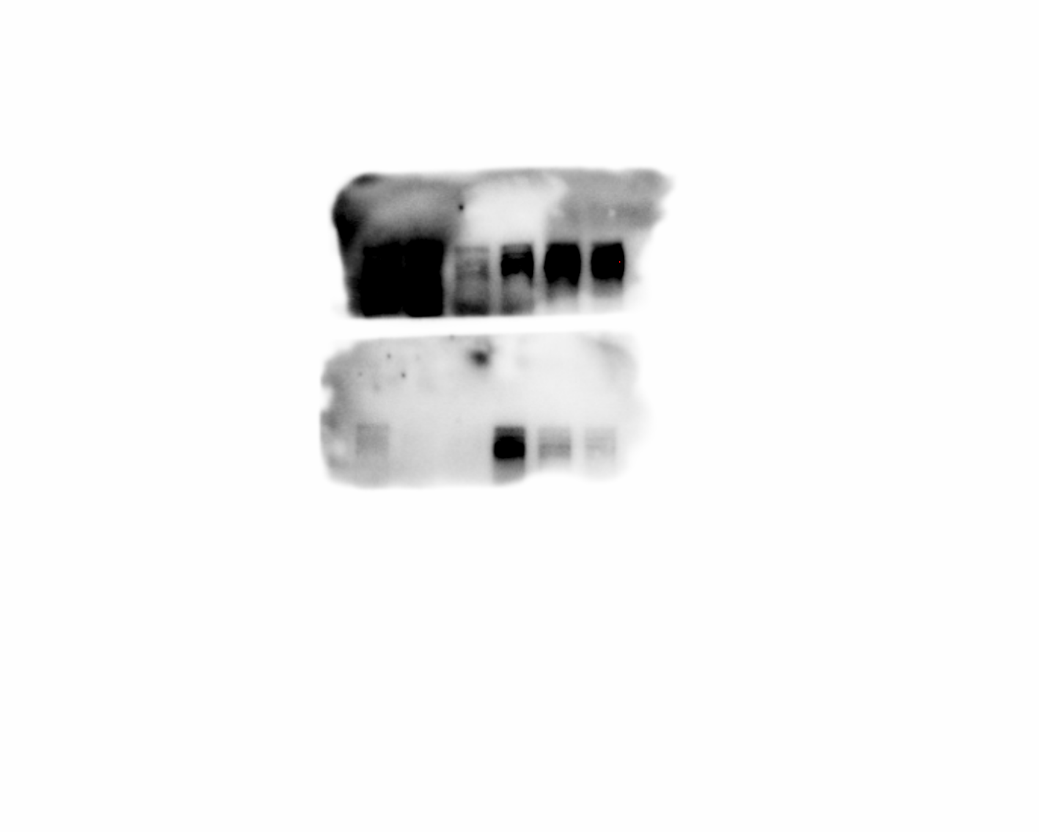


FOXM1


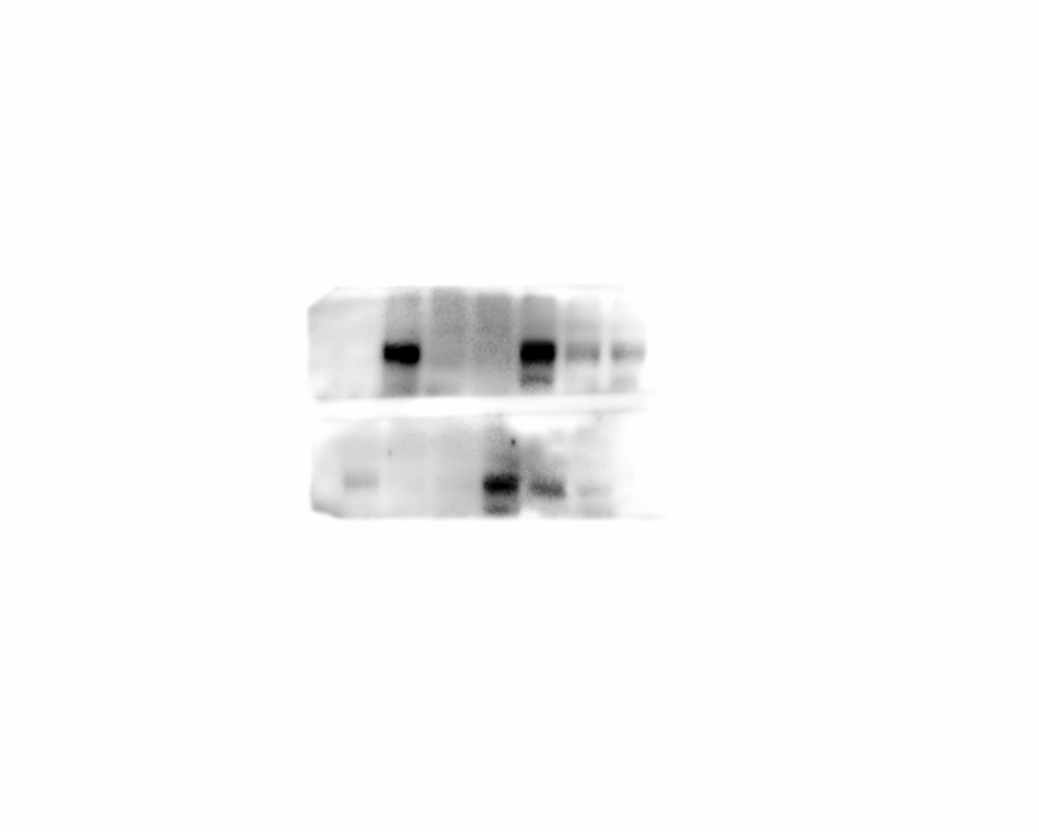


GAPDH


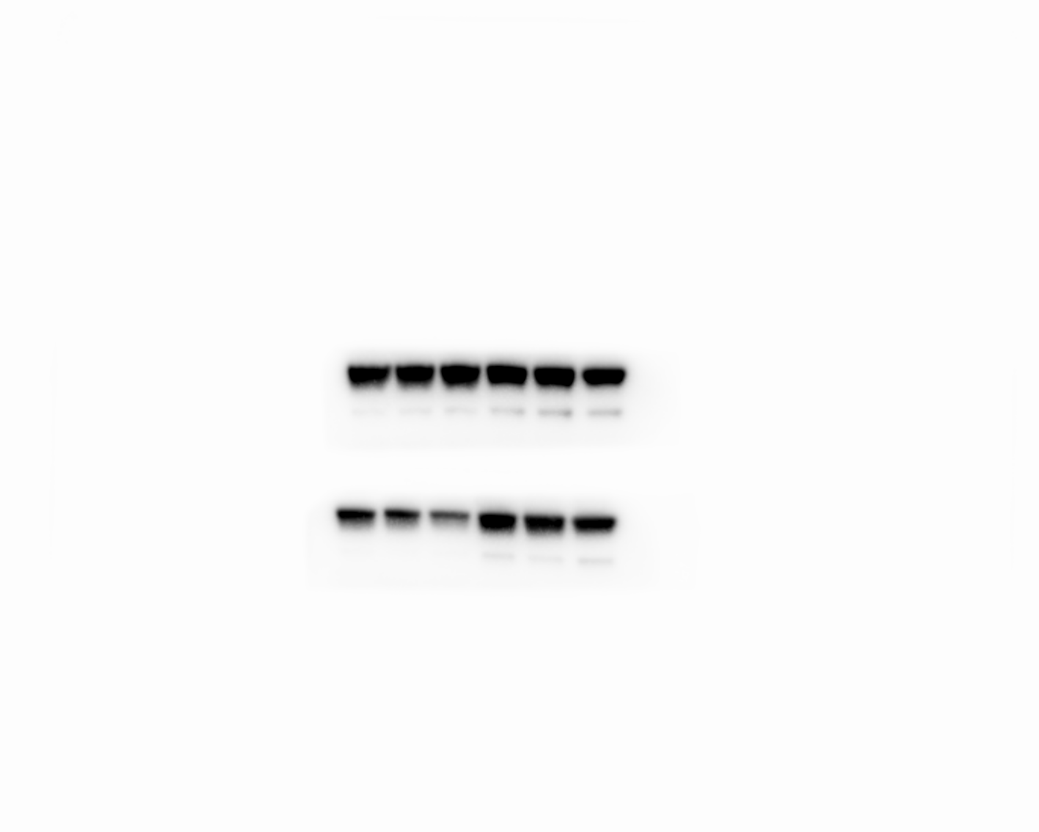


Suivivin


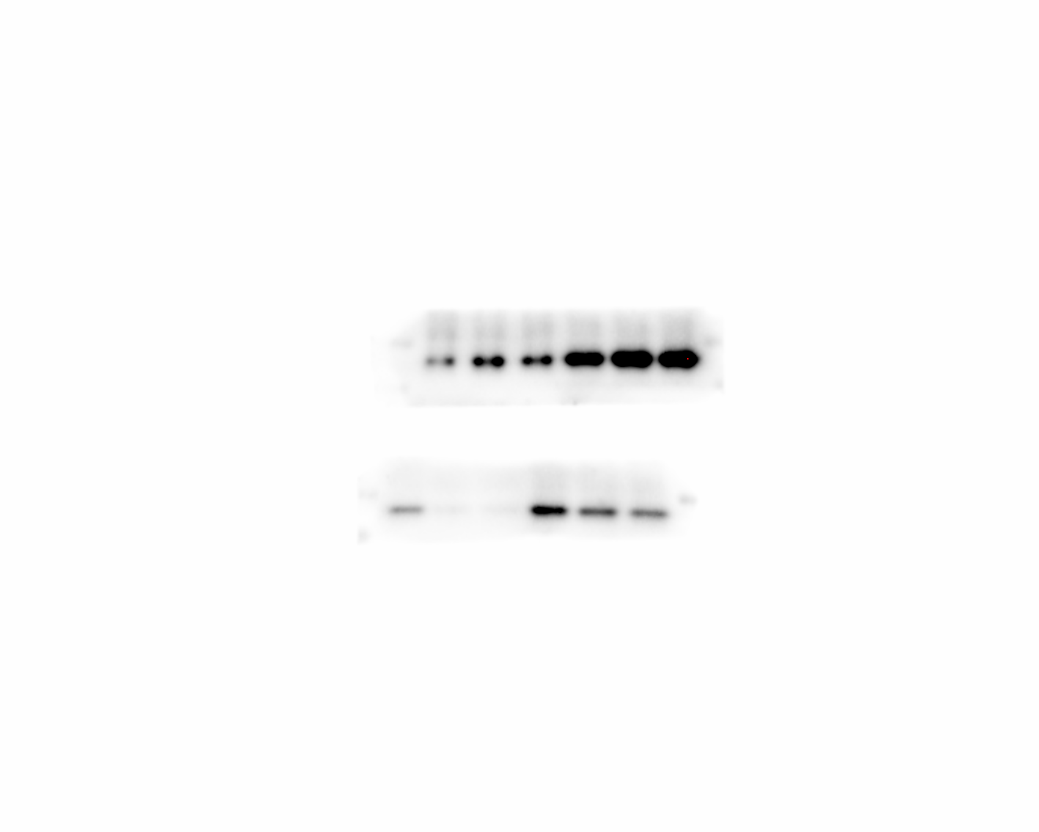


Figure 4H

22RV1-CDK1


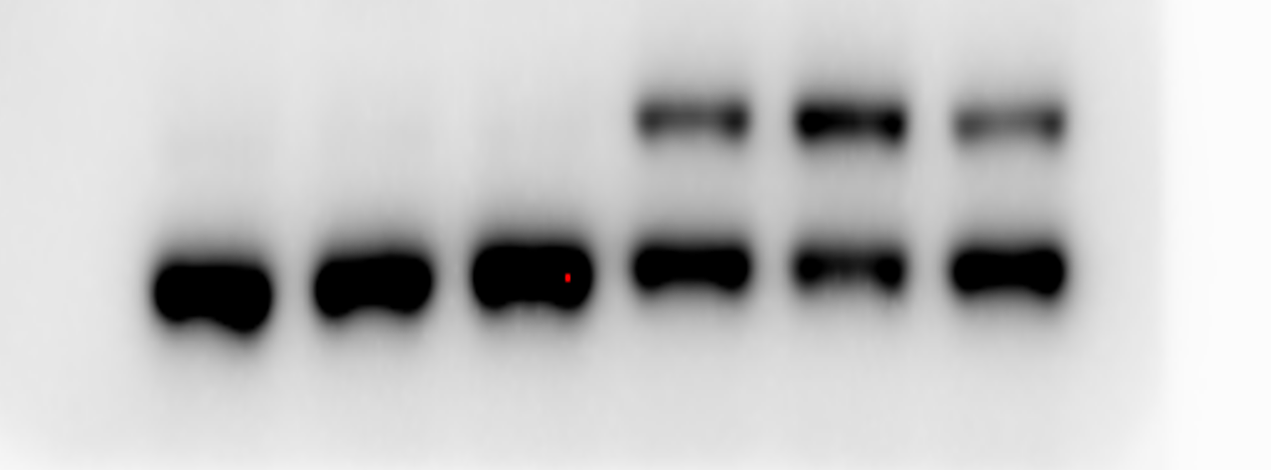


CCNB1


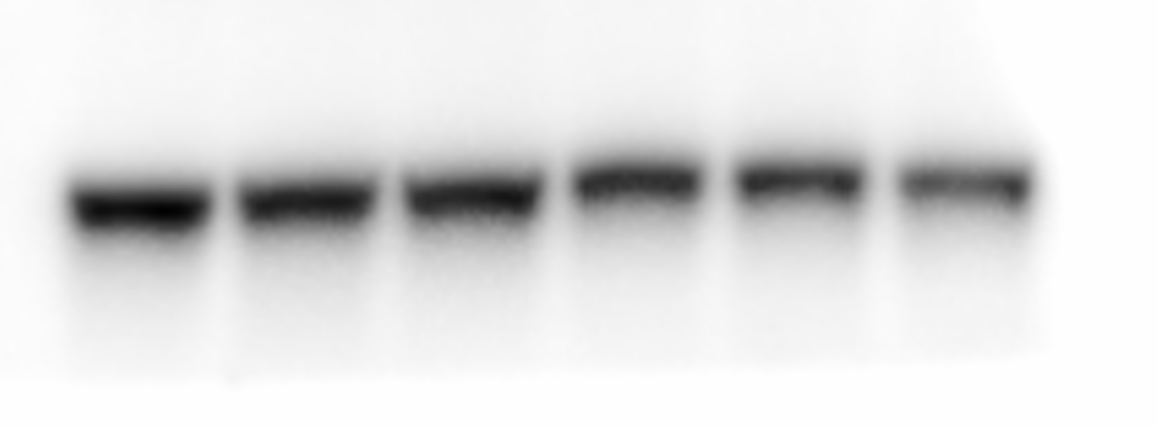


FOXM1


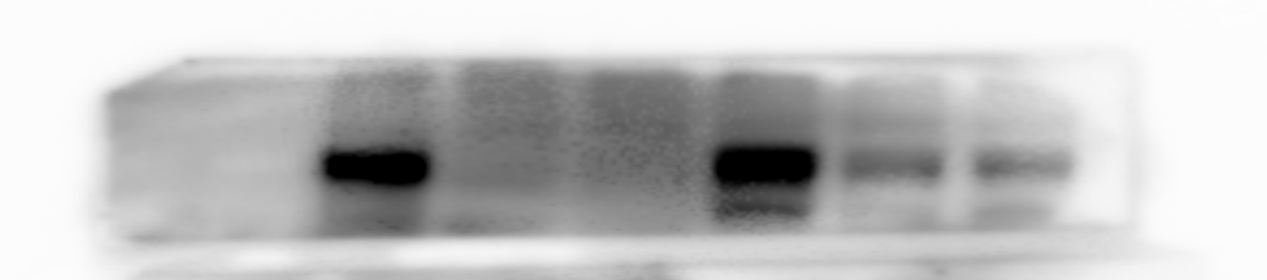


GAPDH


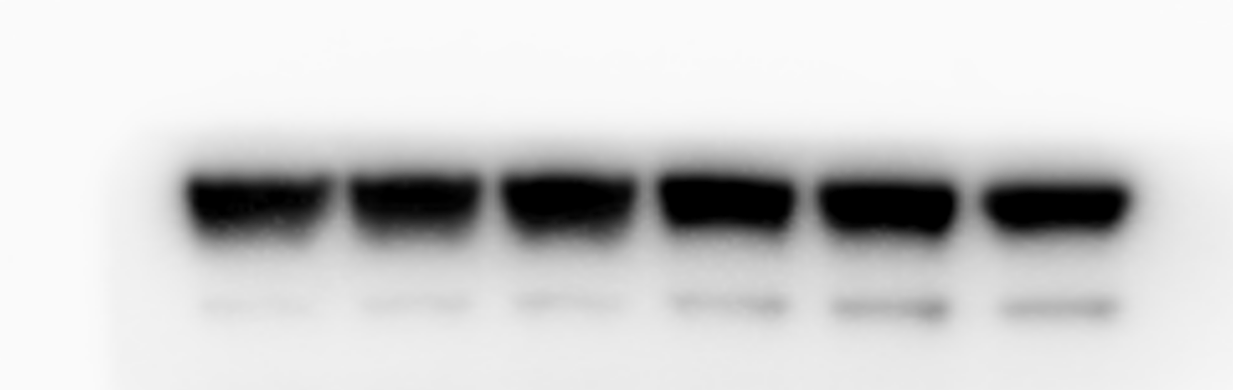


PC3-CDK1


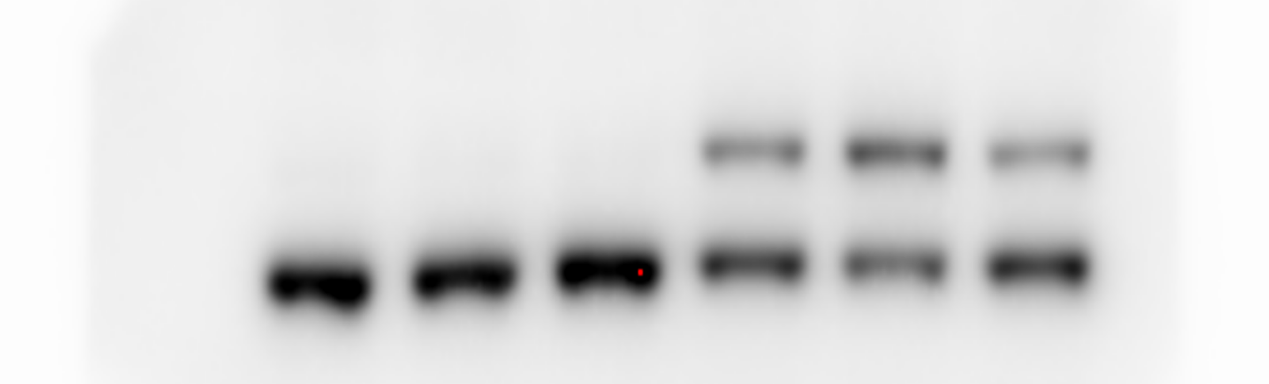


Figure 4I

CDK1


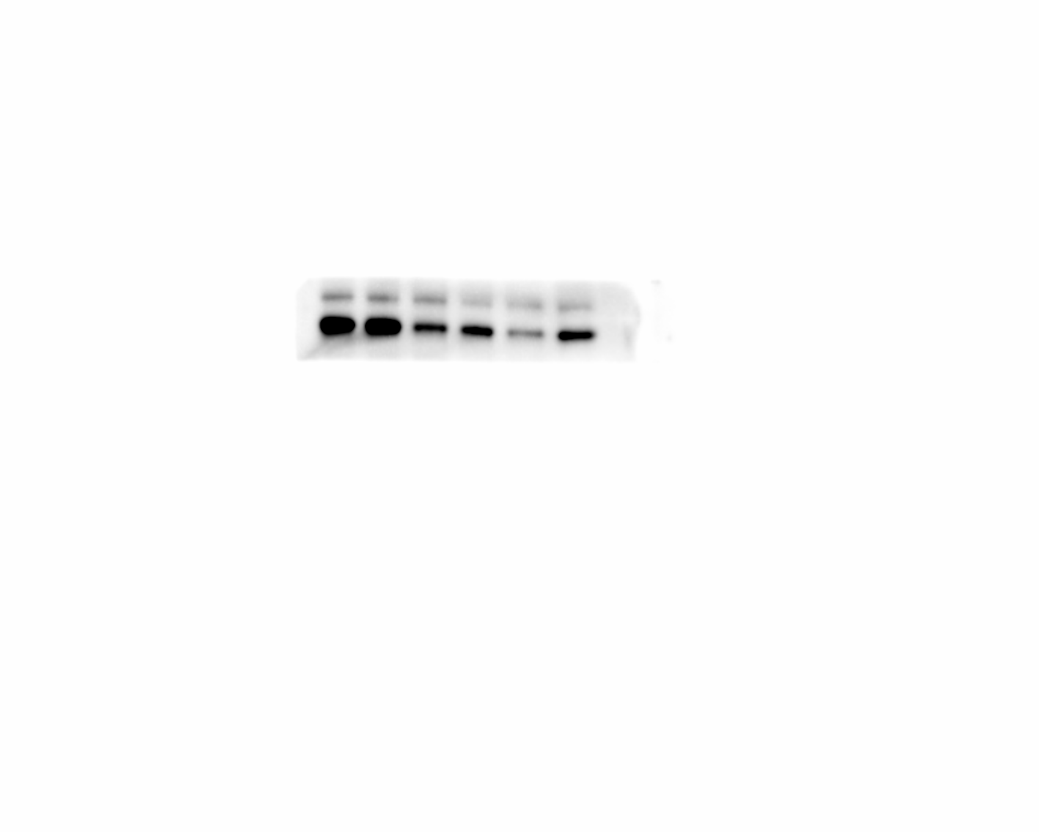


CENPF


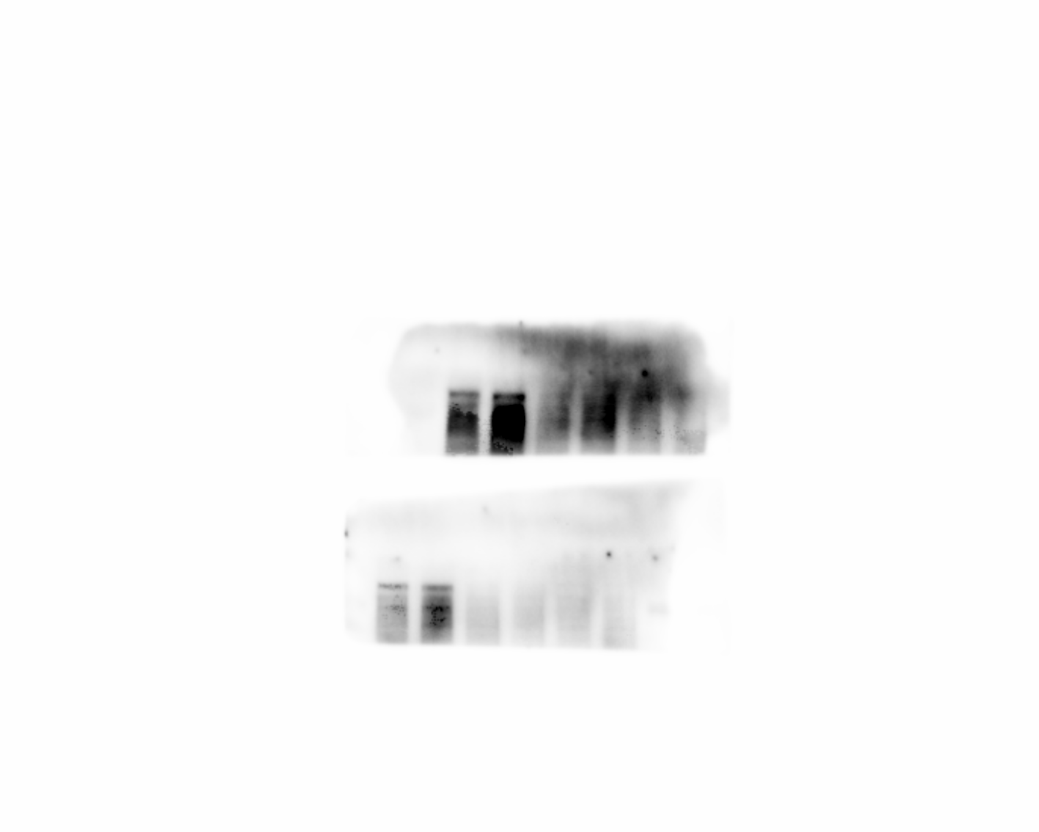


FOXM1


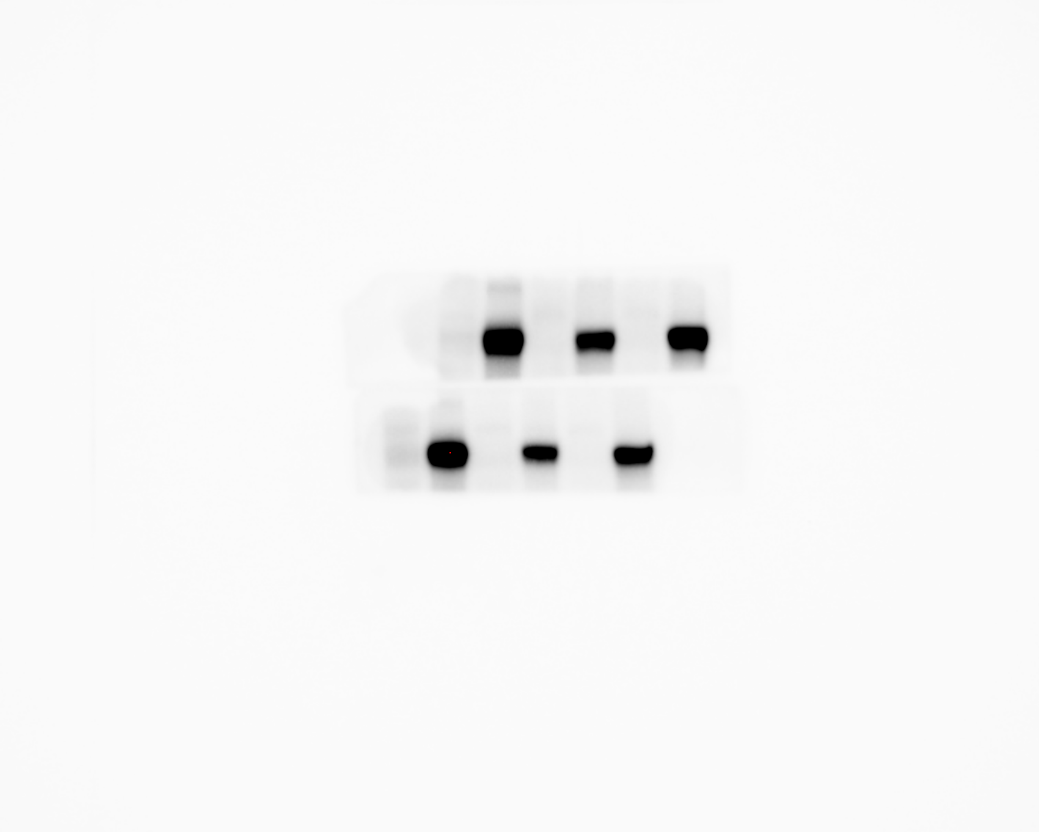


SURVIVIN


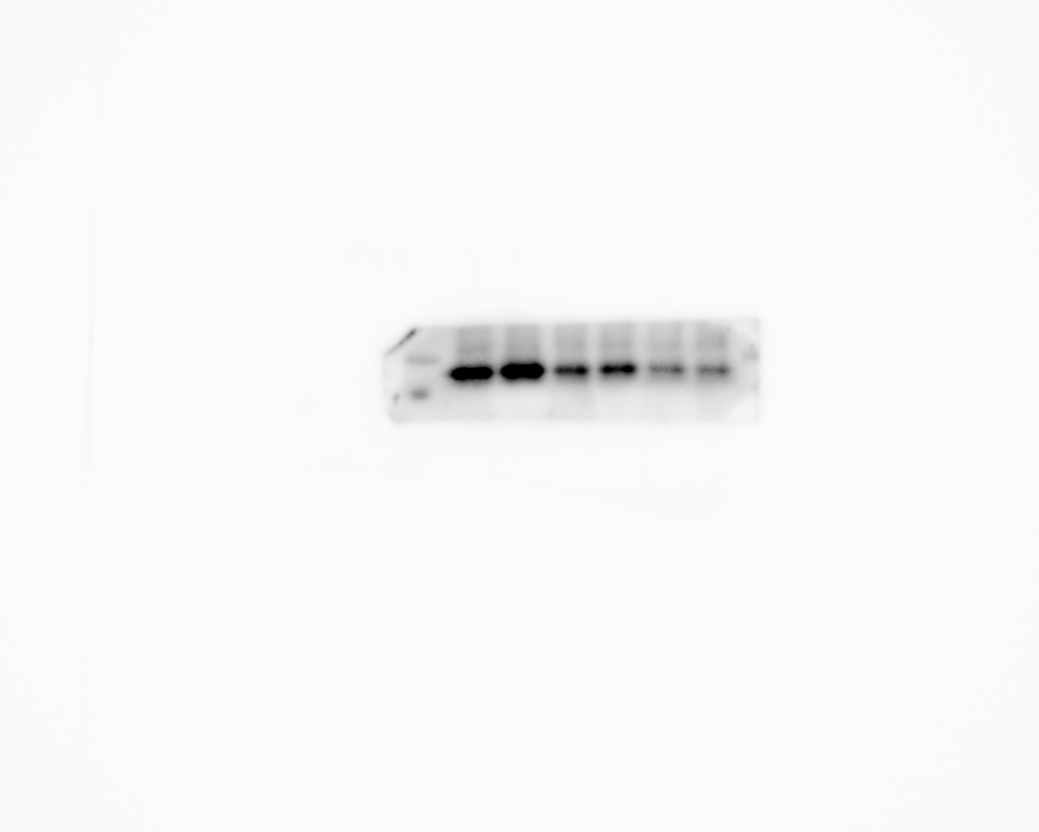


GAPDH


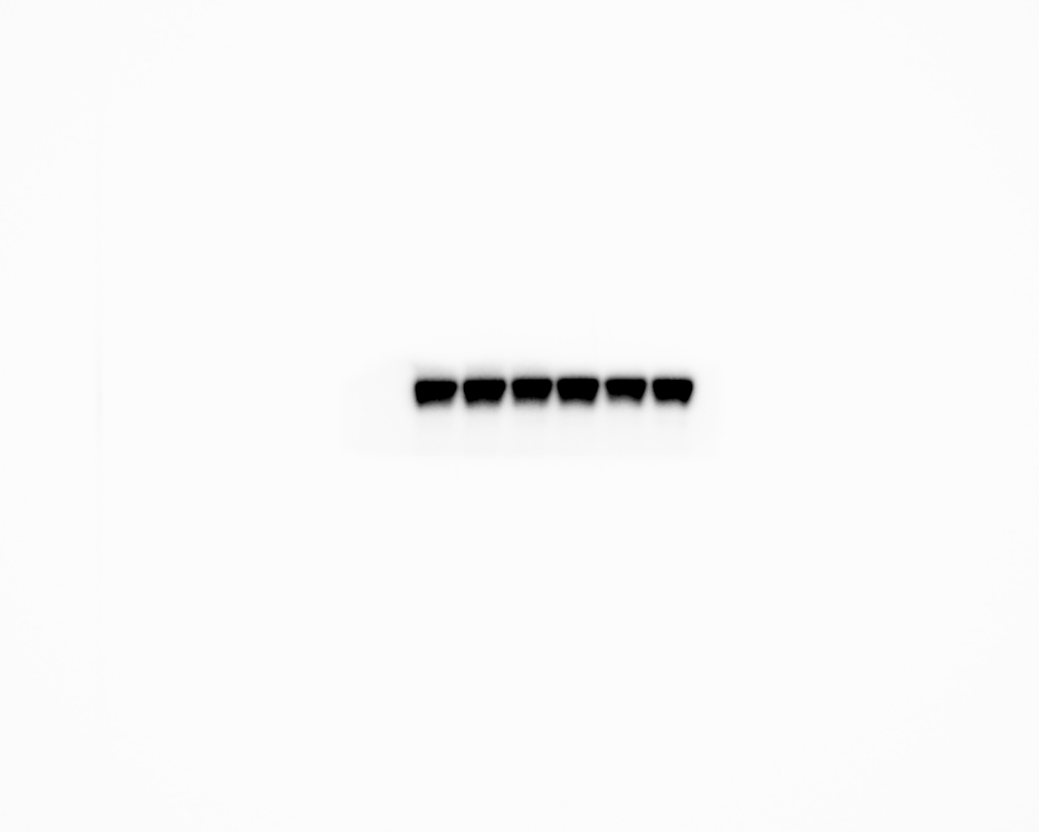


Figure 5

C

22rv1-gapdh


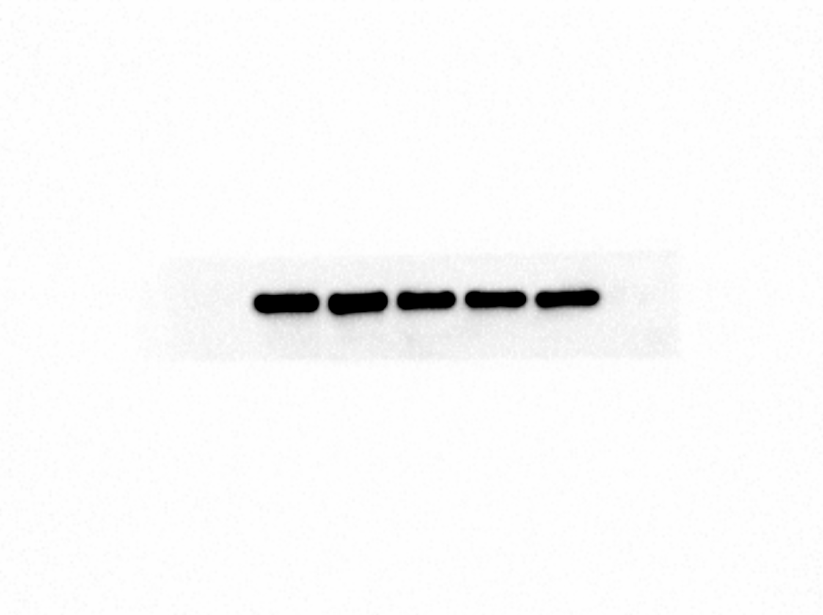


22rv1-nr1d1


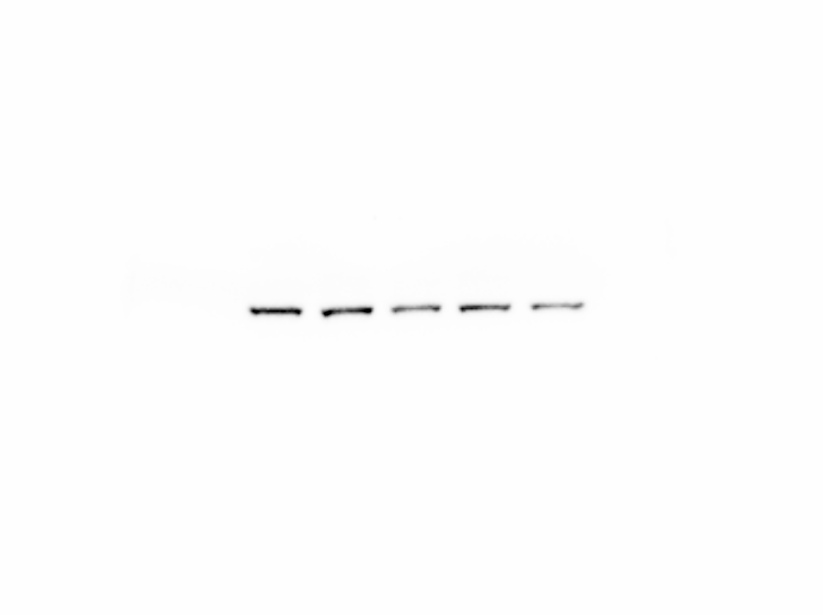


Pc3-gapdh


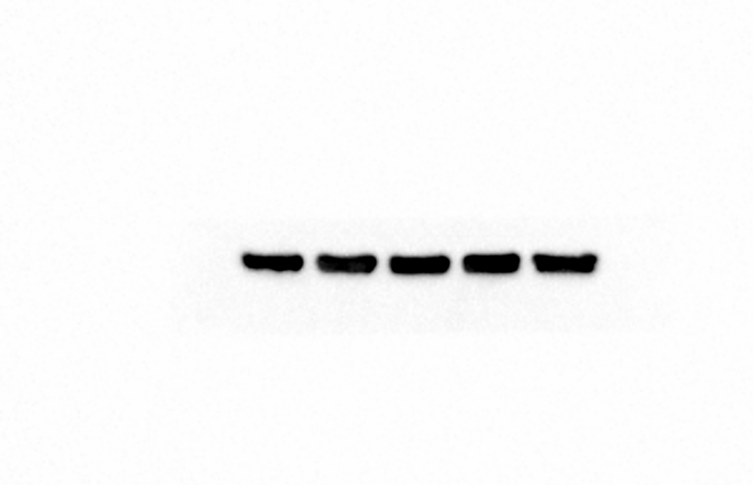


Pc3-nr1d1


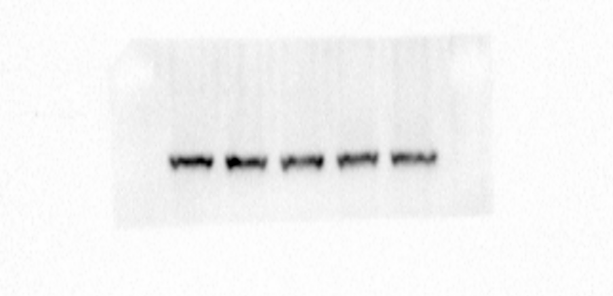


Figure 5G

22RV1-GAPDH


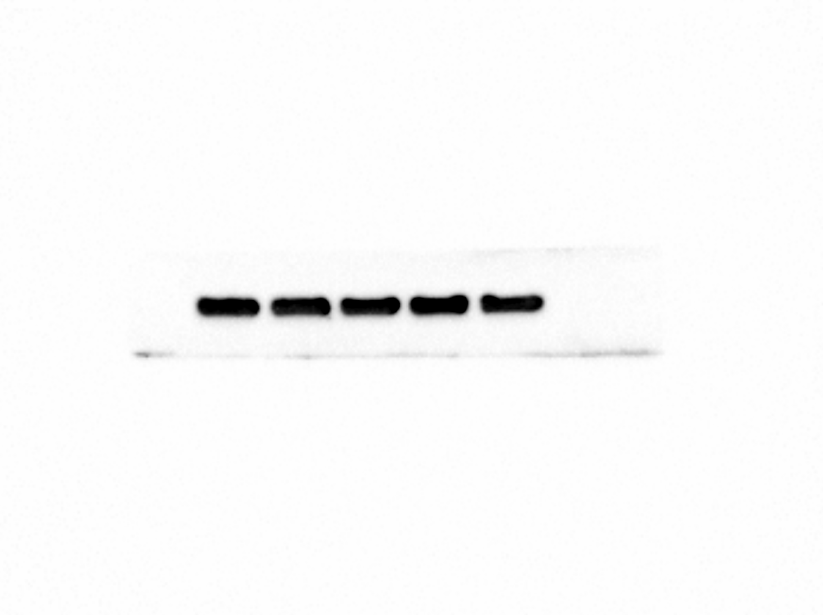


22RV1-NR1D2


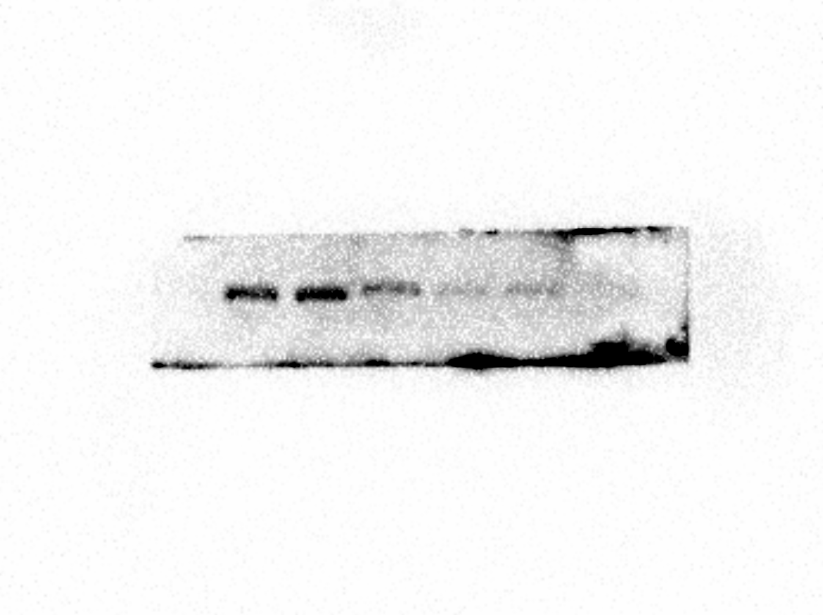


PC3-GAPDH


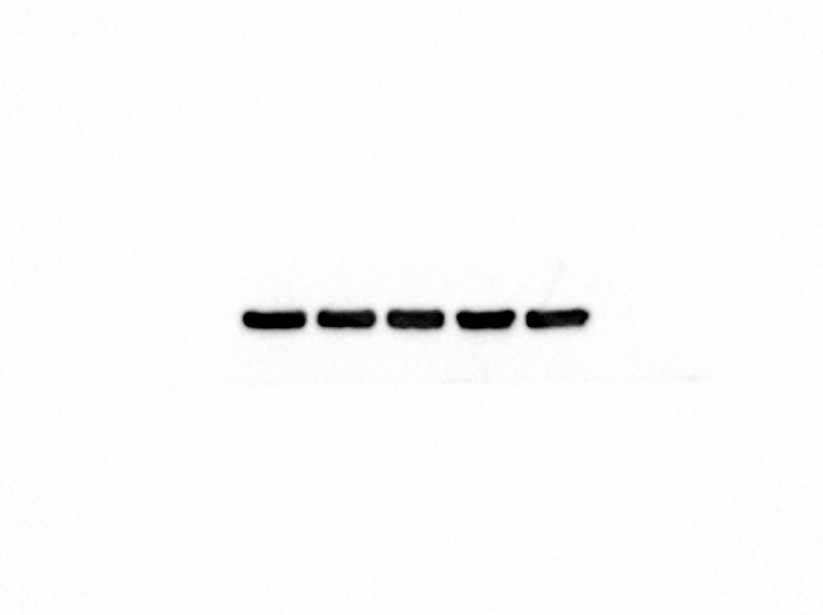


PC3-NR1D2


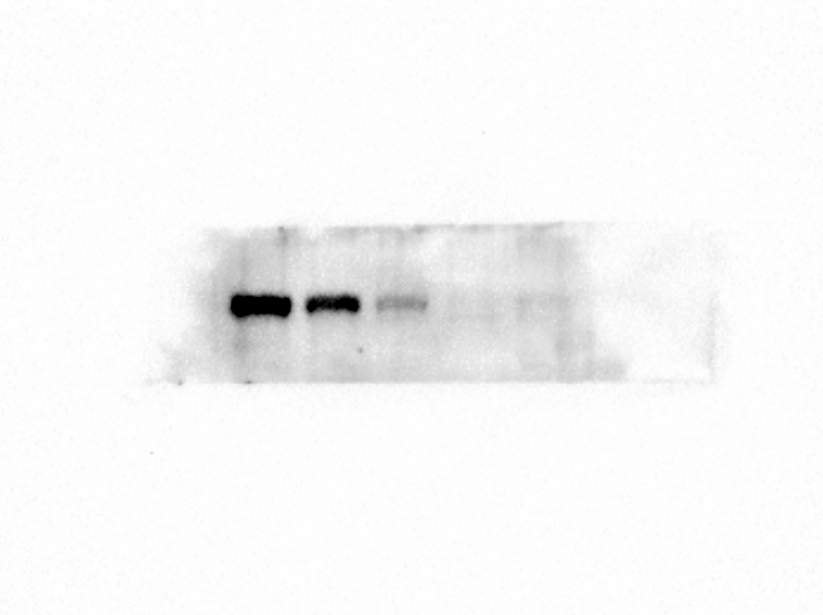


Figure 5J

β-ACTIN


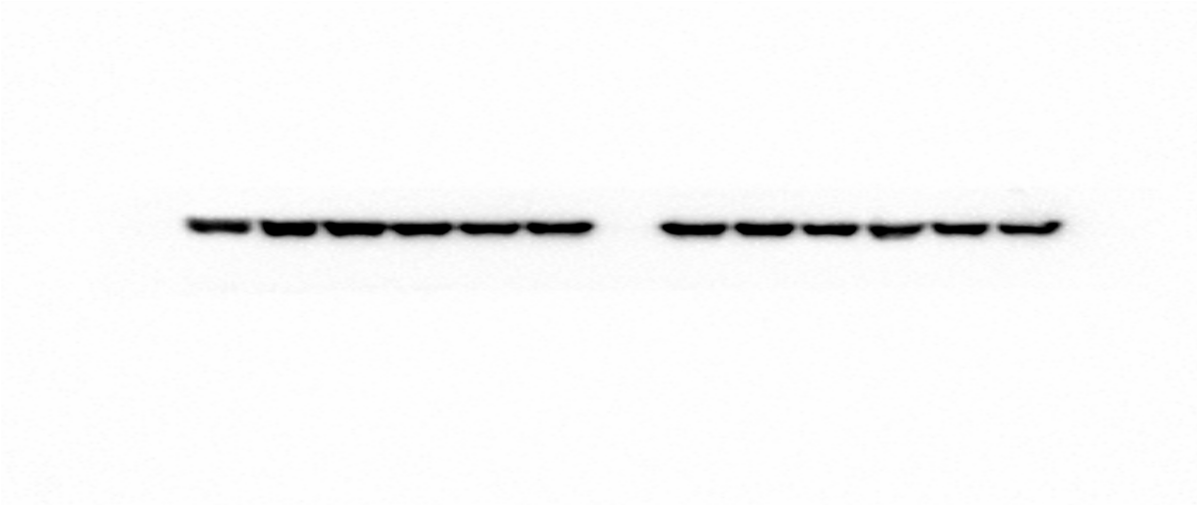


NR1D1


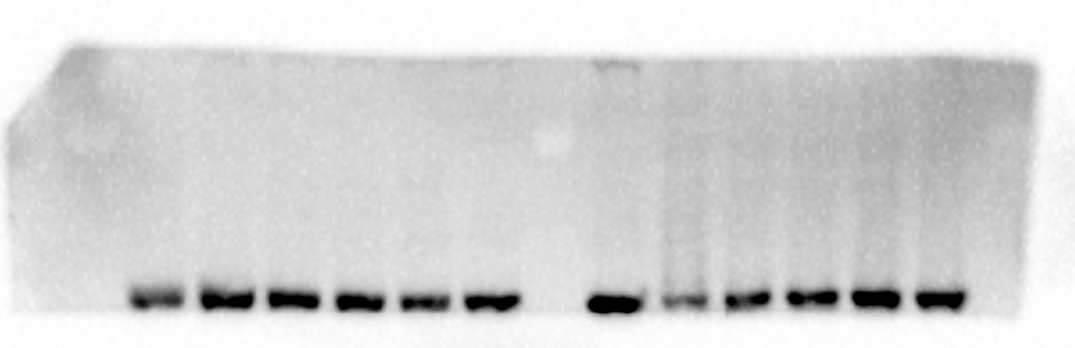


NR1D2


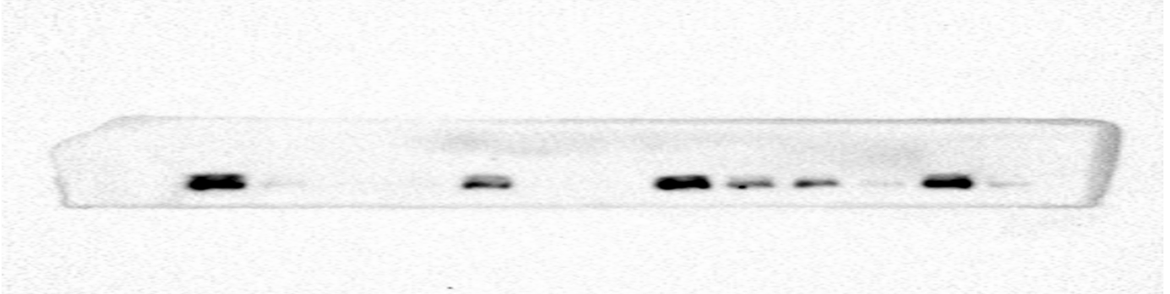


Figure 5M

PC3-REV-ERBα


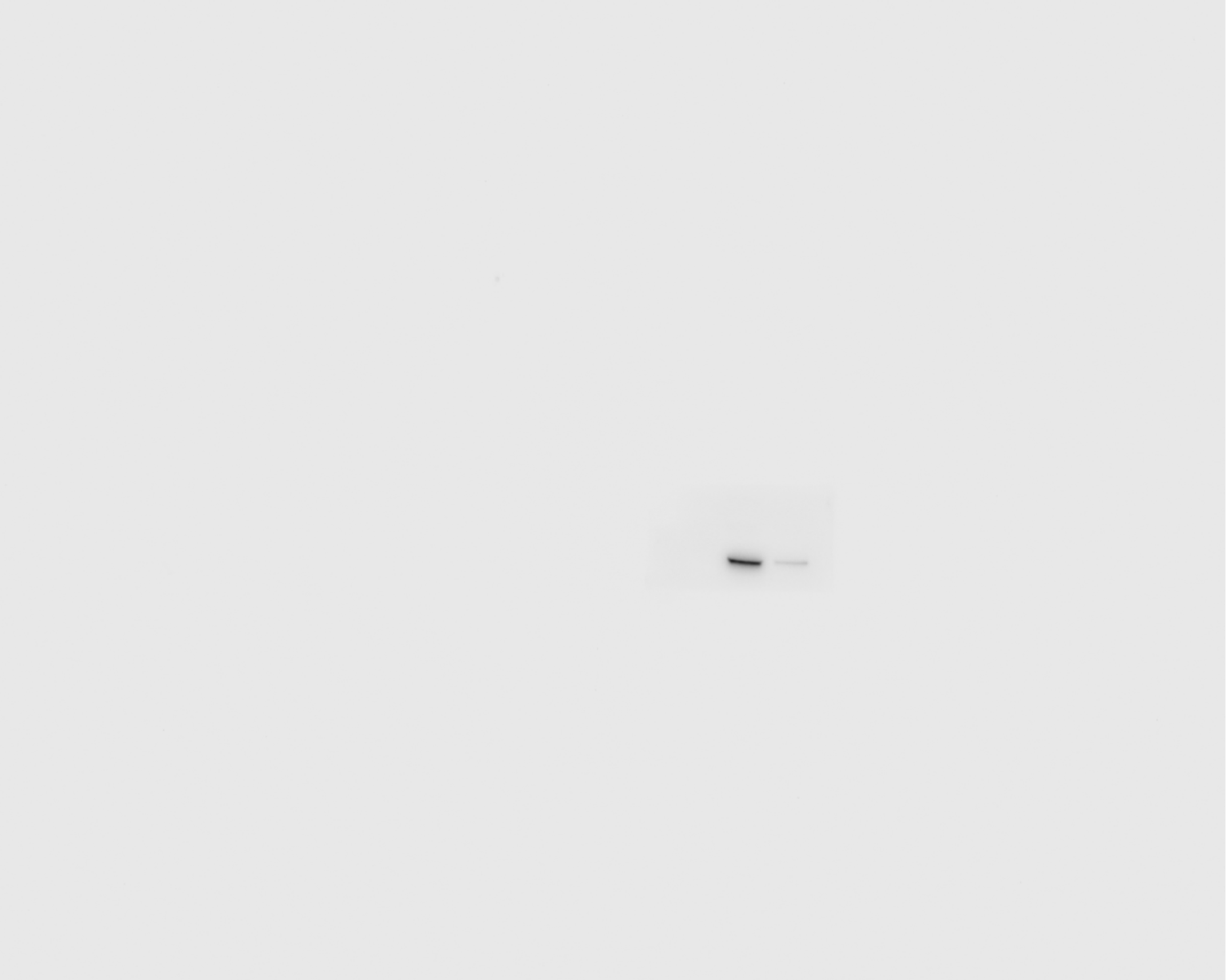


PC3-REV-ERBβ


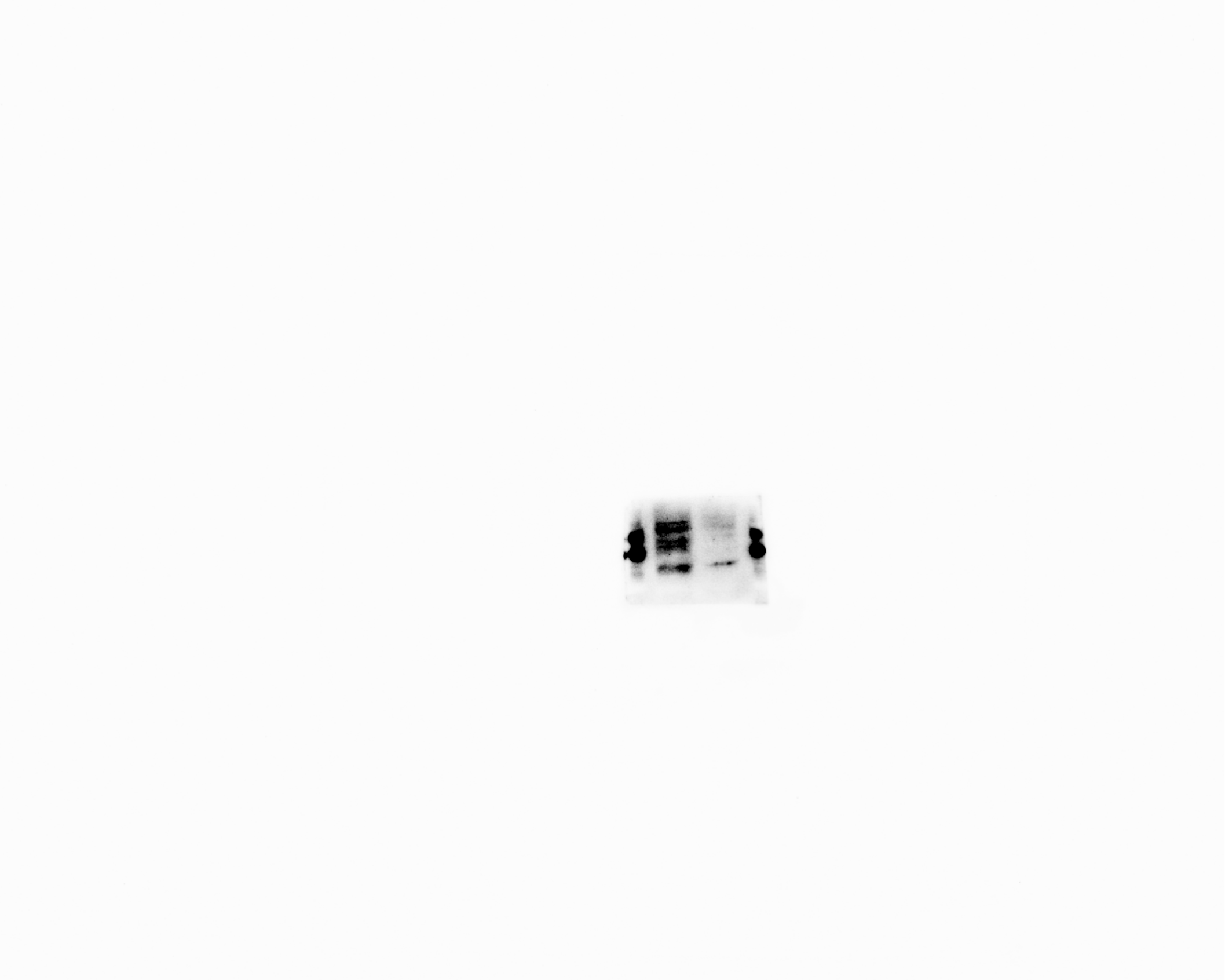


PC3-GAPDH


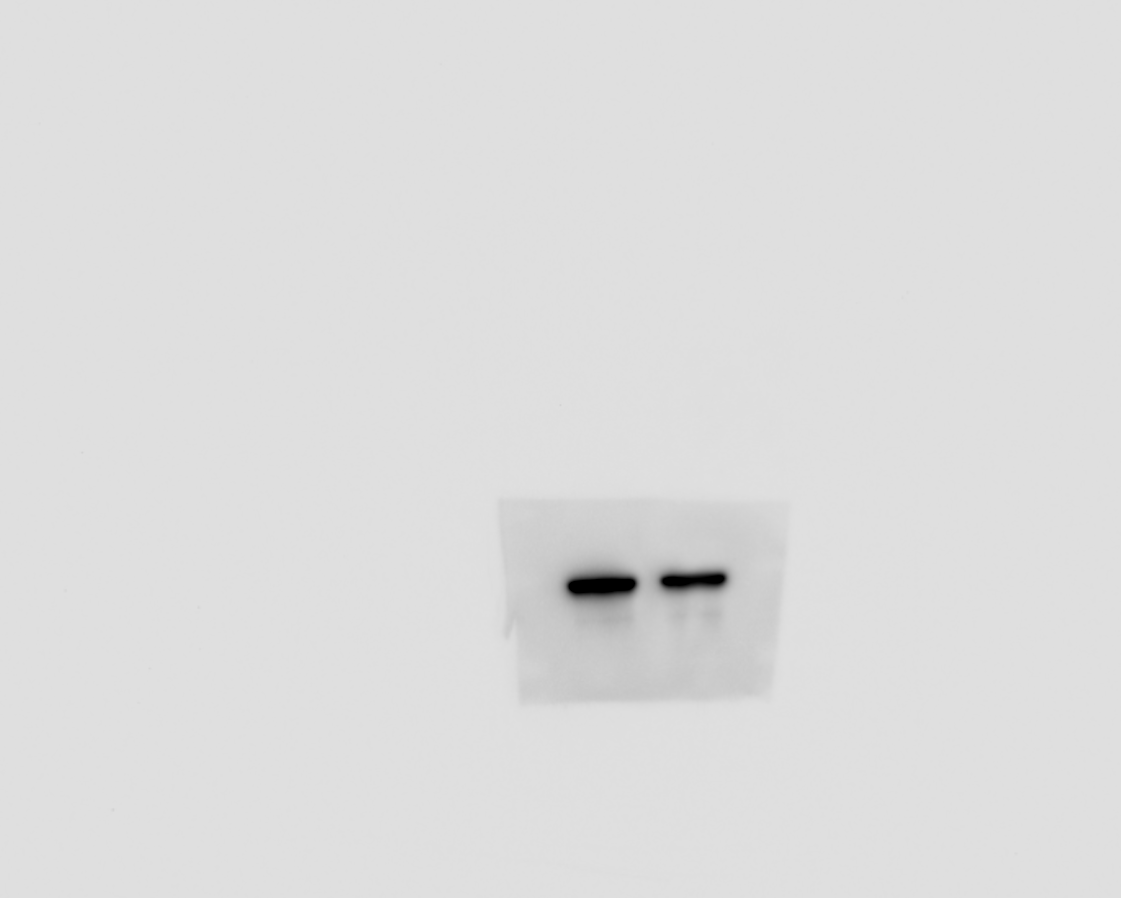


22RV1-REV-ERBα


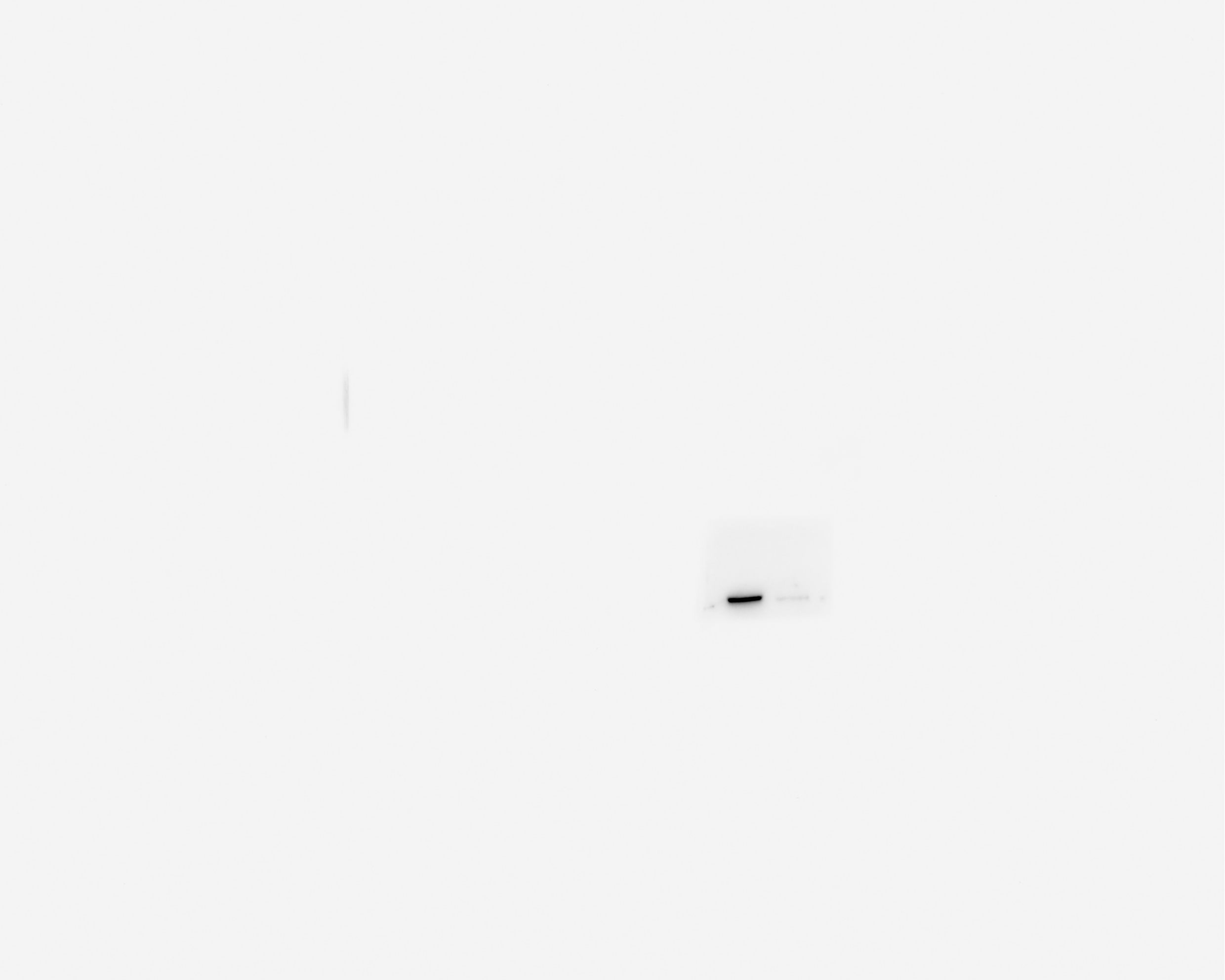


22RV1-REV-ERBβ


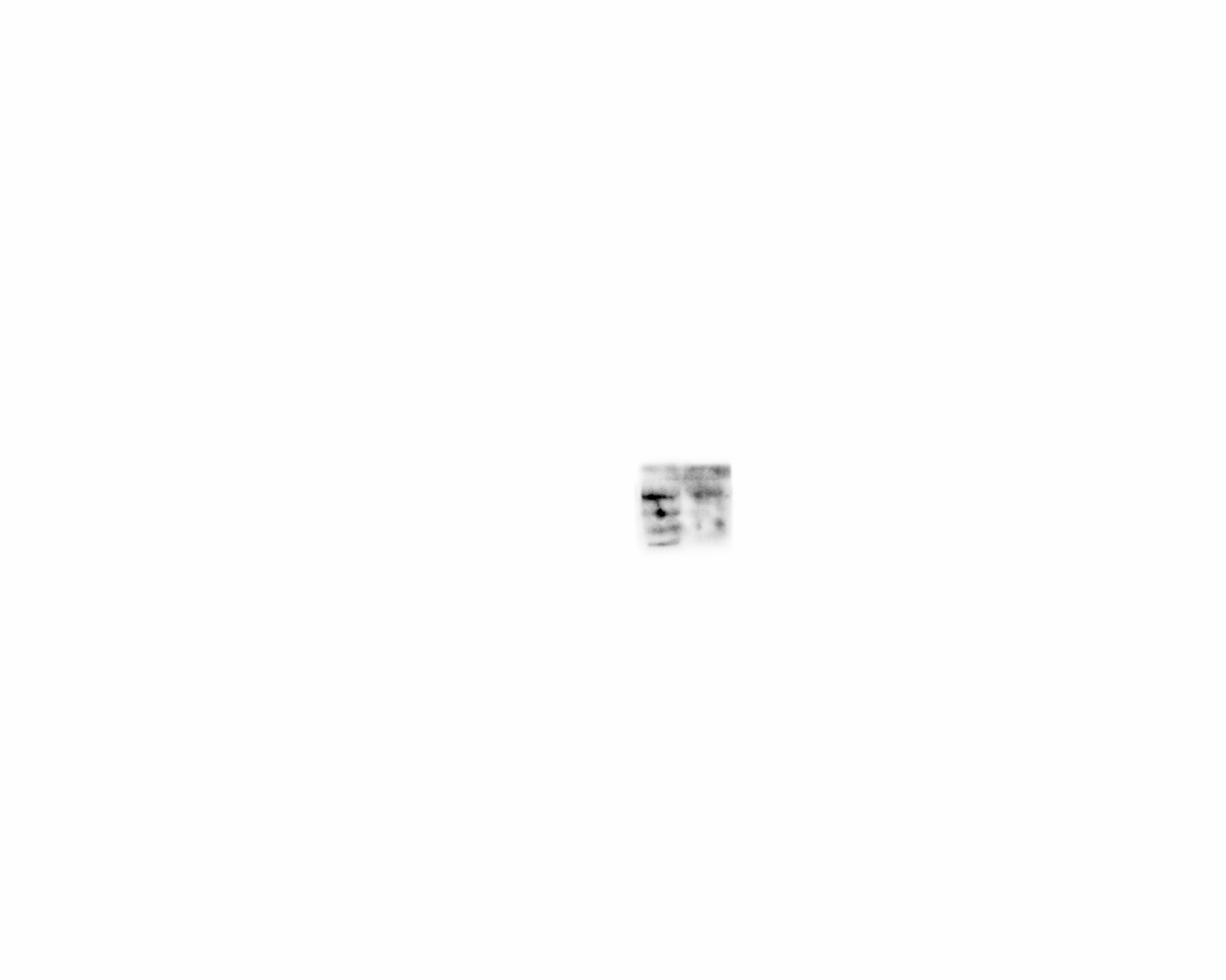


22RV1-GAPDH


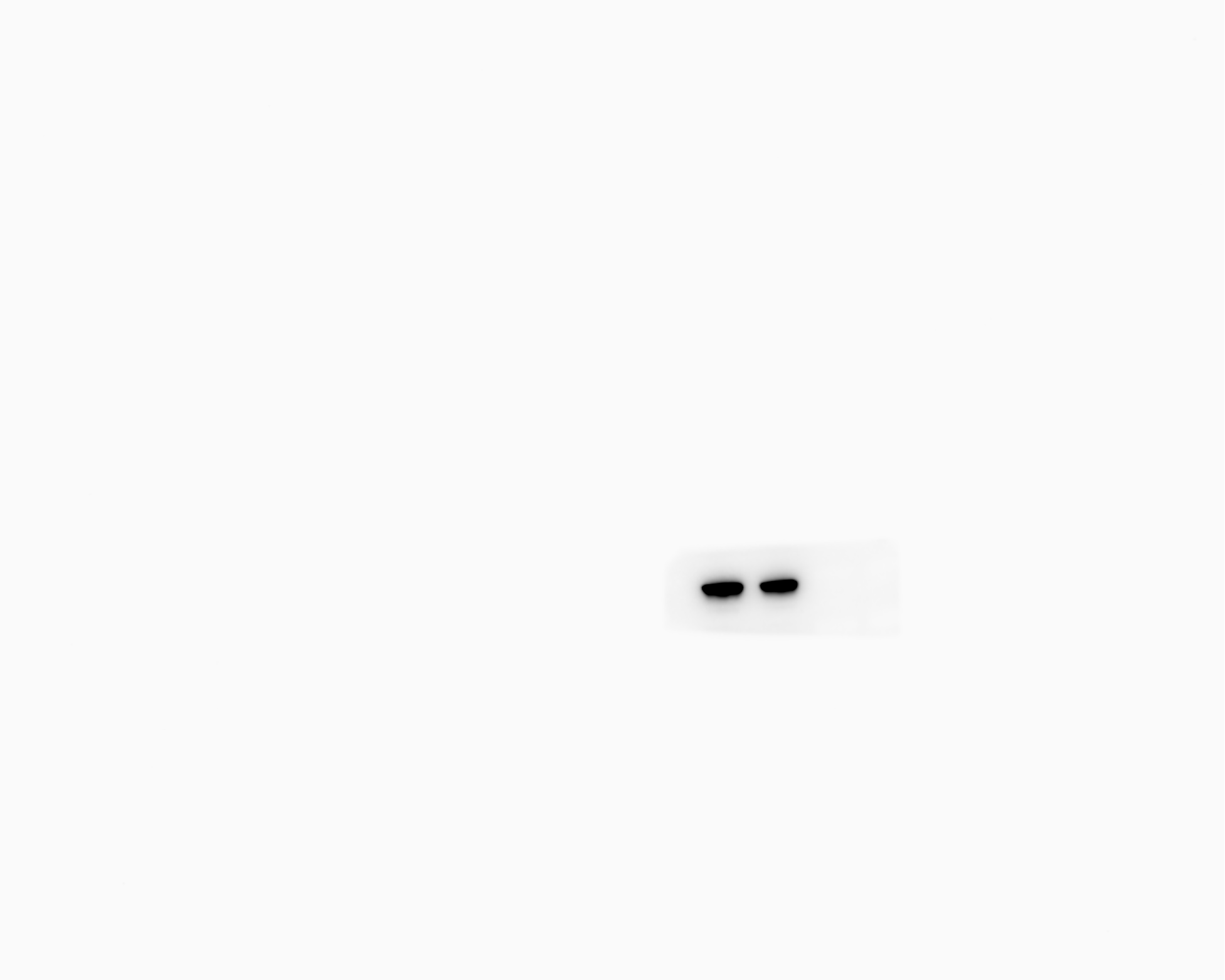


Figure 6C

FOXM1


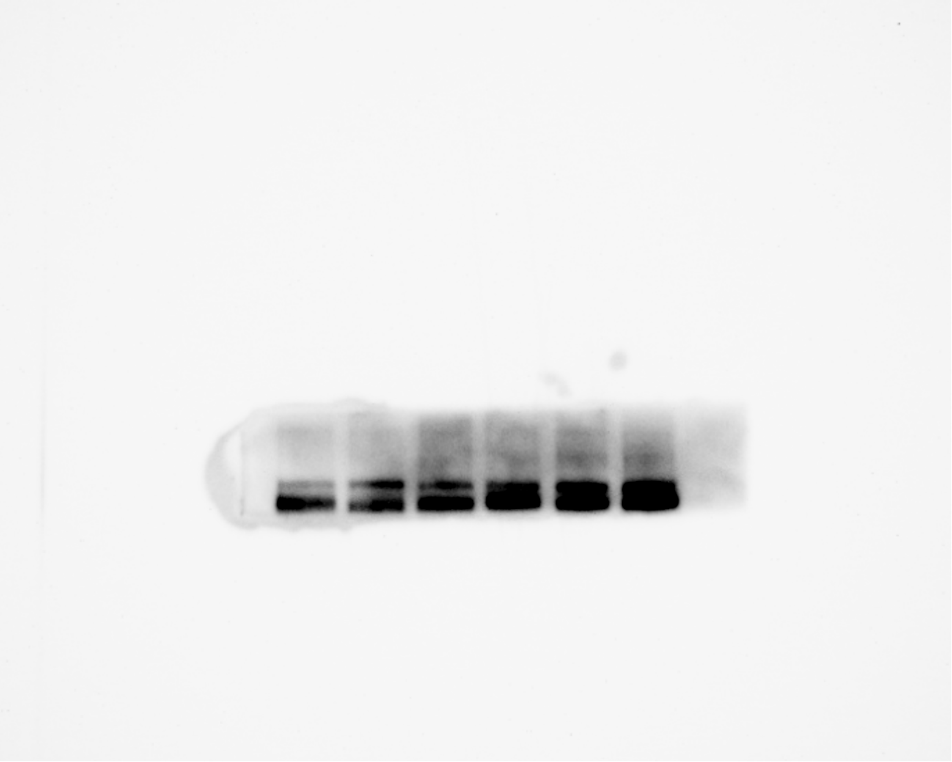


LXRA


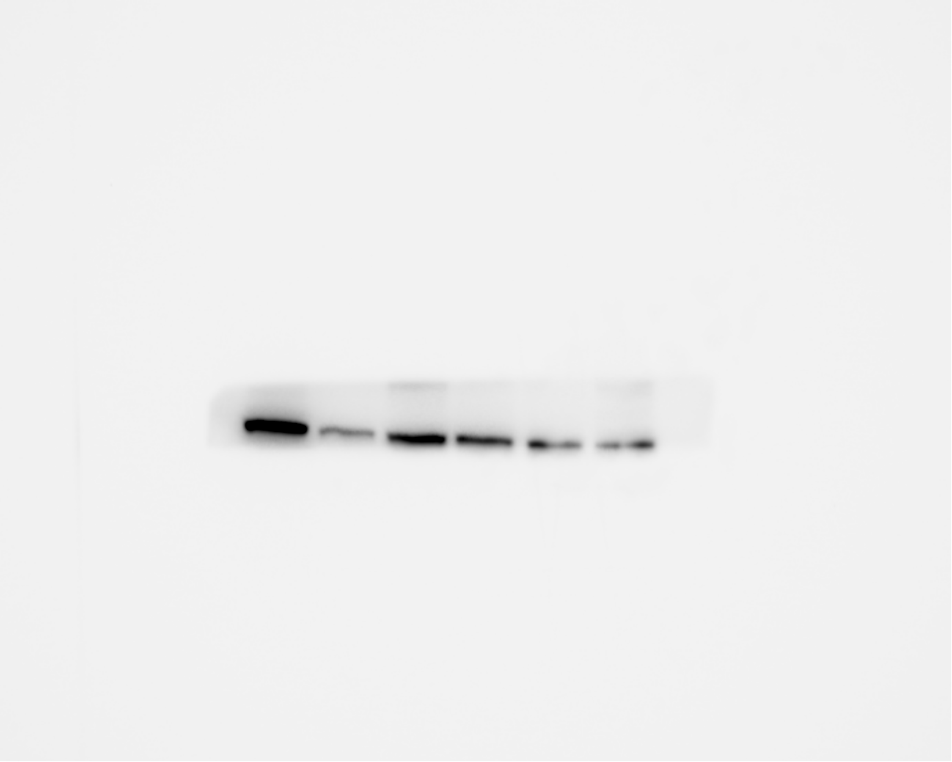


GAPDH


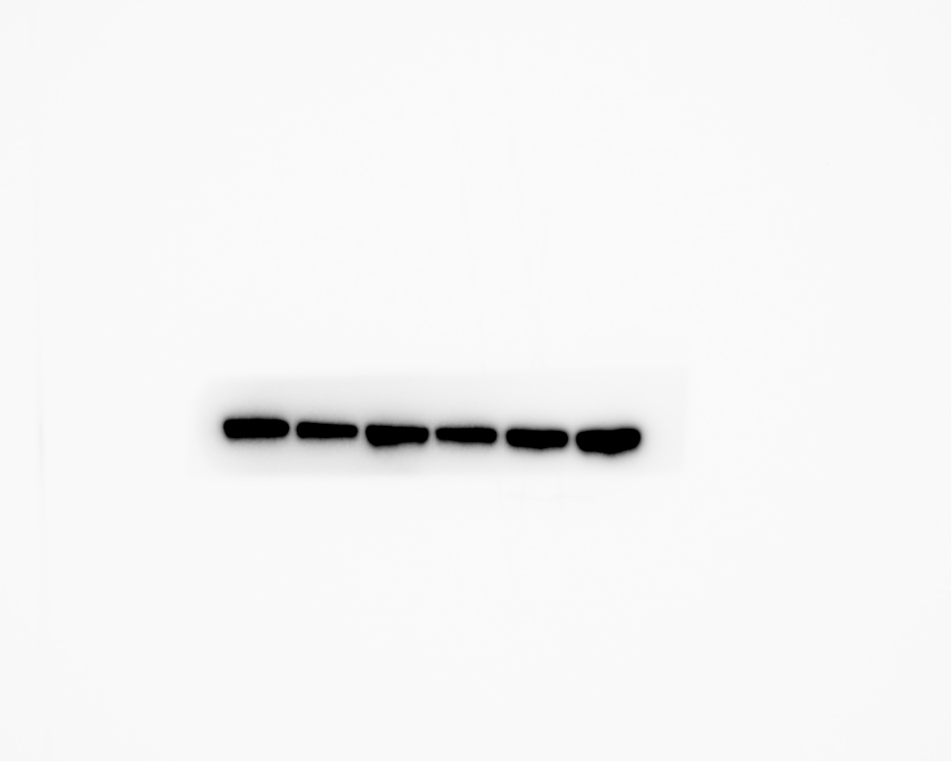


6F

LXRA


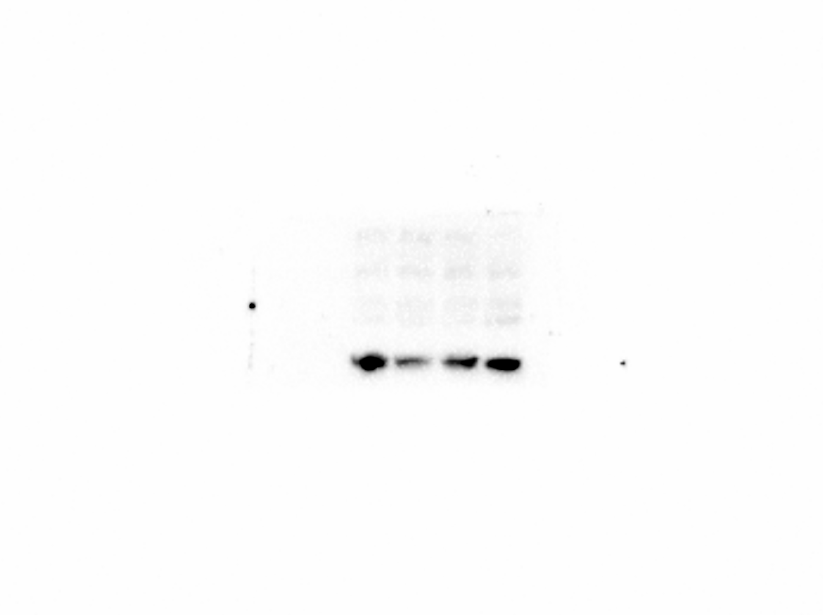


GAPDH


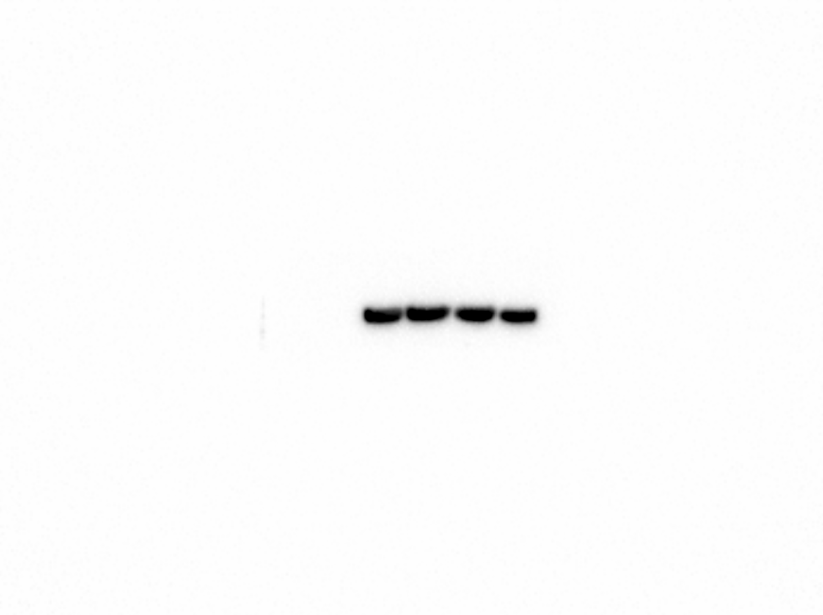


**6G**

22RV1-FOXM1


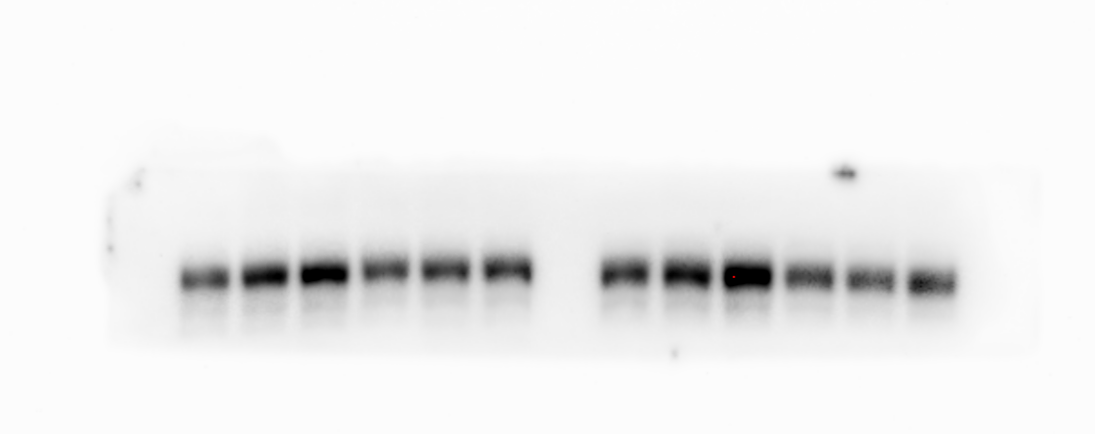


22RV1-GAPDH


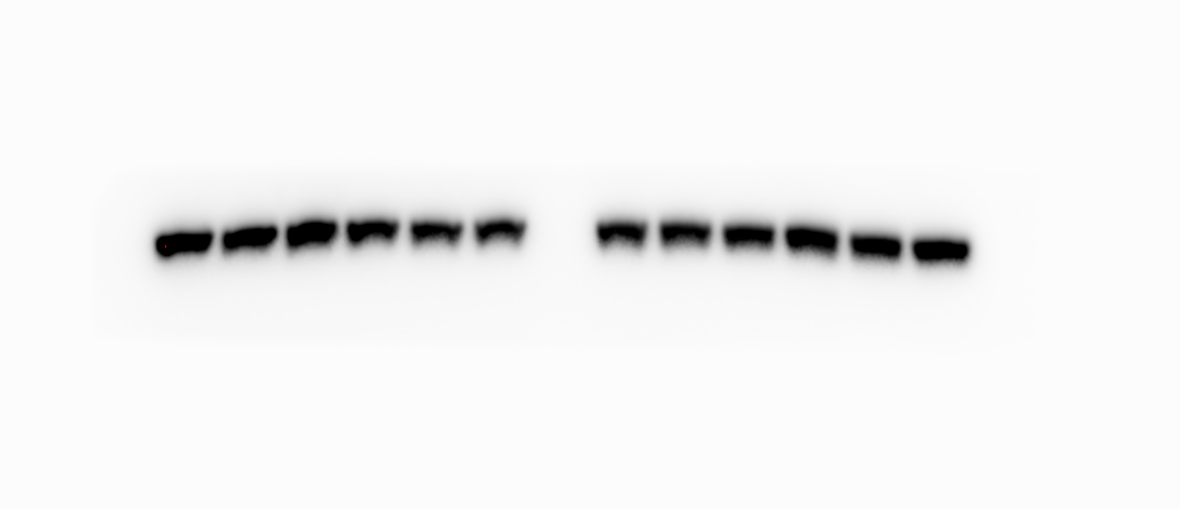


PC3-FOXM1


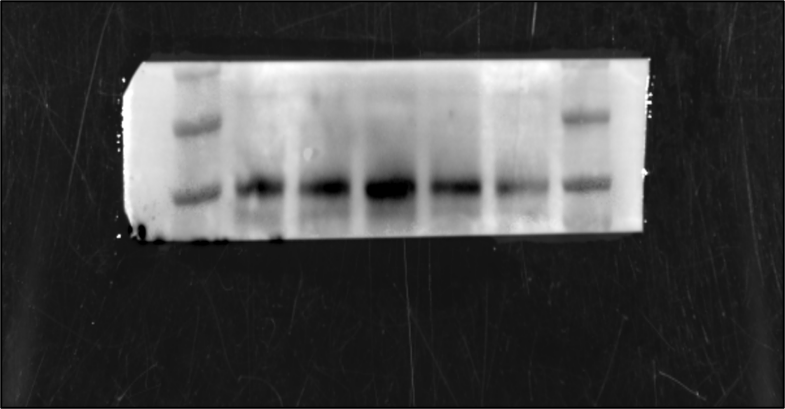


PC3-GAPDH


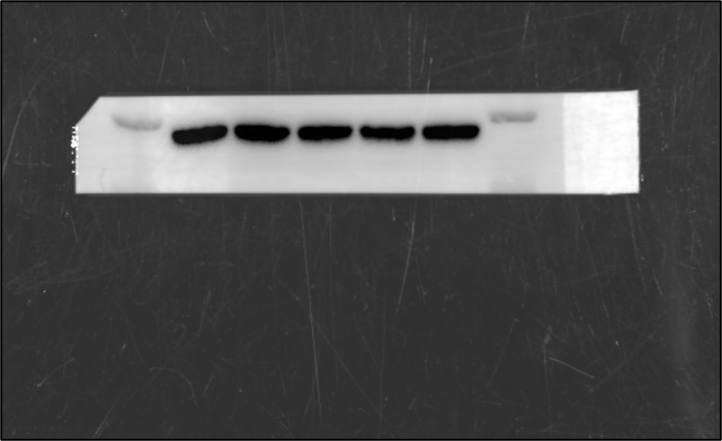


6K

CCNB1


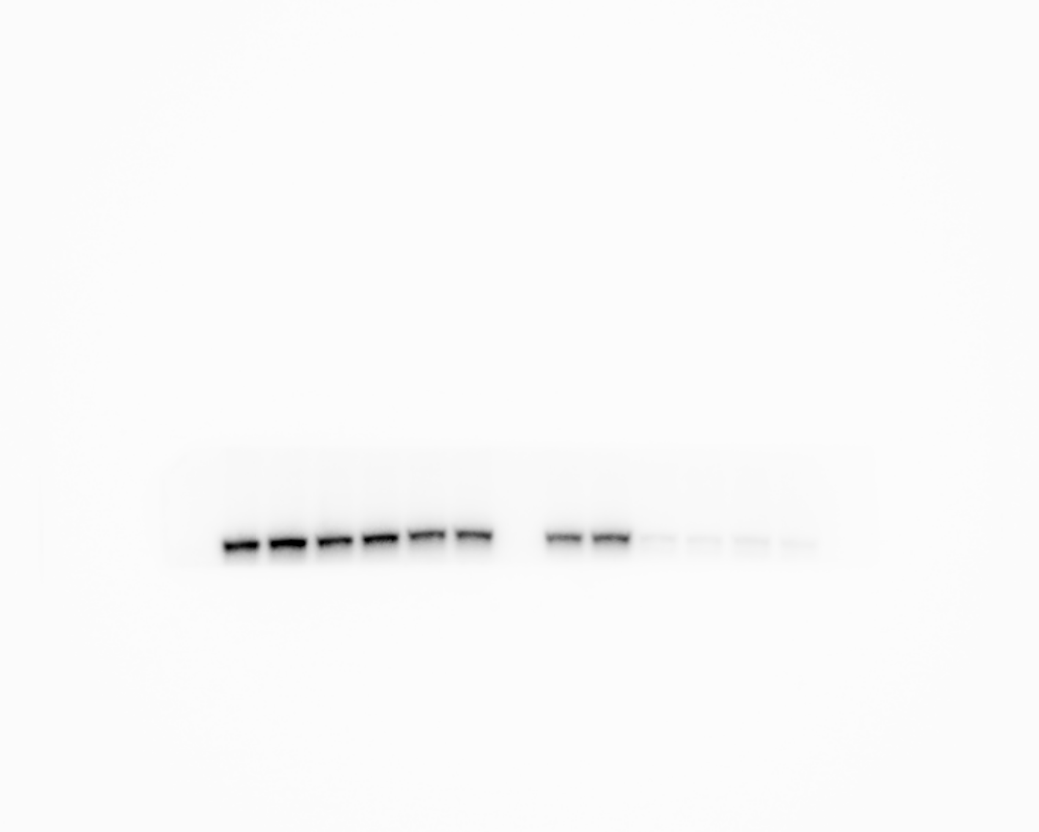


CCNB2


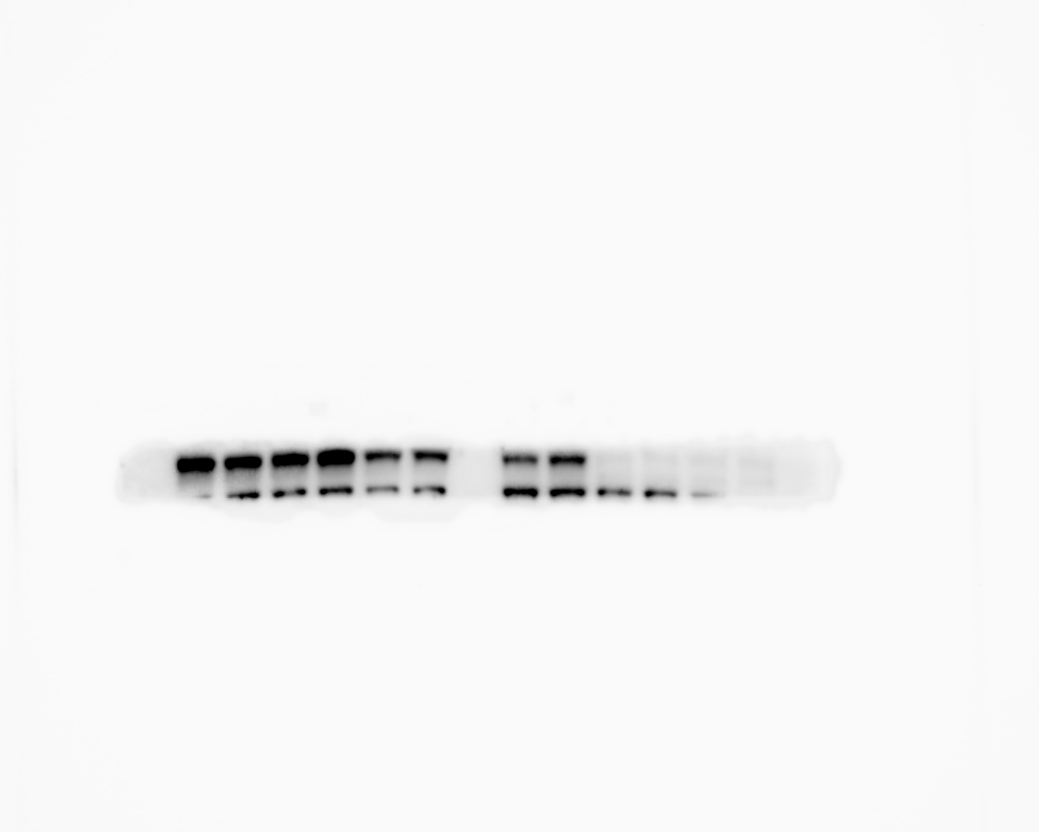


CDK1


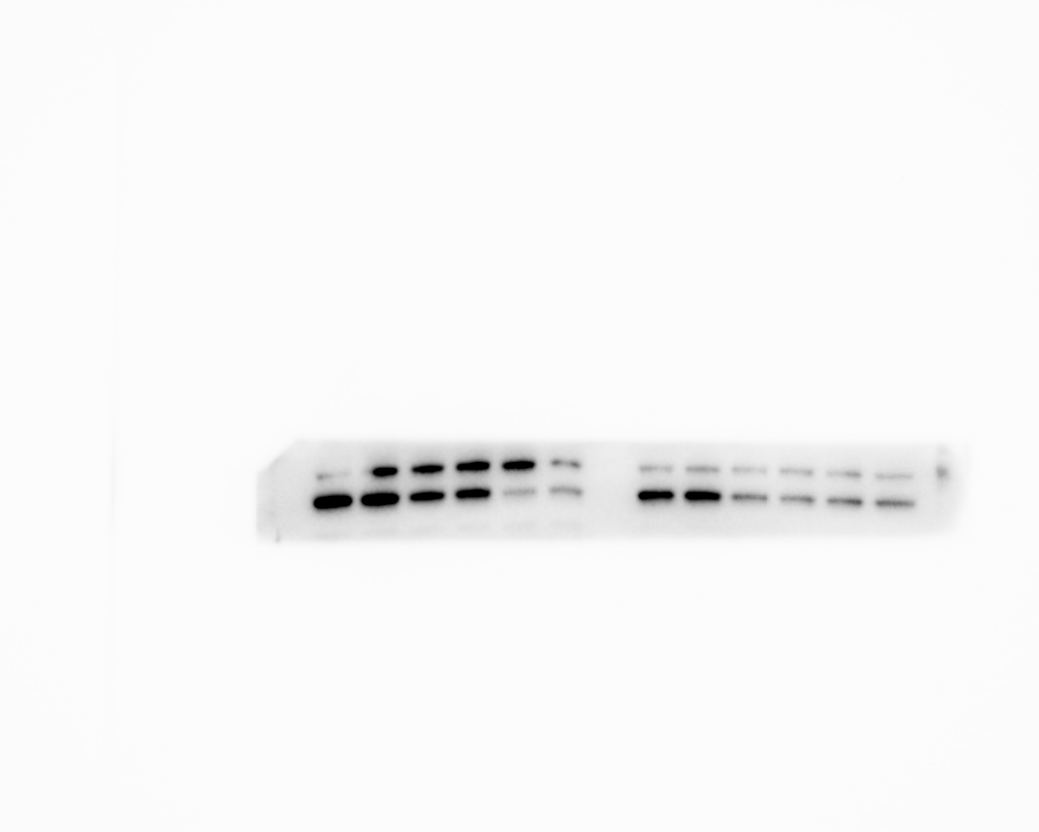


CENPA


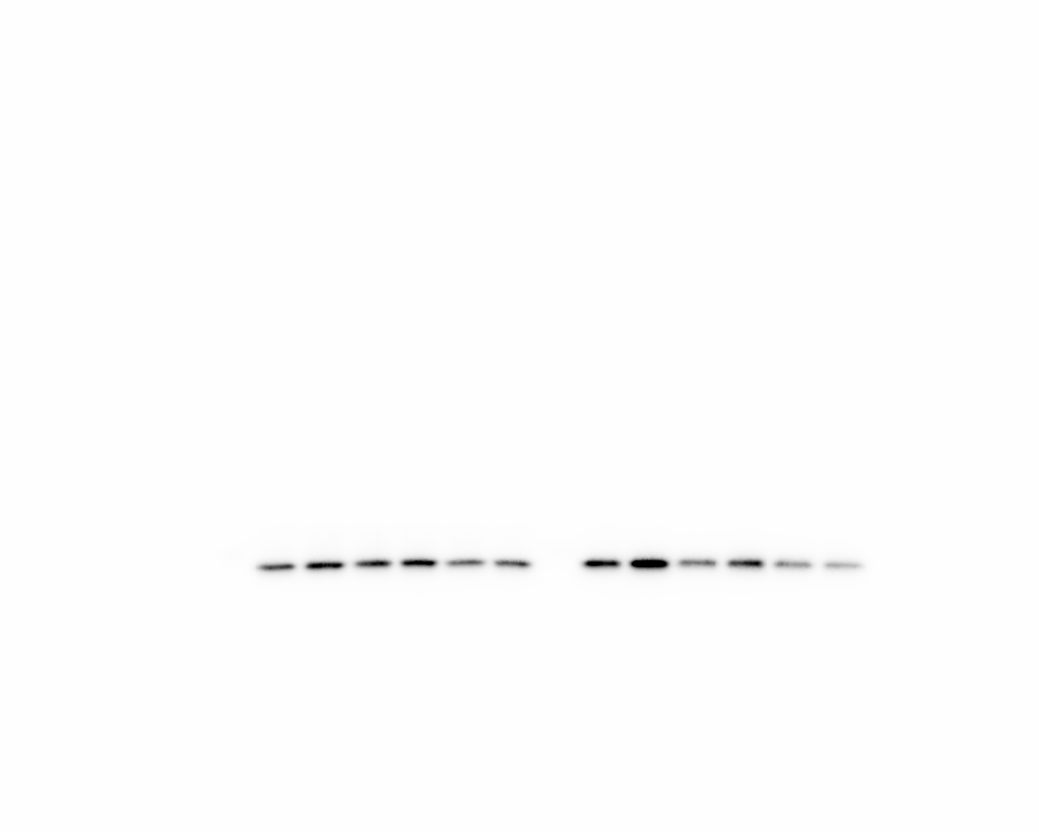


CENPF


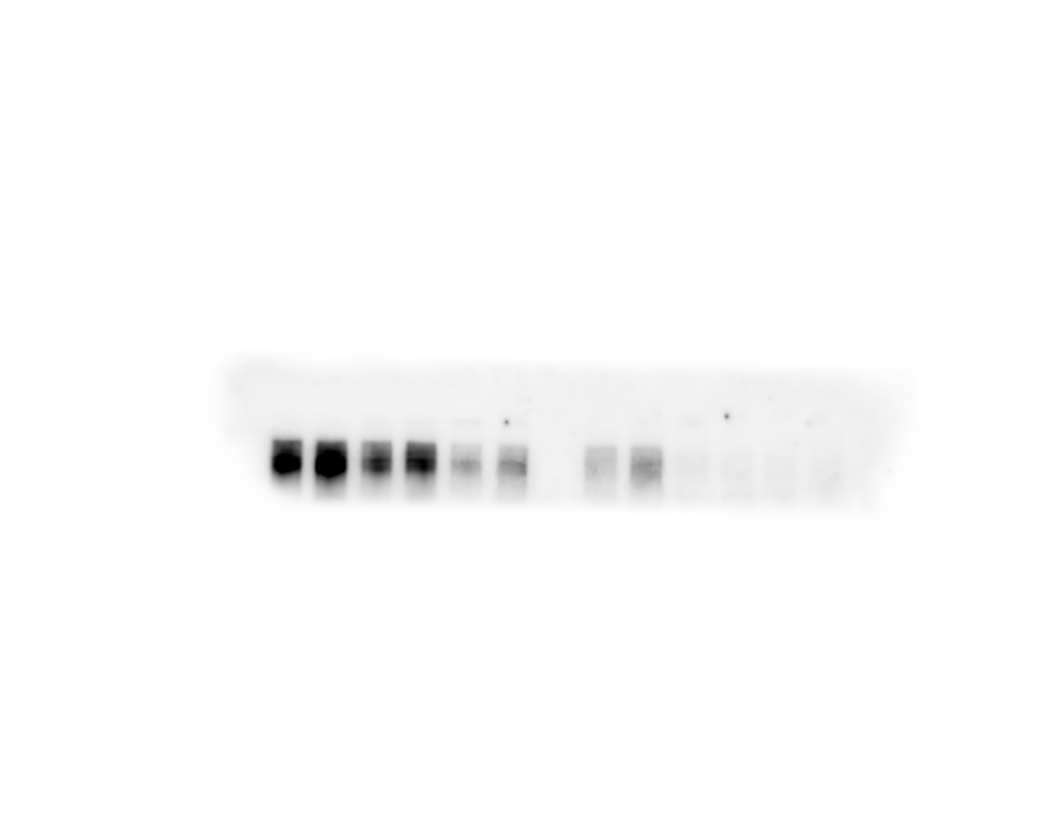


FOXM1


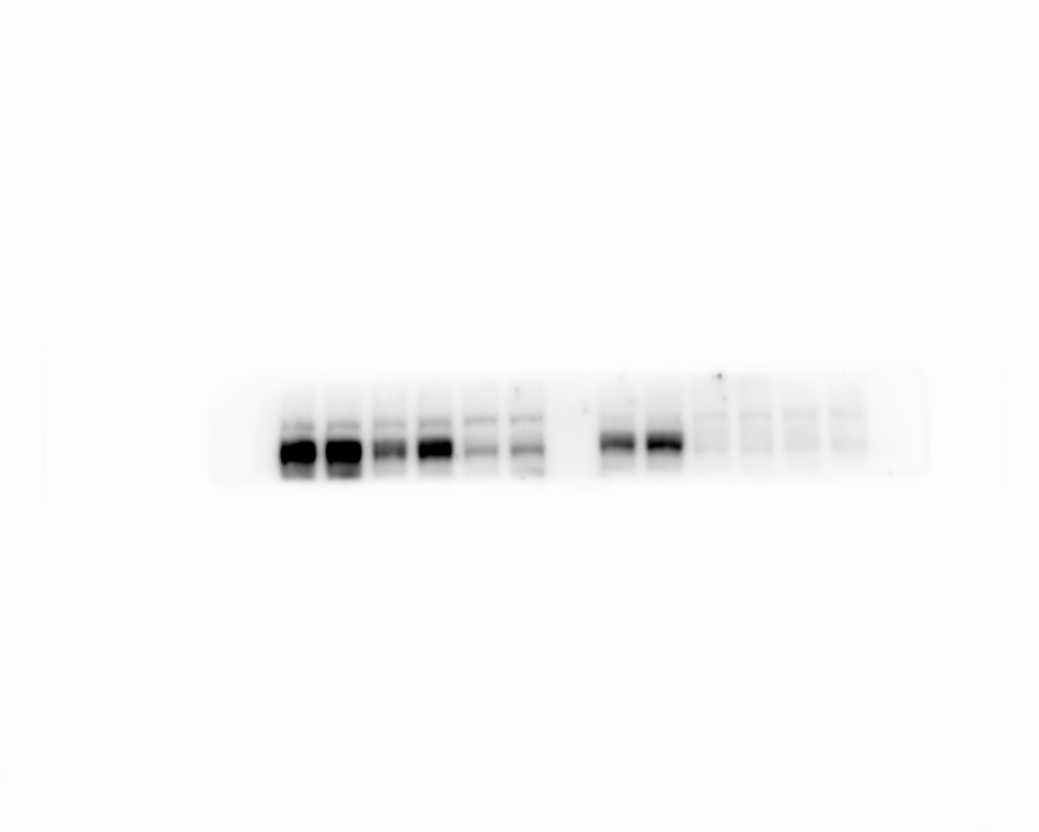


SURVIVIN


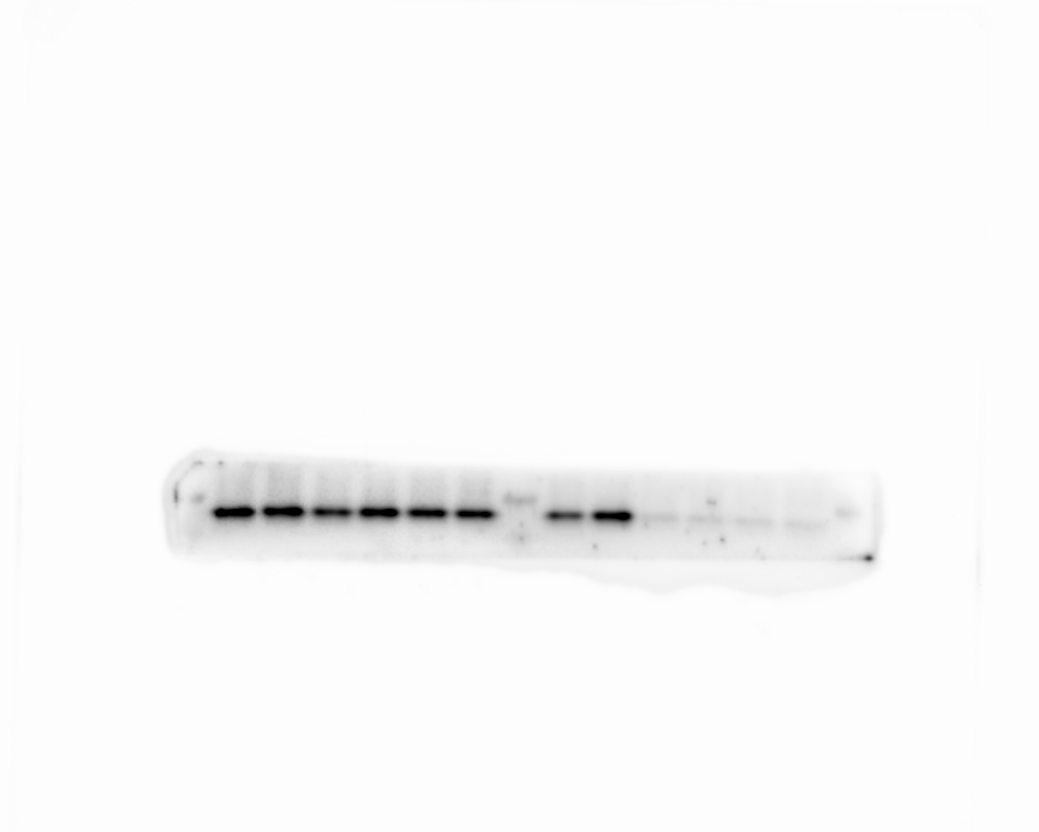


VINCULIN


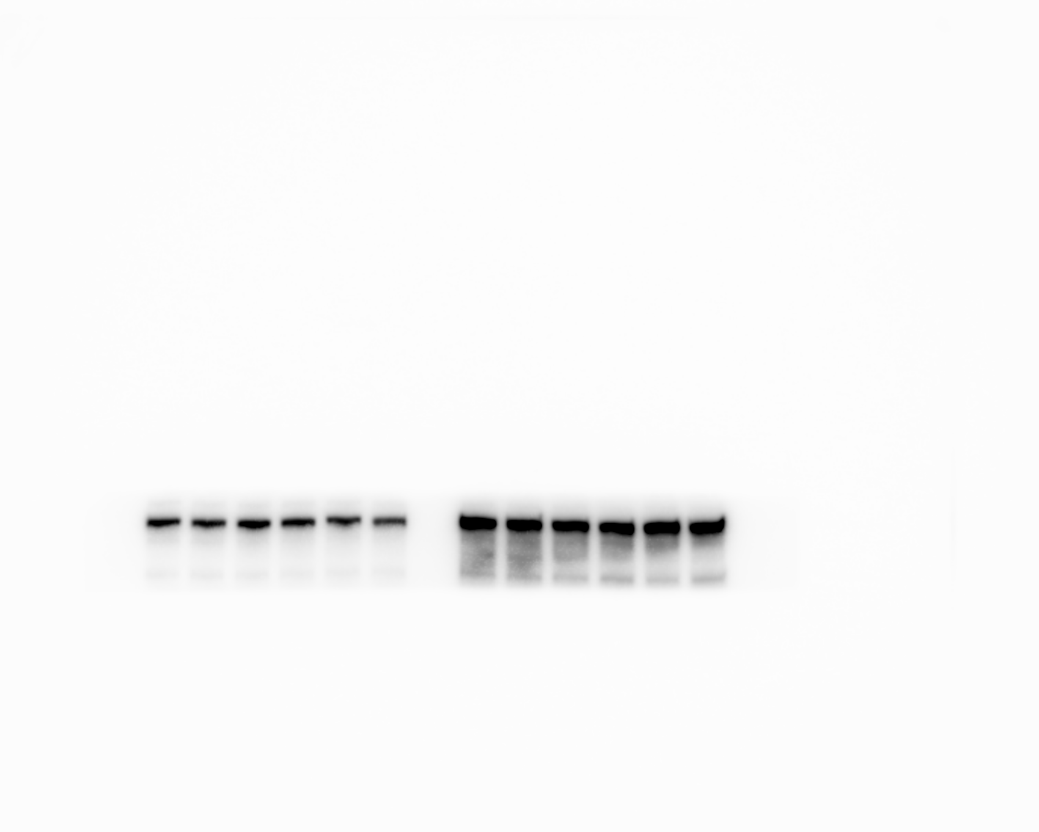


Figure S4D

PC3-BMAL1


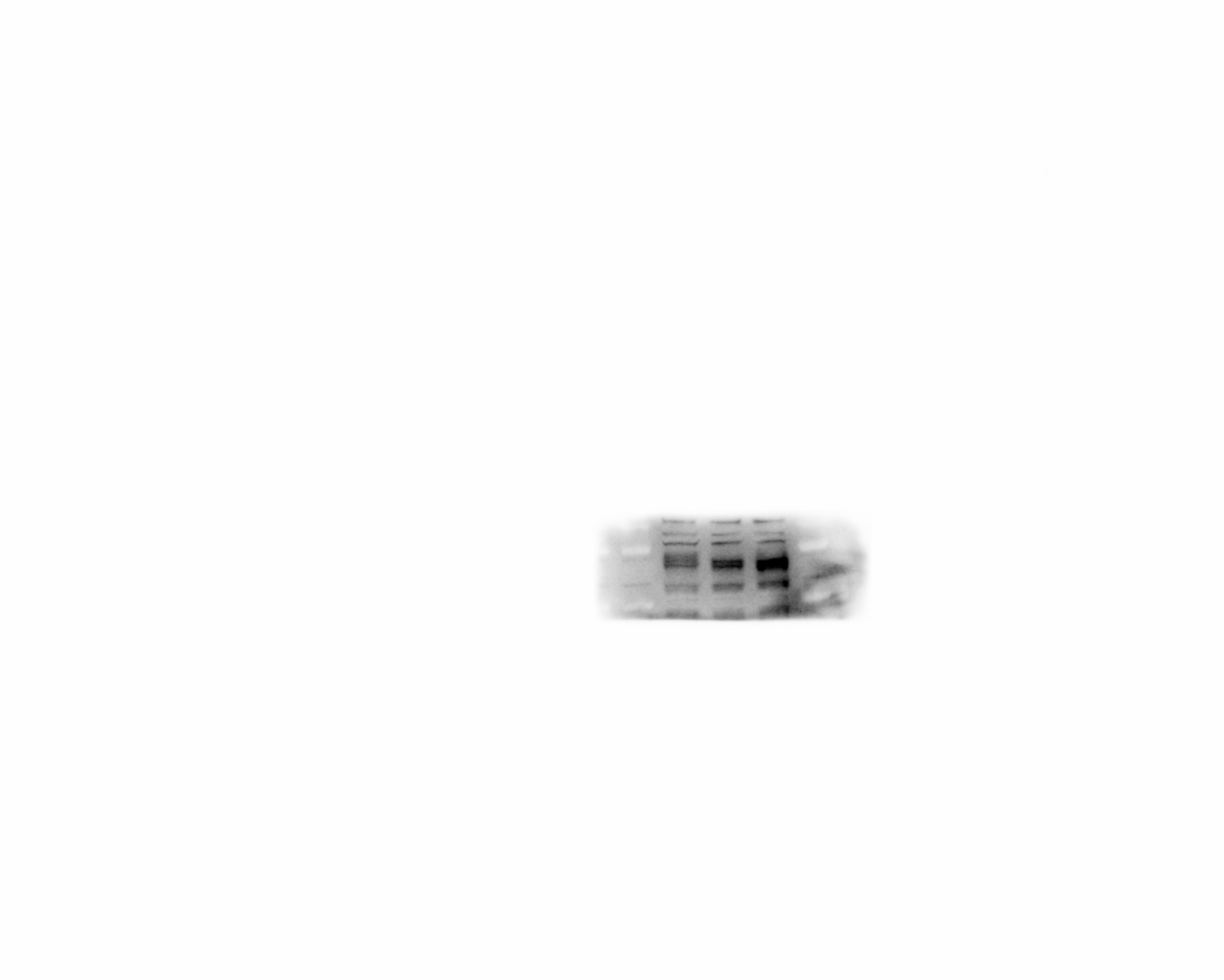


PC3-GAPDH


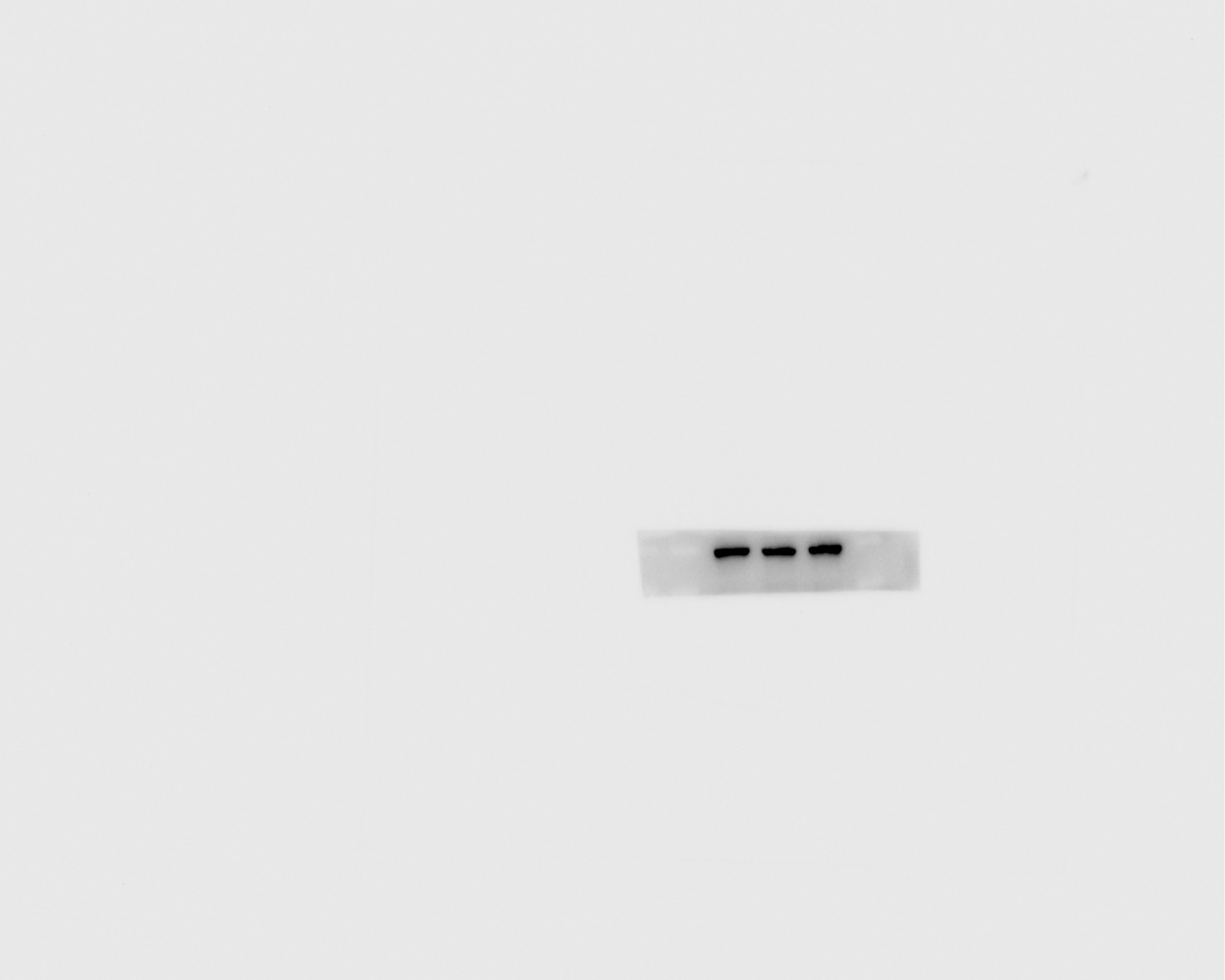


Figure S4E

22RV1-BMAL1


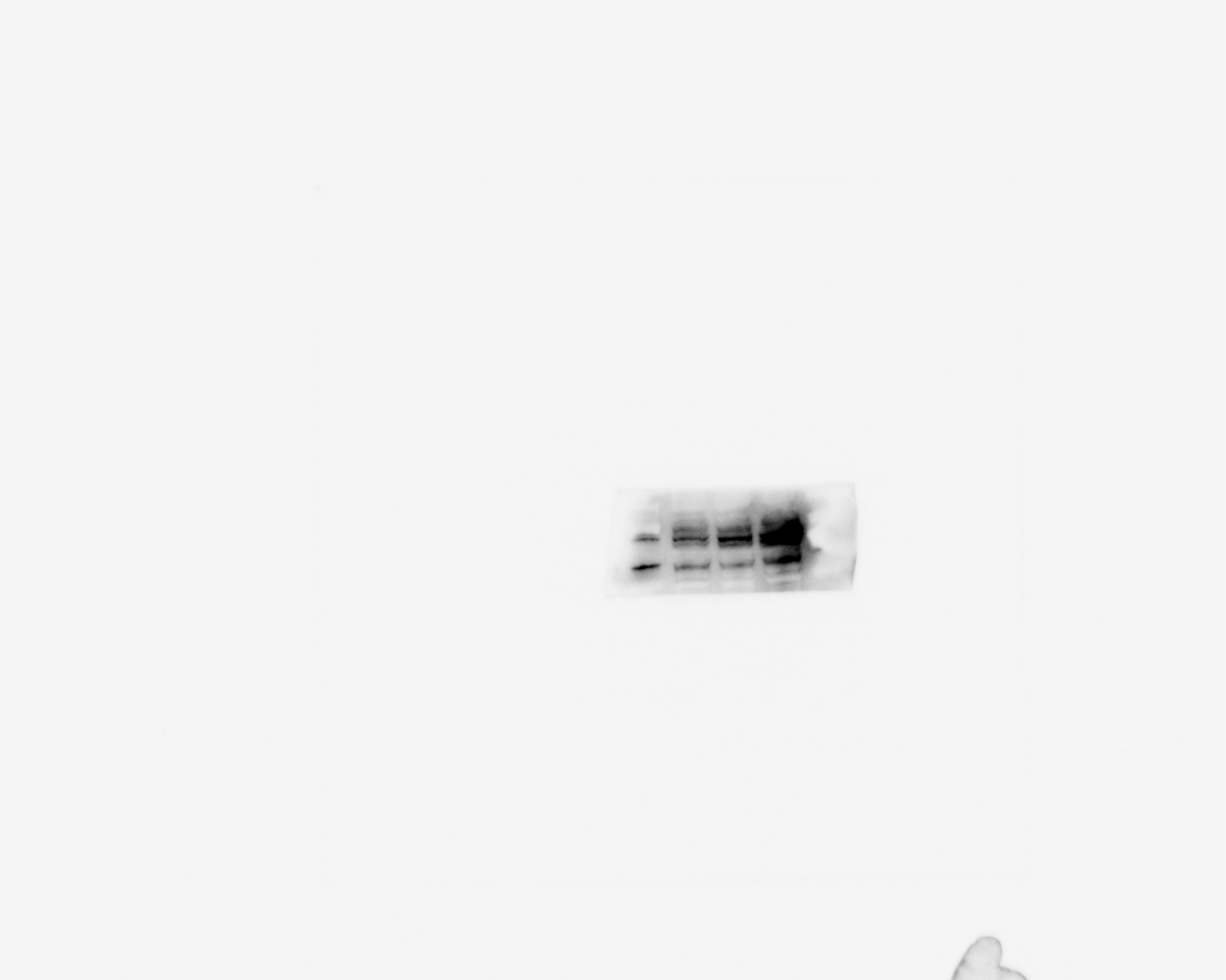


22RV1-GAPDH


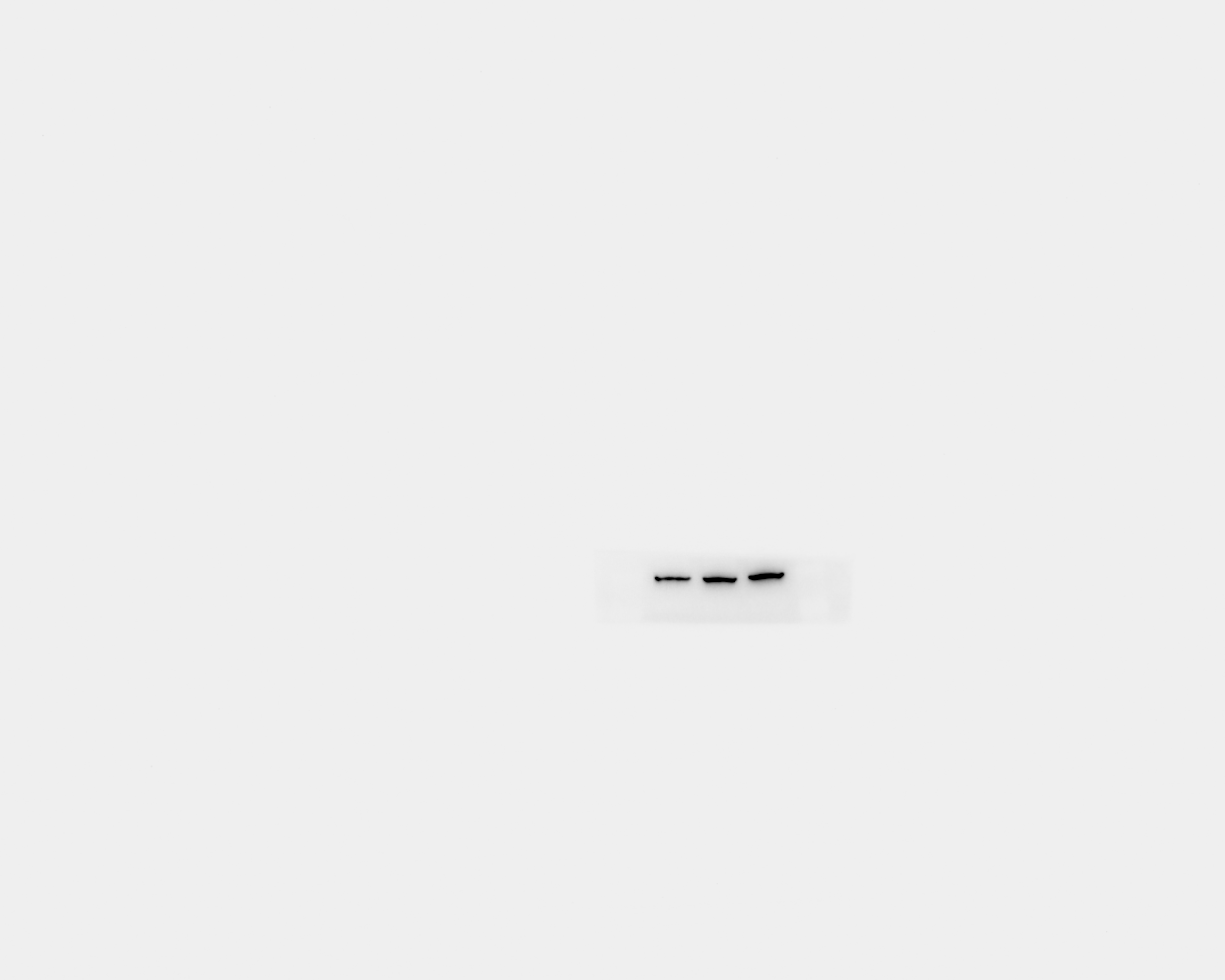

Supplement: Supplementary file 11 — Full and uncropped WB [file 41419_2022_5392_MOESM11_ESM.docx]
